# Sex-specific and developmental expression of *Dmrt* genes in the starlet sea anemone, *Nematostella vectensis*

**DOI:** 10.1186/s13227-015-0013-7

**Published:** 2015-04-25

**Authors:** Nikki G Traylor-Knowles, Eric G Kane, Vanna Sombatsaphay, John R Finnerty, Adam M Reitzel

**Affiliations:** Hopkins Marine Station, Stanford University, 120 Ocean View Blvd, Pacific Grove, CA 93950 USA; Department of Biology, Boston University, 5 Cummington Mall, Boston, MA 02215 USA; Department of Biological Sciences, University of North Carolina at Charlotte, 9201 University City Blvd., Charlotte, NC 28223 USA

**Keywords:** Sex determination, DMRT, *Nematostella vectensis*, Gene expression

## Abstract

**Background:**

The molecular mechanisms underlying sex determination and differentiation in animals are incredibly diverse. The *Dmrt* (doublesex and mab-3 related transcription factor) gene family is an evolutionary ancient group of transcription factors dating to the ancestor of metazoans that are, in part, involved in sex determination and differentiation in numerous bilaterian animals and thus represents a potentially conserved mechanism for differentiating males and females dating to the protostome-deuterostome ancestor. Recently, the diversity of this gene family throughout animals has been described, but the expression and potential function for *Dmrt* genes is not well understood outside the bilaterians.

**Results:**

Here, we report sex- and developmental-specific expression of all 11 *Dmrts* in the starlet sea anemone *Nematostella vectensis*. Nine out of the eleven *Dmrts* showed significant differences in developmental expression, with the highest expression typically in the adult stage and, in some cases, with little or no expression measured during embryogenesis. When expression was compared in females and males, seven of the eleven *Dmrt* genes had significant differences in expression with higher expression in males than in females for six of the genes. Lastly, expressions of two *Dmrt* genes with differential expression in each sex are located in the mesenteries and into the pharynx in polyps.

**Conclusions:**

Our results show that the phylogenetic diversity of *Dmrt* genes in *N. vectensis* is matched by an equally diverse pattern of expression during development and in each sex. This dynamic expression suggests multiple functions for *Dmrt* genes likely present in early diverging metazoans. Detailed functional analyses of individual genes will inform hypotheses regarding the antiquity of function for these transcription factors.

**Electronic supplementary material:**

The online version of this article (doi:10.1186/s13227-015-0013-7) contains supplementary material, which is available to authorized users.

## Background

The pathways involved in metazoan sex determination are diverse including transcriptional regulation, post-transcriptional modifications, and hormone synthesis. However, distantly related metazoan phyla (e.g., arthropods, nematodes, and chordates) utilize a member of the doublesex and mab-3 related transcription factor (*Dmrt*) family to regulate sexual determination and/or the development of sexual dimorphism [1,2]. This gene family is defined by its presence of a highly conserved DNA binding motif, the DM domain, which is characterized by cysteine-rich, interlaced zinc fingers [3]. Additionally, some *Dmrt* genes have an additional conserved region termed the DMA domain, which may be involved in neurogenesis [4,5], although the specific functions are unknown.

In bilaterians, one or more DMRT proteins play a role in the development of male-specific characteristics [6-11]. Broadly, *Dmrt* genes contribute towards sex-specific characteristics and can promote the phenotypes of either males or females. Evidence from various species has suggested that two characteristics of *Dmrt* genes correlate with sex-biased expression: loss or absence of the DMA domain and differential splicing. For example, in *Drosophila melanogaster*, the *Dmrt* gene called *doublesex* (*dsx*) is named because it plays a role in determining both males and females [12]. *dsx* from *Drosophila* (and other insects) lacks a DMA domain (like DMRT1 in mammals and mab-3 in *C. elegans*) and undergoes differential splicing in each sex. Male and female fruit flies express *dsx* transcripts but produce different isoforms via alternate splicing (DSX^M^ and DSX^F^) [12]. Splicing of *Dmrt* transcripts appears to be common in many animals; however, the function of splice variants outside of sex determination in studied insects remains largely unstudied [13].

Given the variation among bilaterian animals in the function of *Dmrt* genes, including sex determination and neurogenesis, data from outgroups to the bilaterians (e.g., ctenophores, cnidarians) are needed to clarify potential ancestral functions in the animal lineage. In the reef building coral *Acropora millepora*, one *Dmrt* gene (named DM 1, evolutionarily related to other *Dmrt* genes from cnidarians and *Trichoplax* and most closely related to *Nematostella vectensis Dmrt* A) [14] was highly expressed in the tips of adult corals during the reproductive season [15]. *A. millepora* is a simultaneous hermaphrodite; thus, increased expression of this single *Dmrt* gene could not be assigned to a particular sex. AmDM 1 contained a DMA domain and the transcript showed two splice variants, suggesting a potential role for post-transcriptional modification like that observed for *dsx* in *D. melanogaster* [15]*.* Subsequent to this study, gene discovery and phylogenetic analyses from *A. millepora* have shown that this coral species contains at least five additional *Dmrt* genes that have not yet been characterized [14]. *Dmrt* genes have been identified in other early diverging phyla [14,16], but expression or function has not been characterized.

*N. vectensis* is a cnidarian amenable to studying *Dmrt* genes in development, sexual determination, and/or differentiation in an early diverging species due to accessibility of developmental stages and adult gonochorism [17]. *N. vectensis* has 11 *Dmrt* genes [5,14], the most of any animal species yet described, which have generally unclear orthology to bilaterian *Dmrt* genes suggesting independent expansion of this gene family in cnidarians. Switching of sexes or hermaphroditism has not been documented in this species suggesting that sex determination likely has a genetic component [17]. In adult females, oocytes develop within the mesenteries, so that all stages of oogenesis can potentially be present [18]. In adult males, spermary development occurs within the mesenteries in tight bundles [19].

We report the protein models and domain architecture for the 11 *N. vectensis* proteins. To begin to characterize the role of *Dmrt* genes in *N. vectensis*, gene expression was determined over a developmental series and in sexually mature males and females. We found significant differences in gene expression of *Dmrt* genes between males and females, consistent with a potential role in sex determination or differentiation. Additionally, we found differences in expression during development. This report presents the first evidence of differences in *Dmrt* gene expression between females and males in a cnidarian.

## Methods

### Gene and protein models

The 11 *Dmrts* which were previously identified in Bellefroid *et al.* 2013 [16] and Wexler *et al*. [14] were blasted using blastn or tblastn against the full DNA sequence scaffold identified on the Joint Genome Institute genome portal (http://genome.jgi.doe.gov/Nemve1/Nemve1.info.html). These sequences were then further analyzed using HMMER (Howard Hughes Medical Institute, Chevy Chase, MD, USA) to identify protein domains [20]. For full JGI gene models, see Additional file 1: Table S1.

### RNA extraction and cDNA synthesis

To confirm the predicted sequences from *N. vectensis* as well as to generate plasmids for qPCR analysis and *in situ* hybridization (described below), we amplified, cloned, and sequenced portions of each predicted transcript (primers designed with Primer3 (Genomics at Estonian Biocentre, Estonia), primer sequences listed in Additional file 2: Table S2) from cDNA. For characterization of sex-specific expression patterns, RNA was extracted from three biological replicates of pooled samples of males and females using the Aurum Total RNA Mini Kit (Bio-Rad, Hercules, CA, USA) with on-column DNAse digestion, as described previously [21,22]. For the developmental time series, RNA was extracted from three biological replicates from four developmental stages: embryo (0.5 to 1 days post fertilization (dpf)), early planula (3 to 4 dpf), late planula (7 to 11 dpf), and juvenile polyp (8 to 23 dpf). From the total RNA, cDNA was synthesized with the Iscript cDNA Synthesis Kit (Bio-Rad, Hercules, CA, USA) using 1.5 μg of RNA per 30 μl reaction.

### Quantitative real-time RT-PCR (qPCR)

Oligonucleotide primers (Additional file 1: Table S1) were designed to amplify each *N. vectensis Dmrt* gene. Primers were 20 to 23 nt, with a GC content of 40 to 60%, in most cases, spanned a large intron, and produced amplicons with minimal predicted secondary structure (m-fold, [23]). A standard curve was constructed from serially diluted plasmids containing the amplicon of interest for each gene. The standard curve was used in qPCR reactions to quantify amplification efficiency and to calculate the number of molecules per reaction as in [21]. qPCR was performed using iQ SYBR Green Supermix (Bio-Rad, Hercules, CA, USA), and reactions were run using a MyCycler Real-Time PCR detection system (Bio-Rad).

Standards and experimental samples were run on a single plate. The PCR mixture consisted of 11.5 μl of molecular biology grade distilled water, 12.5 μl of IQ SYBR Green Supermix, 0.5 μl of 10 μM gene-specific primers, and 0.5 μl of cDNA. PCR conditions were as follows: 95°C for 3 min; 40 cycles of 95°C for 15 s, and 64°C for 45 s. After 40 cycles, the PCR products from each reaction were subjected to melt curve analysis to ensure that only a single product was amplified. The number of molecules per microliter for each gene was calculated by comparing the threshold cycle (Ct) from the sample with the standard curve.

Expression data for developmental stages was standardized by total RNA input into the cDNA synthesis reaction. Expression data for the comparison of males and females was standardized to a constitutively expressed heat shock protein, which served as a control gene. Expression was compared among developmental stages (embryo through juvenile) using one-way analysis of variance (ANOVA) with Tukey’s honestly significant difference test as a post-hoc test. Expression differences between sexes were statistically compared with a *t*-test.

### *In situ* hybridization of adult polyps

Results from qPCR indicated that a number of *Dmrt* genes were differentially expressed between male and female anemones. Expression of two of these *Dmrt* genes (E and G) was determined with *in situ* hybridization using 8 to 10 tentacle adults following standard protocols [24,25]. Probes were synthesized with the DIG RNA Labeling Kit (Roche Diagnostics Corporation, Indianapolis, IN, USA) using the cloned sequences used for the plasmid curve for qPCR analysis.

## Results and discussion

### Protein domain organization in *N. vectensis* DMRTs

**Figure 1:**
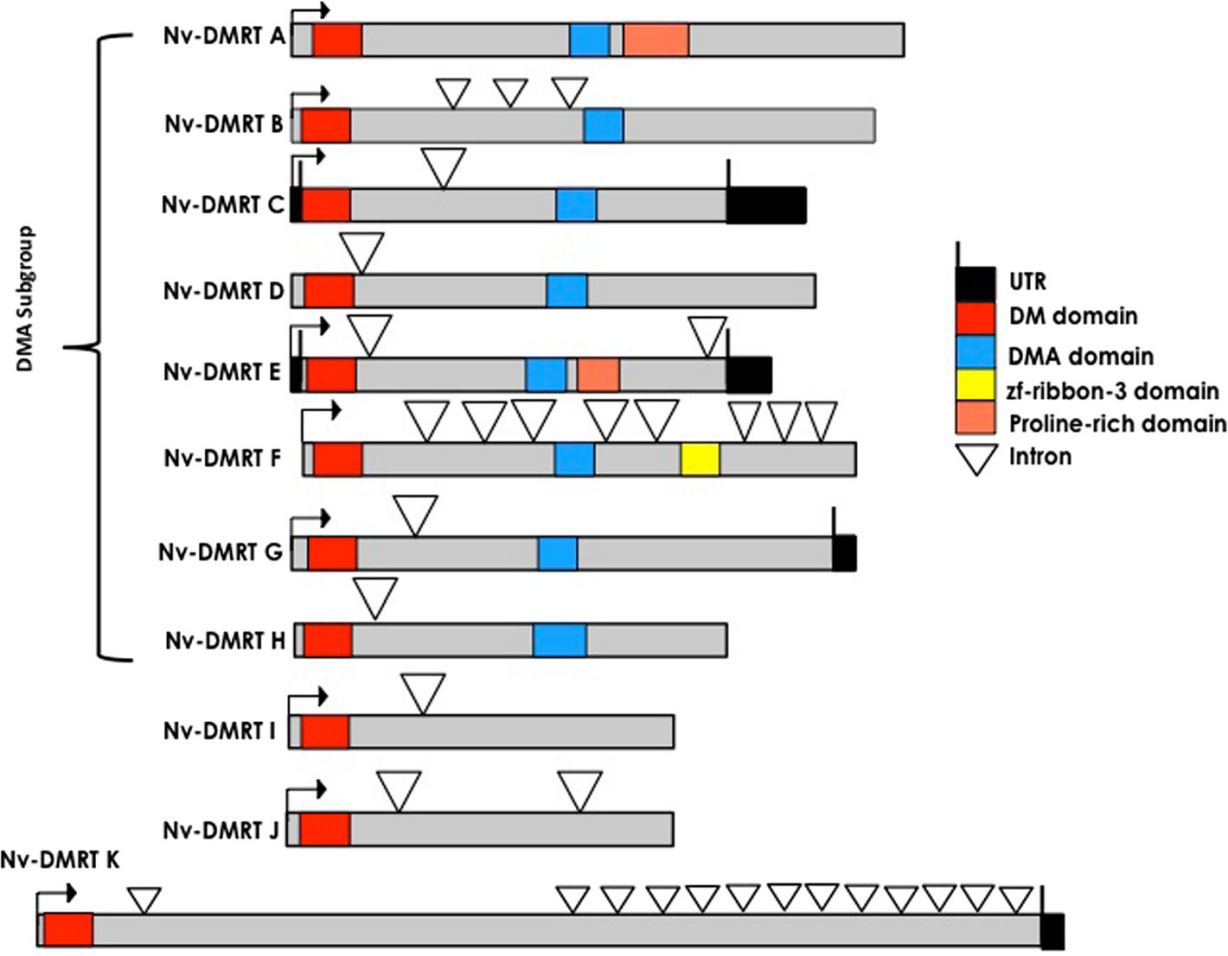
Protein/gene models of the *N. vectensis Dmrts. NvDmrt* A-H have DMA domains present. Additionally, a proline-rich domain is found in *NvDmrt* A and *NvDmrt* E, and a zf-ribbon-3 domain in *NvDmrt* F. The role of these additional domains in *N. vect*ensis is currently unknown. *NvDmrt, Nematostella vectensis* doublesex and mab-3 related transcription factor.

Comparing the 11 *N. vectensis* proteins, NvDMRT A to H all have DMA domains (Figure 1). Additionally. NvDMRT A and E have a proline-rich domain, while NvDMRT F has a zf-ribbon-3 domain present. The full coding sequence for all *N. vectensis Dmrt* genes has not yet been sequence confirmed (e.g., *NvDmrt* K) resulting in unreliable gene models; thus, additional domains may be identified when these genes are verified. These results confirm that cnidarians have DMRT proteins both with and without a DMA domain, like previously reported in protostomes and deuterostomes, further suggesting that the domain diversity in bilaterian DMRTs dates to early animal evolution.

### Differential expression of *Dmrts*: developmental stages

**Figure 2:**
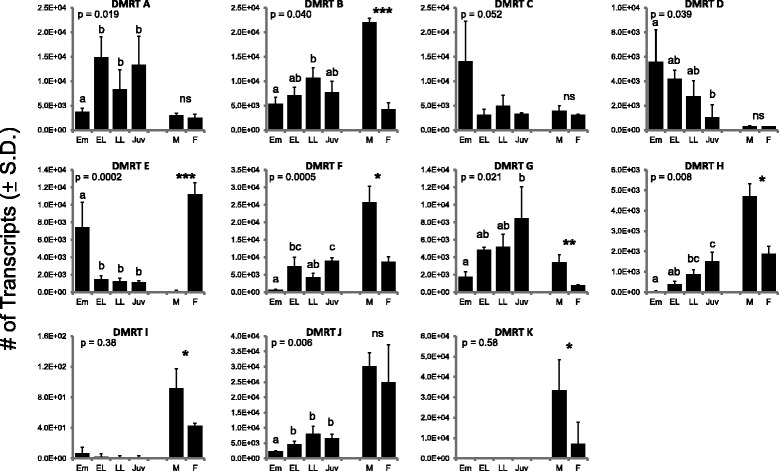
Quantitative PCR results for expression of 11 *NvDmrt* genes over a developmental series (Em = embryo, EL = early larvae, LL = late larva, Juv = Juvenile) and between males (M) and females (F). The developmental series results from statistical comparisons are shown with the *P* value, and letters above bars indicate statistical grouping based on post-hoc comparisons. Significant differences in gene expression between males are females are indicated with an asterisk (*P < 0.05; **P < 0.001; ***P < 0.0001). NS, no significant difference.

Previous research reported the developmental expression of one of the 11 *N. vectensis* Dmrt genes, *NvDmrt* B. *NvDmrt* B is expressed in a “salt-and-pepper” pattern during embryogenesis in presumptive neuronal cells, and morpholino-based knockdown of this gene resulted in a reduction in Elav-1 positive neurons [5,16]. This result is consistent with the role of *Dmrt* genes in neurogenesis at the cnidarian-bilaterian ancestor but does not address the other potential functions of the diverse suite of *Dmrt* genes in cnidarians. One component of gene function is the timing of expression during development. Our quantitative comparisons of *Dmrt* gene expression for all eleven genes over a coarse development series showed a significant difference in expression for eight out of the eleven *Dmrt* genes where different stages had peak expression (Figure 2). For example, *NvDmrt* E showed the highest expression at the pooled embryo stage and in female adults, with modest expression in the other developmental stages (Figure 2). *NvDmrt* A showed relatively high expression in all developmental stages except the pooled embryo stage and the adults (males and females). Together, these data show that *Dmrt* genes in *N. vectensis* have differential expression during development.

### Differential expression of *Dmrts*: females and males

**Figure 3:**
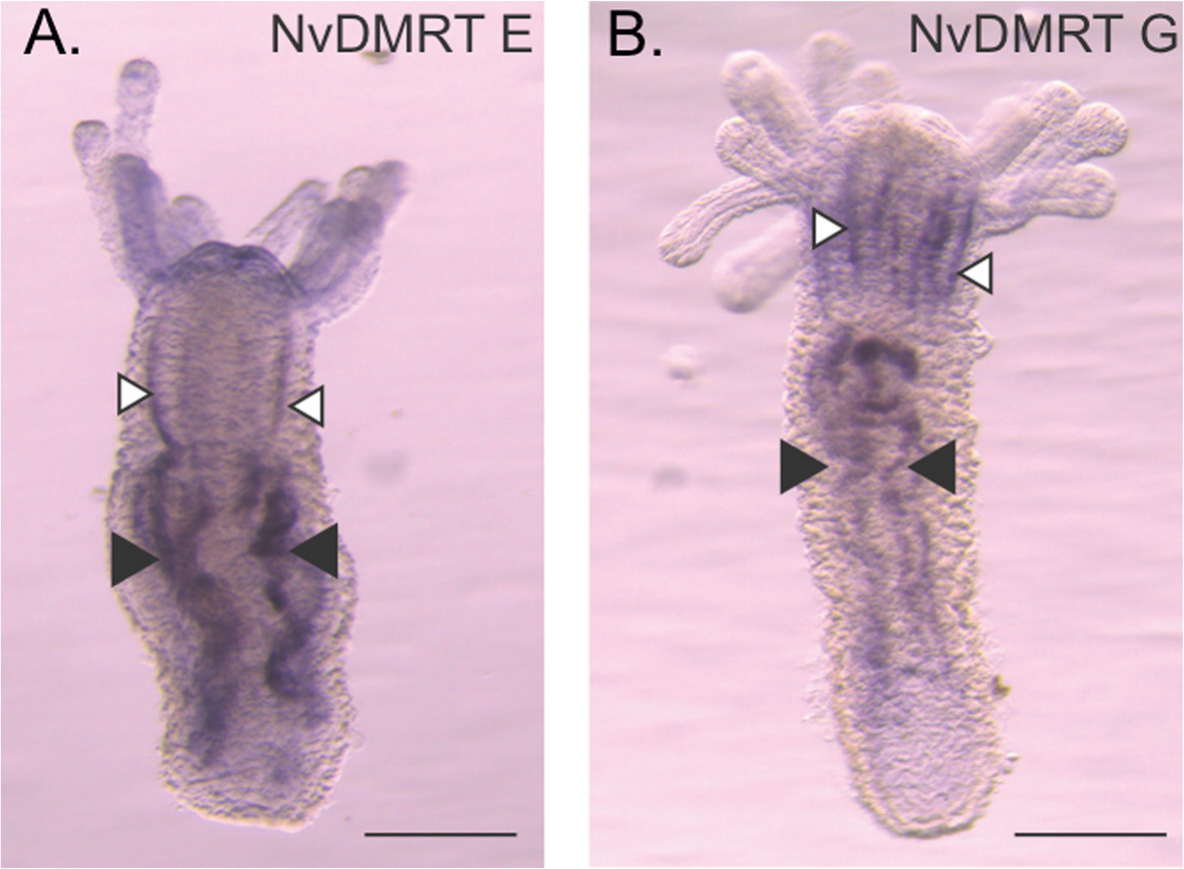
Spatial expression of two *Dmrt* genes in young adult polyps by RNA *in situ* hybridization. Staining of **(A)** *NvDmrt* E and **(B)** *NvDmrt* G are found throughout the mesenteries of the polyp. Black arrows denote the subpharyngeal mesenteries, while the white arrows denote pharyngeal region. Scale bar = 0.5 mm.

Due to the function of *Dmrts* in sex determination and differentiation in various bilaterian animals and our observation that many *Dmrt* genes have higher expression at the adult stage, we compared expression of all 11 genes in sexual mature female and male anemones. Seven of the eleven *Dmrt* gene tested had significant differences in expression between females and males, all higher in males with the exception of *NvDmrt* E (Figure 2). *NvDmrt* E had considerably higher expression (>78 fold change) in females with low expression in males. Males had higher expression in the six other *Dmrts*, with *NvDmrt* K having the highest expression (>24 fold change) (Figure 2). To investigate if two of these DMRT genes with differences in expression based on sex (E and G) were expressed in regions potentially involved in gamete development, we determined spatial expression with *in situ* hybridization using 8 to 10 tentacle adults. Expression for each gene was located throughout the subpharyngeal mesenteries and extended into the pharynx (Figure 3). Efforts to determine cell-specific expression or sex of the polyps with histology were not successful. Thus, while these data suggest that these genes are expressed in the gametogenic regions of the polyp, we cannot discern the likelihood if they are involved in a sex- or gamete-specific process. Further work comparing expression of these and other *Dmrt* genes with other gamete-specific markers (e.g., SYC1 and 3 [26] or GCS1 [27]) would be informative. Additional *in situ* hybridizations will need to be conducted on adults as well as developmental time series to better elucidate the spatial expression of these genes.

## Conclusions

Previous research has shown that the *Dmrt* gene family evolved early in the metazoan lineage, likely in the ancestor of all animals due to the presence of these genes in all phyla but not in unicellular outgroups [14]. We show here that for the cnidarian *N. vectensis*, which has the most *Dmrt* genes for any species yet characterized, the diversity of genes is accompanied by an equally diverse pattern of expression during development and in each sex. *Dmrt* genes from various species commonly have splice variants that impact the function of the translated proteins. In our studies, we did not detect any splice variants for *Nematostella Dmrts* nor were any reported in previous studies that annotated *Dmrts* from this species. Our targeted PCR and cloning approach was based on genome annotations, not high-throughput sequencing; thus, we cannot rule out the possibility of splice variants. Thus, the expression shown in this study may be due to isoform specificity and may not represent all the isoforms including those that may be critical for sex determination. Significant differences in the expression of particular *Dmrt* genes between females and males may serve as useful biomarkers for determining sex of individuals, both in the laboratory and in field collections of natural populations.

## Additional files

Additional file 1: Table S1. Gene models from the Joint Genome Institute for all *N. vectensis* DMRTs. Red indicates the coding sequences, gray indicates buffer sequence, black indicates intron regions, and blue indicates UTR regions. Additional file 2: Table S2. Primer sequences and the *N. vectensis* sequence ID used for cloning and quantitative PCR.

## Abbreviations

*Dmrt*: doublesex and mab-3 related transcription factor
Nv: *Nematostella vectensis*
