# Supplementary material for: Sex-specific and developmental expression of Dmrt genes in the starlet sea anemone, Nematostella vectensis
Source: EvoDevo. 2015 Apr 25;6:13. doi: 10.1186/s13227-015-0013-7 (PMC4433094; doi:10.1186/s13227-015-0013-7)
Supplement: Additional file 1: Table S1. — Gene models from the Joint Genome Institute for all N. vectensis DMRTs. Red indicates the coding sequences, gray indicates buffer sequence, black indicates intron regions, and blue indicates UTR regions. [file 13227_2015_13_MOESM1_ESM.pdf]

DMRT A

```
       204738 AAGACCATTAACGAGAACAGGGAAAGATCAAGAGAAGACGTTCACGTCTTGGTAGTTATTGTAAACTCTT 204669
204668 ATGTTTATCTATCTAGAGTATGATATTATCAGTTATAAACAAAAAGTGCTTGCATGAATCTTGTATTAAT 204599
204598 GACAAGGTCTTGTTTATCCATGCAGCTGAACCAAGGATGTCTGATATGGCCCTCAAAAGGCAACGTTCAG 204529
204528 AAGAGGACGACGAGCCCCCAAACAGTGACCGAAGATCGCCCGATCAAAAGCGACCACGTCCGGAAAACAT 204459
204458 TGTTCTATCAAATGGCTTTTCAAATGATGTTCATGGACGTATGTTTTCAGCGGAGCACGCACGAGCTATG 204389
204388 AATATCTTGACGAGACTTTTCCCGGAGCAAAAACTCAATGTGTTAGAGCTGATTCTCAAAGGGTGCAGTG 204319
204318 GGGACATTGTACAGACTATAGAATGCGTTCTGCCAAGTCACGAGGAAGCCTTAGCAAGGGCTCAAATGTT 204249
204248 CACTACTGGACCCCCAAGGGGAATGTTTCCCATGCCCTCCCCACTGAGGAATGGATTGTCAGCCTTCTCG 204179
204178 CCTATCACACCAGGCTTACGGCCATCCTTACCCATTATGCCTTTAGAGCTTCACACTGCAACGTCATGTG 204109
204108 CCAGTGGAAAGTGCCCTGGGTGTGTGTACTACCCAGGGATGGTCCCTAGGAGTCCCAGAGATGCCCCCAA 204039
204038 GGAGCCAAGGGAGTATGCCTCTCCCCCACCACTGCCCTCTAAGTCACCTCGGTCACCAAGAACATCAGAA 203969
203968 AGTCGTTCGCCCCCAAGGGAAGAAATGCTGGACAGTCAAAGAGTGCACTCTGCTACTGCTGCACTCATTT 203899
203898 CAATGAGCAACAGTGCACAGTTATTCCACAATGAACGCTCATCTGTCATTAGGAACAATATGAGCATGGT 203829
203828 GGGATCCCCGACAAGTGTCAAGTCACATGATTCCGTCAGGGGATCCCCATTAGGGAACTCAACAGGGGAA 203759
203758 TCTACTCCACCCAGTAATTAATCTTTGACTACTGTACAAAACATTCAACCCATTTTCACTTTTTTTCTTA 203689
203688 GTTATTTAGTGCTAGTTAGACTTAAAAG 203661
```

DMRT K

```
765630 CACGCCCCACGCTCAAAATTGATTCACGCACGAGGTAGCCAATCAGAACACAGGACAGCAATGATTGACA 765699
765700 AGCTAGAACAGGAGAGGCGCGCAGTTCCGATCATTCCTTCGCAAGATTTGCCAAACAGCACGTTTTGGAA 765769
765770 CAACGTTGTTCAAGAATTTCAAAAGACTGAAAAAGAGGTTCTTGCGTGTTAATCTACATTATGGAACACC 765839
765840 TTCCAAGACGTTCAAGAAACCCTAGTTGTACTCTTTGCAGTAATCATGGTATCAGATCGGAGCTAAAAGG 765909
765910 ACATAAATACAATTGTCCTTACAGTTCGTGCAAGTGTGAGCTCTGCGACCGTGGCAGAGAAAGACGGGAA 765979
765980 ATCATGCGAAAACAAGTGAAATTACGGCGACATCAGATGAGAGATATTTTATCAGGGAGAGAAACCGCGG 766049
766050 CTCCCGTTGAAAAATCGATTCAGGAAAACTTACAGGATCCTAAAGTATGCAATCAAAATGGTGAGTGAGT 766119
766120 TTTGATTGTTCCTTCTCATTCATCATCGTTGTTACATGCGATACATGTGGTTGTTTGAAAATTGACTTTG 766189
766190 AACTACAACTAAACTTAATAATTCGATTTATCTGAATTTTTTAAGGCATCACTTAGTATAGAGATTGATG 766259
766260 TAACTCTTGCTTAACGAAGCATTTTTTGACGAACCAAAAAGTGAATTTCGCTTCGCTTTGCCCGGCACAA 766329
766330 TCACAACTTGTCTTTTCTTTTTACAAAACAAATCAAAGCCTGGCGCCTTTTTATTTGCCTCATGTCTGAT 766399
766400 TAACAAATATCCTTGTATATTTGTGTGACTTTATATTAAAAATTGAACCTAAAAAAACAATTCACACAGC 766469
766470 CTTGTAAAATCAATGTTTCTAAAAAGTGGCGCACACTGGCGACATAACGGTCCATGTAACACTATGCCAT 766539
766540 TCGATGTTTTTGTAGTTTTTTTTTTAAGTAAAACAATAAAATTCGAATGTGATTTTCGTTCTAAATTTTC 766609
766610 GTTTTCGAAATCCAATCCAAGCCCTCGTTTTTAATTTTGTGGTTTCATTTTTGTGTTTTAAAATCGAACA 766679
766680 TTAAAAAGGAGTACCTGTGTAACACTCCGCCATTCAATAGTTTGGCGCTTTTCAGAATAAAGTTAAAACA 766749
766750 AGAGAATTGATTATTCTTGTTAATTTATAGGTTCCGTTAGAAATTAAAAACCAGCCAGTCTTTTTTTCCC 766819
766820 TTGTGTTTTAAAGTAATAATAGATACCATTATACGATCTTTTTTTCTCTCTTTAAAATGAATAATGATGA 766889
766890 TGTTTAATGATAAATGAATTATGATTTAACTAATTTAAATATCCGTTTTTCTATTTTCATATCCATCCAA 766959
766960 TCTAACGAATGTAGGCTTCGGAGGGGGAGGGGGGAGGGCTGCCCCTAAAATGGATGCCATACTAACTAAG 767029
767030 GGAGCGATGTTTTGGCAGTATAGTATCAAGATGAATTGATCTGAGGGAGGGAAGTGCTTGAGTTAGTCTC 767099
767100 CCCCTGATAAATATATTTTTGACAAAATTAGTAATAGTCCCTCCCCTTTCTTCAAATGTACAACCTTTGA 767169
767170 CGGGTGTCCATATGAGCAAATGAGCTGAAAATAAAACACGAATATTTTACAAGTATGGAAATGAGTTTAT 767239
767240 TTCGATTGAGATTAATTCCCAGGACAAAAATCAGATAATGATACTTGTTATTTATTTTTCGATTTTTAAA 767309
767310 GCCAAAAAATAGAAGTGTGAAGCCTTGAAATTTGATTTCTAAAATACATATTTTAATTAAAAATACATGT 767379
767380 TTTTTTGTTTATGTTACTATAAAAAAACATAAAAATATTCTATGGCAGAGTTTTACTCAGACTTGATTTT 767449
767450 TTATTTGATTTTCGATATTAAAACGCAAAATAAAAAATAAAAAATTAAAAAAAAGAGGGGCTGAATTTGA 767519
767520 TTTTTGATTTCTATAAGGGAAGTTTAAAATAAAAATCACGTTCAAATGTTTTTTCCCTTAAAAATACCAA 767589
767590 ATTATTAAATGGCGGAGTGTTACACGGACCCATAACGACCCAGGCGTGCATTGTTTTATGTTCTTATGAC 767659
767660 AGGGATATAACATGATATAATAAAAGAGGATTATTTTGGATTGTTTTCCTTTTTGGTAGTTGAAACAAGT 767729
767730 ATTTCTGATTGCTGACATTGCTCAAATAAGCGCCTCGTCTAAGAGGAGAAAACTGATTAAGTATGATGTT 767799
767800 TCACCATCTGTAGTTAGAGGGTGGCAAAAAATTACTTCTTTATCAAGTGCTGTAGCAGGCTTCAAAATAT 767869
767870 TGGGGGAGCAAAATAAAAACCTTTAAGAAGATATTTATGGGCACAGCCATACTGGCCATTTTCTTGTATC 767939
767940 TTTTAAAAATATTGAGGGGGGGAACACGTGATATGGCCCTGTTACCAAATTTACTATTATCATTTCCTAG 768009
768010 GCTTCCAGAAGGAACCTGTAATTCCACCCATAGGTATGAGTCATCCAATTATTGGACTTCCTGAAAGTCC 768079
768080 TCTCCTGAATACAAAAGTATTCTACCCTCGGTTCGCACACCCAAGCTCCCCAACACTACGCCACAATGCT 768149
768150 CCGCCAGTACCAATGGTCTCATACTCACCCAACAGCAAATACTACTGTCACTACTCCCCATATCTGTCTC 768219
768220 TCCGTGGCCTTCATGCTGAGCCGCAGCACCACTCTAGCCCACAAGGATACCAAATCAGCAAACCGGTACA 768289
768290 GATTGGAGGCTTCAACACTTCAGAGAATAGCGCCTTTGAGAAGTGTGCAGGGGTGGTCTTTACTAACTCG 768359
768360 AGCAGCATGCTTGCTACAGAGGCAGCAAGTGCCTTGGCTACTCTGGCACAGTCCTAGGAAACATATGATT 768429
768430 GGCTAGTTTAGCACAGTCATAGGAGACATATGATTGGCTACTCTGGCACAGTCCTTGGAAACATATGATT 768499
768500 GGCTACTCTGGCACAGTCCTTGGAAACATATGATTGGCTAGTTTAGCACAGTCATAGGAAACATATGATT 768569
768570 GGCTACTCTGACATAGTCATAGGAAACAAATGATTGGCTACTCCGGCACAGTCATAGGAAACAAATGATT 768639
768640 GTTAGTGCCTTGGCTACTTTAGCACAGTTATAGGAAACAAATGATTGTTAATGGGTTCACTACTCTAGCA 768709
768710 AAGTCATAGGAAACAAAGAGTTTTCCCATATAATATCTATCACTGCTCCCTCCCTCCCCCCCCCAAGCAA 768779
768780 TTCCTGGCTAATCGCCTAGCTAAATCTTTAGCATTCCTTGCACTGAGAGGGTCTCCGTGTCGGCCACCAT 768849
768850 TTTGTCTTCTCCCAGCTTTTTTGAGACTATCTTTGCGTAATTTGTCTTGAAAAAACTGAATGAAAACAAT 768919
768920 AACAAAATTGTGGCAGACAACATTAAAGGCCCTTTAAAGCTTGCAGCTATCCCCCATACTCCTTTGCATG 768989
768990 TCTTTTCTGTCTTCCCATTATGCGAGTTGAGCCACTGGTGAGGTTAAAGTCAGGCTATAAGAAACAACTG 769059
769060 ATTGTAAGAGAATACAGGAGTAAAGTGTATGGGACAATCAGCAAGATTTGTGTTGTACATTTTCAGATTT 769129
769130 CATAAGATCAAGTATAACTAATAATTAATTTAATTGTAATTATGTGTGTGAACTGTTAATGTTACAGTTG 769199
769200 GTCCTAGTTAGACCTATTTTTTGTTGAATTAATCTGATCATTTTTGCATAATAAGGAATAAAAAAGAAAA 769269
```

769270 AGGAAATAGAGTTTTATTTTCTTTAGAAACTGGTAACAATAAAAGTGATTGTGGAATACAATAGGGGGGT 769339
769340 TCTGAGATGGTCATAAATAAATAGATCAGAAATAAAACACAAAAATGTCACTTGTAACTGTTTCATGGAA 769409
769410 TTATCTTACTACCCTAATCATTTTTTTTTTACCATTATACTTCTCTAGAGTGTAAGTAAAGTAGTAGAA 769479
769480 AAGATAGAATAACTACAATTCCTTGGCATTATTCATTGGCTAAGGCTCGTTCCAATTTTACCCATTTTTG 769549
769550 GGCACCCTGTGGAAACCTAAGACTTTCCCGCCATTTGTCAGGAGCGCGCGGCGCGATGGCTGACATGTTT 769619
769620 TTTGTTTGTTTTTTCCTTTGTTCCAGCTTTCAGATTTGTTTGTTGATTTATCTGCCAGTCTTATTAGAGA 769689
769690 AGCTATCAGATAATTAGCATTGAAAAATTCCACTCCTGTATCGTAGCGTTTTTTCCACTTAGACTTTGAA 769759
769760 AGTCGTGAATTAATCATGAGTTCGTTCACGGGGTTGTTTTTCGGTTCGTTACCTAAACTCGGCGAGTTGA 769829
769830 TGGCGGTGTTGATGGAAGAAGTGAAGGAAAGTGATGTCGCAGCTATACAGCCAGGATGTTGCAGGTATTC 769899
769900 TATGCGCATTATTGCTAAGGATAACCTTTAGGTATTAGAGCAAGACTATGCAAGATCTGAGTACTGTATA 769969
769970 CAAATAACATGATGACATGATAACGGAGCGAAACACAAGGAACGCTCTTTTCATCAATCGATAGCATGTC 770039
770040 ATTCATTAACTAGATAATAAAGATTAATTTTATCCTGAAAGTGTTTTCCATCGTGTATTTGTTTGATAAT 770109
770110 ATTACTGGATAAAAGAGTGAAGTTGAATTTATGCTTTTCCAGGGAGTTAATATTTACAGAGTCCAGAGTG 770179
770180 ATACTGTCCCCATGTGTTGAAAACCCAAAGCAAGTATTTCTTATTTGCAAGGTAATAAGCTTACTACCCC 770249
770250 TTAATTTTCTTTTGTTCCAATGACCCCTTTATCTTACGGAATTCAAATTTTTCAACAATTTTGTTAAGG 770319
770320 TTGATATTCCCAACATGACCAAAAAACCTGGAAAATTCGCAATTCGAGAGAAAAAAGACACAATTTCCTA 770389
770390 CAATGGCCATCGATTCCTTGTATTCCGGACTGATTTTTAAACACAAATAACGTGAGGCCGAAATCCACA 770459
770460 TACCCAGATTTTTGCTTTTCAAGATACTTTGATCATCTTTGCGCTATGTATGAGCTCAGGGACGAAAAGC 770529
770530 TCAAATTTCAATGTCACTTTACAAAAAGTTGCATTTGATATTTTGTTTGCTAGGACTCACTAAGTACTTA 770599
770600 GAGAAATTTTCCGCTTTATTAGATCTGAAATGAAGTGTTAAGTCATGCCGTCATGTCGCTTGAACCCCCT 770669
770670 CCTACACACTATTTGTGTAACCCCCATCCTGACCACCAAAAATATTCCCTTTTGAACAATTAGCAAAATC 770739
770740 TTTGACTTTTCACATATATAATGGCTGTCACCATCAACCTTCAGCAAACCCTAGCAACGTGTCTGTTTTA 770809
770810 TTTCCATCAAAAAATGATAATGGTCCCAGTAAAGATTGTTATCAATTTATAAAAGATATTTACCAGCCTC 770879
770880 CTGATTAATAGTTCTTTTTATACAGAGGGAAGTATGGGAATGTTCAAAGCTCCAAGCTAATCTGAACAAA 770949
770950 CTAAATCTTGAGGTAATTATTAAGTTGTGATGATATTTTTAGTTTGTAGAGGCTGGGGTCTATGAGGGGC 771019
771020 AGCGGGAGGGAGAAGGTTTGGGTTCGCCCTTCCCCACTCTGCCACCGCCATCTGCCTCTGCTATCCAACC 771089
771090 AATATTAGTGATAATTCAGTTCTAGTACTGAGTACTGGTTGCAGGAAATAGCCAGGGCTTTCATTAACTT 771159
771160 TCAGAGTAGGTGGTGGAGATTGATAAGAAGGGGATGGAAGTTATTTGATCTTTTTAGTTCAATTAAATCT 771229
771230 TAAGTCAACTTTTAAAAAAAAAGTATCCGGTGCTCAGTGGAAAATAGCTTGCGCTTCTGCCAGCCGCCGG 771299
771300 GGCCTAATGAAACCCCTGATAGCCTATATTGAGTCCGATCATAAGTCATATGTCTCATATGTATTTCCCT 771369
771370 TAGGTTGTTGAGGAGGTAGACATCAGTAAGAGTTTGTTCCAGTTCTGCCTTTTCCTAATAATTCAGCTCT 771439
771440 AGTACTGAGTACTGGTTGCGGGGAATAGCCTAATTGAGTCGACGATCATAAGTCATAATGTCTCATATGT 771509
771510 ATTCCCCTTAGTTTGTTGAAGAGGTAGACATCAGTAAGAGTTTGTTCCAGTTCTGCCTGTCATTCACACT 771579
771580 CACACAAAGACTGGCTCCTCTATGGAACAAGGCCGGGGACTATTTTATTCAAGGTATATATGTACCCTGA 771649
771650 CCAAAGGCTAGTGAACAATTCACATAATCCACATTAGAACACTGAAAAGCAGGTCATTATTGACAAAACA 771719
771720 AAAGATCTTTTGGAAGGCAATATGCAAAAACCTTTATATGAAAGGTCGGCTCTTGTGCTTTGCATTTGTG 771789
771790 ACCATACCTCACACAGACCTCCCTCTAAGAACCTGTCTACATTTGGCTATCAGTTACTTAGATGGTTGGC 771859
771860 TTTGCATACAGACCCATCTAAGAGCCAAACTACCCTTGCCTTTCACTAGATTTTTCATTTTTTTCTGTTTG 771929
771930 CATTTTTAATTTTTTTTTAAAATAGTTTTTGAGGTTTTGAAAACCCTCCAATATCCAACACCCTCTCTCA 771999
772000 TGGGCGGGGGGGGGGGGGGGTTAGCGGATTTGTCCCCGAAATGCTCACGGATTACGGAATTCGATGATT 772069
772070 ATTTCAGCGGTTTGGCAGATTTGGGAAATATGGCGGATCACGGATCTGTCTACAATTCTGGCGCGGATTT 772139
772140 CTGATTTTGGATGCTTTAATAGTCCAATTTCGGGTATTAAATGAATTTCAATCGATGCTATTGTTTATCT 772209
772210 GTCATACCATGCGGATCTAAAAATATCTGCGGATCCACGGATTTGCCCCGAAAATGTAGCGGATCGGAGG 772279
772280 ATTTACATACCCCTATTCACCCCCCCCCCCCCTCATGAACCATGCCTGAGCTAAGTCACATCTTCATCTC 772349
772350 AAACTTTAACTATATTTACATTTTTAATGTATTTAATTATCACATAGGGCGAGATTTCCTGGAAACATCC 772419
772420 ACAAAGCAGAATGCTGTCAGTGAGTAACTTAGAAACTGTCTGATATAAGGGAACTAATCTTCTTGCTCCT 772489
772490 CTCATTAGAGAGACTGTCGTGGACTAGAATGACTACACTCTAACGCGATCTAGTACACAACAGAATTTAT 772559
772560 CACTCTTTCCATGAAGGCGTTACACAATTCTGTTATCCAGTCTTGGATTCTCTCTAGATAACATAGCTGT 772629
772630 CGTGCTGTTTCACAGCTTATCTTAACGCTTAGCACAACCCTTAGCTTAGCCACTAAGCTCACCACTCAGC 772699
772700 TCCCTAATTCACGAGCAAACTCCTTATATACTCTTGGGTGTCTCTAAATATACCTTATTACATCATAAAT 772769
772770 GTTGTTACAAATAAAAGAAACATTACATCATCCATTTTGTTATCATAAACTGCTGAGTCAGGGTTTTAGA 772839
772840 ATATTCTAGCAAACAGAACAACGATCCTAAGTAAAAAAAATCCTATTCTAGCGTGTTCTAATAGCATGTA 772909
772910 TTAACTTCTACAATGTTTCAAGTAAATGTTCTAGACATTACTCTGTCACAGAGACTCTGCCGCCATTTTT 772979
772980 GTCATTCTAGCAAAATGCCAAGTGTGAGAAAGCATCCAATTCCATCGGCTTGATTTTGTTGAAAGAAGTG 773049
773050 TAAGAACAAAGGTGTGACTGAGAATGGATCACTAAGCCCTGTTCTGTTTAAGTCCTAGATGTAAGCATAA 773119
773120 CTGACAAAGTCTGCATTGGACTTGAAGCTGTAAATCTTAAAATGCAGCCTTCTAAAGTAAGAATCATTGT 773189
773190 AATTACCTATTTGAAATAGTTCTTTATGATCATAGCAAGTTTGAAAATCAATGTGTCGTATCATGCAATG 773259
773260 GGTATCCAAAGATATAAGTAATAGCAGTCGCATTACCAGGATTGGCCGAGTTAGGAGGGGGGGGGGGCAG 773329
773330 TCATAGGTATCATACAGTAAGAAAAACGCATAGTATCTGAAGATTCCTTAATAAGGAATGACTTATTTTT 773399
773400 TGGCCGCACCTCCCTTGTACGCTCCTGAAAAGCATGCAGGTTTGGAACATTGTAGAATTCTTCATGCTAA 773469
773470 AAAAACATGGGTTCATCCATTTATATGCTACACCTGAAGCACTGTAGGTTGCGACACATGAATTCCCAAG 773539
773540 TGCGACCTTATTTGAAGAATGTATAAACTAGCCAAAGTTATGTACAGGGGCACGGTAAGAGTACAACAGG 773609
773610 TGATGGTGAGGTTGAAACCTCCATAGTTGTTATTTTATGTTTGTTTTTTATGTACAACTACAGCTACAAG 773679
773680 CTGCTATACAAATTGCTATATTATATATAATTTATAATATATGCTCACCAGGTGAATCAGTACTGTGTGA 773749
773750 ACTCCAAGCTACTGCAGAGTTTTAGCTGCTGTTTTGATGCCAGTATTGGTGACTGTGCTATCCAAGATGA 773819
773820 TTTGTGTTATGTGTTACCAAGGTATTAAAGCAAGTGACCATATATATCATCAGGGGCATTAAATGTGGGA 773889
773890 ATCCTCCGTTGTACCTCACTTAGGGGTGGTAGTGGTGGAGATTTTCATTGGAGAAAAGCTCCTGTTTTGC 773959
773960 AAAGTCCAGTATTCAATGTCTGTTACCTGTTTGTTCAGGCACCTTTTAAAGAGTTTTATGAAATTTGTCA 774029
774030 TGAAATCTTCATTTAACGAAATAAGATCTGGAATCTGTAGTTTAAATATTTCAACCTTTAACAGCCTGAA 774099
774100 GAAAGGACGAGTATTGAGTATTACCCACCAGCTTCCAGCAGACTCACCTTTTGCAAGCTACGAGGACATC 774169
774170 AGGAAATATTGGAAACTGGTGGTAAGCTCTAGGTGGTTATGTCAACTCTCACACTGCAAGTCTCTTAGTG 774239
774240 ATGGGGTTTTGGAAACAGGAATATAATTTATGAATTTTGACTTGGAGGCTCTGAAGCTCCAATAATTGAA 774309
774310 AATTACTGCAAAATTAATCGTTTCCATACATTTACTGCAACTCAAAGCATTTATTTTTGTAGGTAGCTGC 774379

774380 CCCATCTTTGCCACTGCCTGAAATAAAGATGTGTGGTGTTGCTAAAACATGTTTGTATATTTTCAGTATG 774449
774450 GTTACCGGTTGCCTGCTCATGAGAAGGTATTCTACAATGTGCGGTTTTTCTTCAGGAAGTCTAAAGTTTT 774519
774520 CACGTATCCTACATTTCCATCTGTTTGTAAGGAAATTTTTTTTTACTGTAATATCGTGATGTGCTCGTAC 774589
774590 TGCTACTCCTGGCAAAGCCTCACTTGGTTGAACTAAGAACCTCTTAACATGAACCATGTGGAACAGCACC 774659
774660 TCTCAATATTTTGTGCAATCAGAGGCAGAGAGCTTCTTAGGTTATAGGTACTTTGTCAGCCTCCATTTTG 774729
774730 TCACCCTAGAAAAATGTCACAAAATGTTGATGATAAGCACCACTTTCATAGGCTGATTTGGGGAAGAAAT 774799
774800 TGCATAGATTTTGAATTTAGTAAACGAATCATAAAACTTTGTTTTCAGATGGTTATTTTGAAATTTTTAC 774869
774870 TCACAGTTTGTCTTTAAATATTGCTATAAAGGGTGTAGCTAACTAGGCCTTTTAGGGGTCTGCACAAAAA 774939
774940 CAGAACAACATTGATTGATTTTTGTCTTTATGTTTTTGTGAAGGGAGGTTAAGCGGCTATCTTAGAAAAC 775009
775010 CAAAGAAAACCCAGGATCAAAACAAGTGTTCCCACCAGCATTTACCCCAAGGCATTAAATATACAGTATC 775079
775080 ACAACCTTGACATATGCCCAGTTACCCAGCCAGTTGCATCCATAGTGCAGCTCCCCATGTCTATCCCCGT 775149
775150 TGTGACCAGCATGCTATCTTCACAGCCTTCATTAACAATCTTCAAAGCCGTGTGCCAAGTGTGTGTGGCA 775219
775220 CACCCTTCAGATTGACCTCAGCTCCTCTGTACTACACCAACCGCTTACACAGTGCTAGCCAGCCGCAGAG 775289
775290 TAACCTTACACGCACTGAACCCGTACTGAAGCAATGTAGACCTTTCATGTCTACAATACAATGCAACTCA 775359
775360 AAAAATGGTGCCACACGGATAGATAATTCACTGACTCACAATAGCACCATTGCATCAAAACCTGAGCATG 775429
775430 CAGTTCAAAGCCATTATCAGCACTCGCTTCCACAAGGCCATTCAAGGAGCGGTACTTCCAGATCAGGCAG 775499
775500 TGATGCTGCTGCTTATGCACCTTATATGAAGGCTGGGACAGTACAAGCCCAATCACCAGTTGATAATGTT 775569
775570 ACTAGAGTATCCTCCGAGAAATTGTCTTCTACCAACCTGACAGATACGGGAAGCAATCATGATTGTGCCC 775639
775640 TTGTTGAACCGAAGCCTCGGATTGTTCCCCATTTTACAAACAATAAAGTGCATAAAAAGAAGAATTCTTT 775709
775710 TCAAGAGAATCAAAAAGCAGCTCTGTCTTATCATGACAGACCAAAGGTGGTACCCAAATTTAACCAGAAA 775779
775780 CTGGCCCAGAAGCTACCACAGCAGAGCTGCAACATTTACAGACGACAAAGTGAAAGGAATCCTGCAGAGA 775849
775850 AAGATAAACGCCCACCTAGTGACACTAATTCAGTTGCGAGAAATACATCCTTCTCACATGACTATGACAA 775919
775920 CTATGCTATTCAAGACAAGCTTCTTACTCATGAACAGCCACACCCAGAACAGATTAGACAGAAAGTAACA 775989
775990 ATATATCCATCGCCACATGGTAAAATGAGATCCCCCCTCACCTCTGGGAAAACAATGCTACCACAGGCCA 776059
776060 TGAACCAAGCTAATAGGCCAGTTAGTCAAATGGAGGGCCAGGAAGGAAGACCAGTGTTACCACCGGCCAT 776129
776130 TAACCAGGCCAAGAGCCCAGTTAGTCAGATGGAGGGCCAGGAAGGAATGCCGGGAAGACCAGTGTTACCA 776199
776200 CCGGCCATTAACCAGGCCAAGAGGCAATTTAGTCAGATAGGGGGTTTCACTCAAAATATGTTTTTACCAG 776269
776270 TTGTTCAAACAGCCACCAGTCACCTGGGACAACAAAGAAGTGATCCTTTCCGTGACGCAAGTGATATTCT 776339
776340 TTCCCTGAGTTCCTCATATGGGAATGCTGGGAGTAGTGCTAAACCACTCAAACGATCTCAGGTAAAGTAA 776409
776410 AGGACTGCACAACAAGAGGGCTTGTAAGAAAAGTGTAGGGATCTTGGGAAACTGTCAATGACAAAATAAT 776479
776480 GGTTGGAGGGTGTGGATAAAAAATGTTAAAAAAATGTTAAAAATGTTAAGGTTAGATATATAGGGGGCTG 776549
776550 TCTGGGACTTAATGGCTTTATGGGTGGAGGTAGCCAACTTCAGTTTATGAATCTTTTTCTACAGTCCAAT 776619
776620 GAAGCTGATAATCAACCAAAGAAAGCACGACCTAAGTCAAAAGTGCAAGAGAATGTAGATGTTCGCCAAA 776689
776690 TGGCTTTAACAAATCAGGTACGCACACAATACTTATAGTGGTAGGACTGTTTATAGTTAACATCAGCCAT 776759
776760 AACTTAGACAACTGTGTACACATTGTGAACAACATAGTTGATTGACACATGACACCACACCTTATTGATT 776829
776830 GATTGACACATGACACCACACCATATTGGCTGATTGACACATAACACCACACCCTATTGATTGATTGACA 776899
776900 CATGACACCACACCCTTTTGTTTTTTTGAAACATGACACCACAACCTATTGATTCATTGACACATGACAC 776969
776970 CACACCATATTGACTGATTGACACATGACACCACACCATATTGATTGATTGACACACGACACCACACCAT 777039
777040 ATTGACTGTTGCACACATGTCACCACACCCTATTGATTGATTGACACATGACACCACACCATATTGATTT 777109
777110 ATTGACACATAACACCACACCATATTTGATTTATTGACACATGACACCACACCCTATTGATTTATTTACA 777179
777180 CATGACACCACACCCTATTGATTGATTGACACATGACACCACACCATATTGATTTATTGACACATGACAT 777249
777250 TTCACCCTATTAATTTGAATGACACATCACCACACCCTATTGATTGATTGACACATGACACCACACCCTA 777319
777320 TTGATTTATTGACACATAACACCACACCATATTTGATTTATTGACACATGACACCACACCCTATTGATTT 777389
777390 ATTTACACATGACACCACACCCTATTGATTGATTGACACATGACACCACACCATATTGATTTATTGACAC 777459
777460 ATGACACCACACCATATTGATTGATTGACACATCACCACACCCTATTGATTGATTGACACATGACACCAC 777529
777530 ACCCTATTGATTGATTGACACATGACACCTCAACCTATTGATTGATTGACACATAACACCACACCCTATT 777599
777600 GATTGATTGACACATCACCTTACCCTATTGATTGATTGACACATGACACCACACCTATTGATTGATTGAC 777669
777670 ACATGACACCACACCCTATTGATTGATTGACACATGACACCACAACCTATTGATTGATTGACACATGACA 777739
777740 CCACACCCTATTGATTGATTGACACATGACCCCACACCATATTTGATTTATTGACACATGACACCACACC 777809
777810 ATATTGATTTGAATGACACATCACCACACCATATTGATTTATTGAAACATGACACCACACCTATTGATTG 777879
777880 ATTGACACATGACACCACACCCTATTGATTGATTGATACATGACACCACACCCTATTTTTTTATTGACAC 777949
777950 ATGACACCACACCATATGTATTTATTGACACATAACACCACACCCTATTGATTGATTGACACATACACCA 778019
778020 GACCCTATTGATTTATTGACACATGACACCACACCATTTTGATTGATTGACACATGACACCACACCATAT 778089
778090 TGATTTATTGATACATGACACCATACCCTATTGATTGAATGACACATGACACCACACCATATTGATTGAT 778159
778160 TGACACAGGACACCTCAACCTATTGATTGATTGACACATAACACCACACCCTATTGATTGATTGACACGA 778229
778230 CACCTTACCCTATTTTTTGAATGACACATGACACCACACCCTATTGATTGATTGACACATGACACCACAC 778299
778300 CATATTGATTGATTGACACATGACACCATACCCTATTGATTGATTGACACATGACACCACACCATATTGA 778369
778370 TTGATTGACACAGGACACCTCACCCTATTGATTGATTGACACATGACACCACACCATATTGATTTGAATG 778439
778440 ACACGACACCACACCCTATTTTTTATTGAAACATGACACCACACCCTATTGATTGATTGACACATGACAC 778509
778510 CACACCATATTGATTGATTGATACATGACACCATACCCTATTGATTGATTGACACATGACACCACACCAT 778579
778580 ATTGATTGATTGACACATGACACCACACCCTATTGATTGATTGACACATGACACCACACCATATTGATTT 778649
778650 GAATGACACATCACCACACCATATTGATTTATTGAAACATGACACCACACTCTATTTATTGATTGACATA 778719
778720 TGACACCACACCCTATTGATTTATTGAAACATGACACCACACCCTATTTTTTTTATTGACACATGACATC 778789
778790 ACACCCTATGTATTTATTGACACATAACACCACACCCTATTGATTGATTCACACATGACACCACACCCTA 778859
778860 TTGATTGATTGACACATGTCTACACAACCCTACCCTACTACCTACACAACCCTGGCCGACATGTGATGTC 778929
778930 TTTCCCTACTGTACTTATGGATTAACTCTTTCTGTTGATCGATTGACTGTTTCATAAATTCAGACTTCAT 778999
779000 GTTAAACATTGAATAATAACTATCACAAGTTGAGTGACTGTTTGTTTTGTTCCTAGCTTCACAAAGTCAA 779069
779070 CAGTGTGACCCTGATCGACTGGCTGAGAAAGCATCATATTGCTGTAAAGTCTAAAGACAAGAAAGCCGAG 779139
779140 CTGGTGGAGAGAGTGATGAGATATATGGCACTGCACCAGCATGAAGTTTAACAGGTAGAGACATACTGCT 779209
779210 TAGGAGCAAAATATACGTTGGCCCATAGGCTTACTGGTCCCTTCCCTTTACATGGGATATATAACGCTAC 779279
779280 ACTATCATGAAGTTTAACAGGTATAGGCATGCCAGTTAGGGGCAGGAGATTTGTAGGCCCATAGGATTTT 779349

DMRT B

236071 GAACCACGGGTTTGATTCCATTCTCAAGGGACACAAGGGGTTCTGTAGGTGGCGGGACTGTATGTGTCCA 236140
236141 AAGTGCATGTTGATAGCGGAGAGACAGAGAGTTCTCGCGGCCCAGGTCGCTCTACGAAGACAGCAAATGC 236210
236211 AGGAGCAGAGGCAGCCCTTACCGCCTGGAGATGTCTATACAGCCCTGCATAGAGTGAACGATGACGACCC 236280
236281 TCAGTCTCCGCAACCCGATTCTCCCTCCGCTGTACCCGAAGAACCCGAGAAAACAGGTACACACAACTCC 236350
236351 TAGCGCTATTACAAACTCATTGAAAAACTATCGCTAGAAATTACCGCAAACTTTGTCTATGTTTATGTTT 236420
236421 AGTTTTGACTTTTGTAATGAAGAGAAAAAGATGTTTTAAGTTCTAATGATGTTTAACCGTAATGTATCTG 236490
236491 TGTGTTGGTGTGGGTTCCAATTTCGTTTTATTTAAAGATGAACGTTTAACCGCCACCTGGAAAACAACCC 236560
236561 TTCATCTTGACCATCTTTGTTCATTTTGTCGATACAAACACATTTTGGGTTAATTTGAGCAAATAACACC 236630
236631 CTTTGCTAAGCCAAACTTGCGGTTTTAAATGTAATTTAGACAACAATATTGACCAAAGAGCCCTCGAACG 236700
236701 AAACGCGAGCAATTCTGGGAGCAATTTCGCGATCTTTGATCTGTTGTCAAATACCGTATGATTTAGAGGC 236770
236771 GATTGATGGCCTCACAATCTAATTTGAACTTCGATACAACTTCGACTTTGAAACACCACTTTTTTATTAT 236840
236841 CAAAGCCAAAGAATCGAGATGATCAAAATGAATGTTTTGATTATTAACATTTCTATAGATTTACACATTG 236910
236911 CGCGAGTCTAGCGATACGGAAAATAACTTGTTTTGTATAACCATTAAAAATTTCTGCTTGGTCGTTAGCG 236980
236981 GCCCACTCCACGCGTGCCCTTTTTCGCATTTAAACATTCAACACAAAGTTTAACAAAAGTCACCAAGGCA 237050
237051 ACCCCATGCATACTTTCCACACTTTTGTTTGTCCCAACTGAAAATTTCATTCTCATATTTCTTAACTTCT 237120
237121 TTCTTTTTCAGAAATTAAGAAGGAGGCGGAATCGCCCGTGGATGAGTCTTTCAACGAACCTCAAGACGCT 237190
237191 AATAGTTCGCATATGCAGTTCACGACTTTCGCCAAACACCAAGGTAACAGAATGAAAAGAAAATGAATCC 237260
237261 CTCGTAATGAACCGCTATTCAACCAGGTTATCATTTTTAGGCACGAGACTTAACTACATGAGACATAATA 237330
237331 CTCGGTTACTTTTCACTGGGTTTGCCATATACTTTGCGCTCAATCTATGGTATAATCAATAATTTTTTGA 237400
237401 CTCATCCATCTCGGGAAAACGCCAGTTTGTAGGCAAAGCAGACGGATGTTGTTTGATTCGTACTTGATTT 237470
237471 GATGAAAAATGGTCCAATGAGGGCGGAAATAGGGTAAATCCAAAGTGTCTTATAATCAATCGGCTGGGAA 237540
237541 AAGGATATTCTGGACAGGTCTTTCGCTGATAAGATTTTTACTAAACCCACGGATATTTTTTTCTTTTATT 237610
237611 CCCCTTGTTAACCCATGTATCTGGTTTCCCGAGAATTATTCTGTCAATGTTTGTAACAAATTTCTTCTTA 237680
237681 ATTTGTAGTTTATTGTTTATCGGAGTTAAGTGCGTAGAATTGAAAATCAATTGTGTGCGTAGTTTCAATG 237750
237751 CGCCTTTAGTTCAAGGCGTGAAGTTTATAGAAATTCATAAGCTTTCAGTATTCATGATAAGGAAAAGTCG 237820
237821 CTCATTTCTGTCTTGGTTTATCCCTACAGCAGCTCAGCATGGCCTCTATATTTCTGCCCATTCAAAGGAA 237890
237891 TCCCATCCGGATTGCACTTGTTCGGCCTTCCGTCCATTCGGCAGTTCTTTTCCGAGCCATCATCAAGGTT 237960
237961 TGTAATGCTCTCTCTTAATTCCTCTTTAATTCTGACTTAATTACTCATAAAAACTCACGACAGAACGTGA 238030
238031 GTGCAAATGGGAGGCGCAATAACACACGACTAAATAAACCATCCGGTTTAACGTTTTACAAAAAAGTGAA 238100
238101 ACTTCAAACCTTCAGAAGTAAAATCCAAGAACTTGCGAGCCCTAATTAACATCTGCGTAGCAATCTTAAT 238170
238171 TTGGCCTCTTCCTACGCACTCGTTCCTCAATTTCCAATGATTTCTTTACCGATTCTGAGCAAAAGTTCCG 238240
238241 ATTATTTTTGTATGAAAAATCTTCAAATGACAATCGTTCTGACAGATTCGCCAAGATAAAAGTGGGTGGA 238310
238311 ATCCAAGATAAGCTAACTGTGACCTGCTGGTGTATCGATTGTACTGGTTCGCTTCGTGAATAACACTTTT 238380
238381 CTCATCTGATTAGCAATTTTTGTGGTCTTTATAGATAGCAAGAAATCGCCAAGTGTAATTTTGAAAGGGG 238450
238451 CTTTTCACTCGACGACACCTGCGAAGAAGTTTTTTTTTACTAAAATATTACTCGGTGGATATGCTAAGCC 238520
238521 TTTAAACTTTCAATTATCAGCGACTAAAATTTCCTCTTATTTTATTTTAGACATGCGGCGTTCTTTCCGC 238590
238591 AATCAATCGAGTTTTATTCTGGGTGTGTATAGAATTATTAGTAGGAAGCCTTCAATTCAGTCAATTTCCT 238660
238661 CGACTTTAAATTGCCCAGTGTTTTTTTGTAACCATTTTGAATGGGGCAGCCATAGCACCTAACATTAGTT 238730
238731 GTCGATGTATCAAAACAACATGTTTCTAAATTGAGATGTTTTCTTTACAAGACTTTCAACAGTGAATAAA 238800
238801 GAAATAGATAATAAAATATTAATTCGGATCAATGCATTAGAACAACTTAAGATTTTGAGGATAACTTGTA 238870
238871 GTCGACTAATCGCTCTATCAGAATGTGGCAAACAAAGTATAATCAGCCCCGATTTCATGTTATCTTTCTT 238940
238941 GGAAAATTCATTATCCAGTATCCCAGCTCTTTTAATGAGAGCTATTGGTCGATAAGAACACAAATTTATA 239010
239011 CAGCAGCACTGCCTTTTTGCCCCGAGCCACATCTTGCAATTAGGCTTCAGACTTCCTCATTTAAATCTCT 239080
239081 CGTTGGGCTCAATTTTCTTTTGCGCGCGTGACTAAAGCGATCGTGGAGTTGTCAAAAGTCTAGAAATGTC 239150
239151 TGATGGAAATTCGCTGAATATTCTACTCAGGGGATCCGAGGTTTTTCTCGCGATAAGATAAAAAGTTGTG 239220
239221 ACTCGCTTGGTGTCACCAGCCTAAACGCATCTGTTTTCCGTTTCATTTTTGCGGCATCTTTCATACACCA 239290
239291 GTATTTCGGCCATATCAAAAACTAGCGCCCGCCACGCACCACTTGTTGAGACGATGTTTTGGGGAATAAG 239360
239361 GAAGAAACAACGCCTTTTCTACTTGACGTTCGGTAGGCCACCACGAGAATGCACATGTAGTCATTATAAA 239430
239431 CCCAGCAAGCGCAAGCGATAACTGCATGCTGAGCCTACCCCACTACATGACTTATTATGCTCATTGGATT 239500
239501 TGATCTTTTGCAGTGATGAAAAGGAAGCTGTCGCCCGAGCAATCCCCGGACACAATACACGGCTACTCCG 239570
239571 TCAGCAGTATTGTTGGGGAATCAATCGCAGAGAAGAGAAGGAGATTTTTTAGCGAATGTGATCCGTCAGA 239640
239641 GAAGACGCATTTTCCAGCGATGCCGCCTTCACCGTTTTCCCAGAGGCGCTCTTCGCCGATTGAACTAATG 239710
239711 ATGCGT 239716

DMRT G

37688 ATGTTTTAAAATAAACTGTCAAATAGTCAGATGAAGATGAAATTTATGTCATTTATTGATGCCATCTGCT 37619
37618 AAACGAAGTGTCATGTTTCCTCGCGCTAATTTGTGTCCTGGTTTCCACCAGGTGATGTAGCAATGTACGC 37549
37548 AACATAGTTATTGCCATTCTAAATTCTAATCGTTTTCACATCTACAGAAAACGTAGAAGAATGAGTGAGA 37479
37478 CATCCACATCACCCGTAGAATCACCAGAGGGTGAAGAAGGCAGCCAGCACCACAGGGTTCCCAAGTGTGC 37409
37408 TCGCTGCCGCAGCCATGGCACGGTGTCGTGGCTAAAGGGCCACAAGCACTACTGCAGATGGCGTGACTGC 37339
37338 ACGTGTTCTAAGTGTCAACTGATCACCGAGCGACAGCGCGTGACGGCCGCGCGCGTCGCTATACTCCGGC 37269
37268 AGCAGAGGAAGAGCGCAGAGTTACGTGAGAAGTATCAACGAGAGATGGAAAACGTGCGTCTTAGTTACTC 37199
37198 CATGGTGTTCCCGAGATCGGGCATTCCGGCCTTTCCACACCACCACATGCATCACTCACTTGCGCAGGCG 37129
37128 CACTACGACGAGAGGATACGCCAGGCGTACGCCCTCAGGGAGCAAGGTAAGACGTCCCACATGGAATACG 37059
37058 AGCAACAAACAATGTAAGCCGCTAAGCAACCAAAAACAATCAAGTGTTCTAGAATCGAACATCAGATGTT 36989
36988 GGAAGACTCCAGTAGGCATACAAATAACCTCATTCCGCTATTAGTGAAAGTCAAGTAACAAAGAAATTTC 36919
36918 TATTTTTTTTCGCAGTCCTAATGTGCTTTATTGCTTTAAGGCTTTTTCTTTGAGTCAGGATTCACGCAGC 36849
36848 GCTTGCATTAGGCTAAGTTATGTTTATAATATACAGCACGGCCCCGATCCTCAAAGCGCTTCCTGGCGCT 36779
36778 TTGTTTTATTCGTTTTGTTGTTTAACTTATCCACCCACATCCCGTACTTTTGGTCCCTTTTCTCATAGTC 36709
36708 ATCATTTCGACATCTAATTTTTCAACCATGCAATTAATATAAGAAACCGACACAAAAACCTTTTGTCCCA 36639
36638 GCGCCCAAACACTACCTTTAATCGGGTTATCTAACATGCTGTTTCTCTATGTCTCAAGGGCTTGAAATAA 36569
36568 ATCGCAAGCGTTGTAGCTCCTACTCCGAGGAGGAAGATTCTGCCCCTTCACCAAAGAGACGAGCATCTAC 36499
36498 GCCAGAAGTACGCGTGAAGGAGGAACCAGTCGACCGCCCTGCGAGCCCCCAACAGCATTCAGCACCGGCT 36429
36428 AGTCCAGCGAGAGAAGAAAAGGAGAGAAAATCTACGCCTGTTCGTGAACCACTCAACTTAACGAGGAATT 36359
36358 TTCCATTCGAAAATGTCCCGCCCCGAGGTCGAGTGGACAGTGGGTCGGACAGGACATCCCCCGACCGACC 36289
36288 ACACCCAATACAGCTCCTTTCCAAGATCTTCCCAAGTCACAGCCACAGCACTCTAGATCTCATTCTTAAA 36219
36218 GGCTGTCGGGGAAGTGTAGTGGAAGCCATTGAGTGTATTCTATCAACGCAAGACCCGCGCAGGGGTGGCC 36149
36148 TTAGCTGTGCGTCCATGGCAGCCATCAAGGCAGCTGGTCTCAGTGGCTGCATTACCACAATGGCGCACAC 36079
36078 ATCACCATTTTTACACGCTCCCATAGCGCGTGCCCCAATAGCACGACCGCTGCCCACGTCACCAGCGTGC 36009
36008 TCCACCAGCCGCATCTACACACCATCGCCACTTCCGCCACCTCTCCTCAAAGCCAAGTCTGAGCCGCACT 35939
35938 ATAGGCCACCGGTGTTTTCACCACCCGGCGCCTGTTCTCTGCCCGGCTTGACTGGCGAAAGATCTAACTT 35869
35868 TAGTCTGCTCTCCAAATACTGTACACTGTGCGGACACAGGGTTCTTTCATCGGACAAATTCTGCGCGCAA 35799
35798 TGCGGAAGGGCCGTAAGCATGGCTAGCCCTGTCTGAACTAAATAACTGGTCTAGTGTGTACCGGGCCTAA 35729
35728 AGTGTAGCTAGCCCTGTCTAAACTAAACAACCGCTGCAGTCGCAAAGGCAAATCGTTGAACATTGTATTC 35659
35658 TGTAAATATTAAGTATTGCCCATTGGCATTAAAAGCATTATAAGAAATCAATGGTTCTCTACAAATATAC 35589
35588 CATAATGATCACACGACTAAAAATAAACTCTATTACAAAGGTATTCTTTTAAAGAACGAAGTTAAAATAT 35519
35518 ACTCCGTTGATGACTAATCTGAATAGCAGTTTGACTCCTCTCGGAAGAAGCAAACATAACTGAAAGCCTG 35449
35448 AATTTTTATAAATCCATCTGGTAAGAATT 35420

DMRT F

95948 AGACATTAAGCTACTTTCTGGGAAGAAAATCAAGCCATTCCACAAAGAAAACTGGCAAAGAACAGCAAAA 96017
96018 AACGAGTACAATTCGTAACGGCGGCCATCTTGGATTTTCCACAGCCGCCATTTTGGACCCTGCCAAGACC 96087
96088 TCAAACGAGTTCCACACACCCAACAAAGCAAAATAACGTTCCTCAAACACCCCTACAGTCATGCCAAATT 96157
96158 ACAAATGCCCTTTTCCATCATGTGAATACGAGACCGATGATGTTACAGACGAGTTGGCAGCTGTTCTTAT 96227
96228 CACAGTACACTCCAAAGGCTCTCAAACTACTCCTGAAGCCCAGGCCAACTGCAGTTCCACCAAAGTCGAG 96297
96298 AAGGTCCGACGACCAACGATAACAACCGCAGGGACAAGTGAAGAGTGGTCTTACTTCACAACCAGATGGC 96367
96368 TAGACTATGTTGAGGCCACACATATCAAGGGCAAAGAAAAAGTTATCCAACTTCTTGAATGCTGTGACGA 96437
96438 GCAGCTAAGAAAAGACCTTACCCGCAACGCCGGTGGTTCCCTTACAAGCAAATCTCCTCAAGAGGTCCTT 96507
96508 GAGGCAATAAAGAAGCTGGCTGTCAGAGAAGAAAATGCTATGGTAGCAAAAGTGCAGCTACACAACATGA 96577
96578 GGCAGGACATAGATGAGACAATAAGAAGCTTTTGTGCCCGCCTCCGCGGCCAAGCCGGCGTATGTAAATT 96647
96648 CCTTGTGGCCTGCCCGGACTGTGCTACAGCAGTTAACTACACAGAGAACGTACTCAGAGATGTACTAACC 96717
96718 CGTGGCTTGGCGTACGAAGAAATTCAGCTTGACCTCCTTGGATGGAAGAAGTGTTGCAATTCATCGAAGC 96787
96788 TAAAGAGTCTGGCAAAAGCTCAGCAGGGCGGCTTCTTCAAGCCCAAGGTACAGATGCAATCCGCAGTCAG 96857
96858 CACAAGAAGCAGAACACGAACCGGAGACCACCCGACAAATCCAATGAAGCCTGCTATTATTGTAGAAAAA 96927
96928 CAGGCCACCGCTACAAAGCGCCACCCAAACAACGCAAGCAAGAGTGCCCGGCATTCGGTTCTATCTGCCG 96997
96998 CCTATGTAGTCGACCGAATCACTTTGAGGCTGCATGTCAAAGTAAAGAAAAGCCCACCCCCAAAGACCCT 97067
97068 CGCCCCCAGCAGGCAGGCAGGCCGAAGGTGCAATCTTTGAAAACCTGTGCAACGTGACAAGGGAAGACCA 97137
97138 CACCCTCAACGGCATTTCCCTAGAACTCCACCCGTATAATAACATGAACGACTGCTGGATCCGACGCGCC 97207
97208 TCCCAGCCACAGCCATTCATCCCTCTCAAGATATCCATTCATCCTCATGACTATGTGGCCTTAGGATAGA 97277
97278 AGCCGGTCACTCACACATTCAGGTCAATCGAGCTACCCGCAATGGCAGACACCAGCTGCCAAAGCTGTCT 97347
97348 GGCCAGCTTGAGCCTCATTCGCCGCCTTGGGTTAAGTGAAGCTGACCTCATCCCTGTCACTACACGCATG 97417
97418 CACGCAGCTAACAACAGTGGCATCAAGATCCTTGGCGCTATCATCCTACGAATGACAGGCACAACTGAAC 97487
97488 CTGGACATGACCTTTAGACAAGACAGATCGTATACGTCACAAATGACTCCGACCGCCTGTTACTCAGCCG 97557
97558 GGAGACCTGCCAAGAACTGGGAATTTTATCAGAACGATTCCCCACAGTAGGAGAGACACTCCCCACCCAA 97627
97628 ACCCAGAGCACCATATCCACATCAACTGGATCAGCAGACCAATCACCCCAACAGCCCAAACTATGTGATT 97697
97698 GTCCACAACGCCAGGCACCACCGCCTAAGCCGTCCACACTTCCATTCCCAGCAACAGAGACCAAGGGGAT 97767
97768 CCTCAGAGTCGACCGNNNNNNNNNNNNNNNNNNNNNNNNNNNNNNNNNNNNNNNNNNNNNNNNNNNNNNN 97837
97838 NNNNNNNNNNNNNNNNNNNNNNNNNNNNNNNNNNNNNNNNNNNNNNNNNNNNNNNNNNNNNNNNNNNNNN 97907
97908 NNNNNNNNNNNNNNNNNNNNNNNNNNNNNNNNNNNNNNNNNNNNNNNNNNNNNNNNNNNNNNNNNNNNNN 97977
97978 NNNNNNNNNNNNNNNNNNNNNNNNNNNNNNNNNNNNNNNNNNNNNNNNNNNNNNNNNNNNNNNNNNNNNN 98047
98048 NNNNNNNNNNNNNNNNNNNNNNNNNNNNNNNNNNNNNNNNNNNNNNNNNNNNNNNNNNNNNNNNNNNNNN 98117
98118 NNNNNNNNNNNNNNNNNNNNNNNNNNNNNNNNNNNNNNNNNNNNNNNNNNNNNNNNNNNNNNNNNNNNNN 98187
98188 NNNNNNNNNNNNNNNNNNNNNNNNNNNNNNNNNNNNNNNNNNNNNNNNNNNNNNNNNNNNNNNNNNNNNN 98257
98258 NNNNNNNNNNNNNNNNNNNNNNNNNNNNNNNNNNNNNNNNNNNNNNNNNNNNNNNNNNNNNNNCGACACCA 98327
98328 GCAGCTCCCTCTCATGGACAGTGTCCCAATGCGACCCATGGTTAACCCTGATTCAGAGCCAGTGGCCCAC 98397

98398 CATACCACCATCCCTGTGCCACTTCATTGGCAAGAGGAAGTAAAAGCTGGTCTGGATCAAGACGTCGCCC 98467
98468 TAGGCGTCCTGGAGCCCGTGCCAGTGGGAGAACCAGTTACATGGTGCCATCGCATGGTTGTGTGTCCAAA 98537
98538 GAAGAATGGGAAACCACGCCGAACAGTCGACTTTCAGTCCCTCAACCTACATGCTACACGCGAGACCCAC 98607
98608 CACACACAGAGCTTATTCCATCAAGCTCGCTCCGTGCCCCACAACAAGAAGAAAACCGTATTTGACTGCT 98677
98678 GGAATGGATACCACAGCGTCCCTCTGCACGAAGATGACCGCCACCTCACCACCTTTATCACCCCCTGGGG 98747
98748 ACGGTATCGCTACAACACGGCCCCCCAGGGATACATCGCATCAGGTGATGGCTACTCCCGAAGGTTTGAC 98817
98818 GAGAATGTATCCGACATCCCAGACAAAATCAAATGCATTGATGACACATTGTTGTGGGCAGACGACATAA 98887
98888 GCAGCAGCTTCCACCAAGCAGTCAACTGGCTTGGCATCTGCGGGCGACATGTTATAACCCTCAACGCCTC 98957
98958 CAAGTTTGTCTTCGCAGCCGACACAGTAGAGTTTGCAGGGTTCGAGATCACAAATGACAGCGTGCGCCCA 99027
99028 TGCACGAAGTATCTCGATGCGATCCGCCACTTCCCTATACCTAGTAACATCACTGACATGCGCTCCTGGT 99097
99098 TTGGTCTGATCAACCAAATGTCTTATGCCTTCGCAGCAACAGAACTCATGCTCCCATTCCGCGAGTCACT 99167
99168 GAAACCTAGGACCCCATTCTTGTGGAATGACGAACTCAACCAGCTCTTTGAAGAATCCAAATCAGCGATC 99237
99238 ATCAGCGCGATCGAAGATGGCGTCCGCATCTTTGACAAATCAAAGCCCACATGCCTGGCCACAGACTGGT 99307
99308 CCAAGACTGGCATTGGGTACTGGCTCCTCCAAAAGCACTGTCAGTGTCCCTCTACAGAGCCCTTCTGTTG 99377
99378 CCGCAGCGGTTGCAAGATGACACTCGTTGGCAGCTGCTTCACACATTCAGCCGAGTCACGCTACGCCCCT 99447
99448 ATTGAAGGCGAAGCATTAGCAGTCGCGGACGCACTCGACAAGACACGTTTCTTTGTCCTTGGATGCAAGA 99517
99518 ACTTAATCATTGCAGTTGACCACAAGCCTCTGCTAAAGGTATTCGGGGATAGATCCCTAGACGAAATCTC 99587
99588 CAACGGTCGACTAAGAAACCTCAAGGAGAAGACACTTAGATACAAGTTCAGATGGTACACATCCCCGGTG 99657
99658 CACGACACAAAGCTGCAGACGCTATCTCGAGGCGCCCAACCGGCCCCACAGACCCTGACAAACTATCACT 99727
99728 ACCCGATGACATAGCAGTTATTGATAACCTAACTCACTCAGCATGCCTACCTGGGCACCCCTTCCTGGCA 99797
99798 AGCATCCGCACCAAAGAACAGCCACAAGATTCATGCTCCTACAGCATCGATGACGGGCTAGTATCATCAG 99867
99868 CCTCATCCATCCTCAACACAATGGCGGTCACTTGGGAACGTGTCAAGCTAGAGACAACCAGCGACCCAGA 99937
99938 CTTACAGACTCTGACCACCATCATTGAATCAGGATTCCCAGAGTTCCAGCATGAGCTCCCACCTGCCCTT 100007
100008 CAGGAATACCACAGGTTCCGCCAGCACCTCCATACCATCGATGGTGTCATCCTCTACAAAAATCGCATTG 100077
100078 TGGTTCCCACCTCTCTCAGAGATGCGATATTGTCAACGTTACATTCTGCCCACCAAGGGGTCACATCAAT 100147
100148 GACAGACCGAGCAGAGTCACCCATCTTCTGGCCAGGCATCACACCTGCCATCACAGCACTACGTGAACGG 100217
100218 TGCTCCCACTGCAACCGAATGACTCCTCCACAGCCAAGTGCCCCACCGTACCCATCAACACTACCAGCAT 100287
100288 ACCCGTTCCAATGCATCTGTGCTGACTACTTCCACTACAAAGGGGTCAACTACCTTGTAGCAGTAGACAG 100357
100358 ATATTCTAATTGGCCAATTGTTGAAAGAGCAAGGGATGGTTCTGCTGGTCTTATTGAATGCCTCAGACGT 100427
100428 GCCTTTGCAACATATGGTGTCCCCGATGAATGCGCAACCGATGAAGGACCACAGTTCACTGCACAATCAA 100497
100498 CACAACAGTTCCTGAAAGATTGGGGAGACTATCACTGCCTATCCTCAGTTGCATTCCCACACTCCAACTG 100567
100568 CAGGGCCGAGATCGGGGTCAAAACCGTCAAACGACTGATCACCAATAACACAGACCCACATGGGGACCTG 100637
100638 AACACCAATGAATTCCAGAAGGCGATTCTACAGTACCGAAACGCCCCCGACCCGAACACGGAGTTGTCTC 100707
100708 CTGCTCAGTGTGTCTTCGGTAGACCCATTAAGGATTTCATCCCCATACTGCCTGGGCGCTATCTCCCCCA 100777
100778 TCCTACCTGGCGCGATACCCTCGCGTTGAGAGAAGCCTTACGAAACCGACACATGCAAGCATCAGAGAGG 100847
100848 TGGGCTGAGCATACCAAAAGGCTCCCTCCCCTGGTAGTTGGAAACCATGTCAGAATCCAGAACCAGACCG 100917
100918 GACCTAACCCCACATGAGGGAATAGATGGACTCTTATCTAATTTCCAAATGTTGATTACAGTGCTACAAT 100987
100988 TTCGTAAAACAACAAAGAGCGCAGCAAAATTCAAAGGAATTATTGATTCAAGTTCTCTTTTGTCTAAAAC 101057
101058 AGCGTTAAAATTTGAAACGATTTTAGGTAAGCAGAATTTCAAAACCCGCGCACACTTTTTGAAAAACGCA 101127
101128 CATTTCCCTATAAATTTCTAAAATTTGATTAAAGTGCTAAAATTCCGTTAAACAGCAAAGAGCGAAGAAA 101197
101198 AATTCATGGGAAGCATTGATTCTAGTTCTCTTTTGTCTAAAATAGCGATAAAATTCAAAACCATTTTAGT 101267
101268 AAGCAAAAATTCCGAAAGTCGCGCACACTTTCTGACAAACGCACACATTTTGCACACGCACATTTCCCTA 101337
101338 TAAATTTCCAAAAGTCGATTACAGTGCTAAAATTCCGTAAAACAACAGAAAGCGCAACAAAAGTAATGGG 101407
101408 ATTTTTATTCCATTTTTTTTTTATAAAAAGAAAAATCGAAACCATTTAAGTAAACAGGAATTTCGACTTC 101477
101478 CGCGTACCCTTTGACACTTCAGGACAAACACACACATTTTGAACGCACATTTCAAAAATTAGTCTCAAGC 101547
101548 GCGTGTGACCATCCAGCCGTCAGTGGGGCCGATATAGGCCTGGGGACGTTTTGTTCTATATGGTCAGGGG 101617
101618 AGTGGGACTCAATGTGTCGCTACCCAACTGCAAGCAGTAAATCTTAGCAAAAGGAAAAGGAAAGAAAAAG 101687
101688 AATACGAGGTGAGCGAAGTGTTGTTTACAGACAAAGCTATAGAAAATGCTGCCAAAGTGCTACTTTCGCG 101757
101758 GAGTTTGGATTCACATTTTTAACCCTAAAGGATAGCCGGGAATCGGGAAAACTGTTAAATACCTCCTAA 101827
101828 GGGAATAAGCTGGCCGAGTTTTATACCCTAAGAGATAGTAGAATCGGAAGAAAACGTTAAGCACCGCCAT 101897
101898 CAGGAATAGCAGTTGGTCGAATTTTATACACTTTAAGATAGCAGAATCGGACAAATATTTCATCATAAGG 101967
101968 GACTATAGCAGTTGGTCGAGATTTTTTACCTTAAAAGATAAGGACGATCACCCCTGAAAAGAGAGGAATA 102037
102038 GGGACGGGGTTGCGTTTTTACTCTTTCTTCCAGGAGCAAGGTTGAGCAAGCATTTTGGTTTTTTTAGTA 102107
102108 CACCCGGGCTAGAACGGTGAGCCTGTGCTCGATACTTCACTTTATTCCTTAAGAATTCTTCATTGCTCTC 102177
102178 GGGAGTTCCTTTATCTTATTATCAATATTGAATATCGTAGTAAACGTTATATCTGTATCGATTAGATACA 102247
102248 AGATATCAGAAAAGAGACGAGCAATGGGGAGAGAGGGTGAGCAAAGAACATGAACTTGGCCGAAGAACGG 102317
102318 AGAATATGGAATATGGAATTGACTATGCTAAGGGATTATGCGTGCAACGGGGTGCAGGCAGAGCTAAATT 102387
102388 TTGCCATTCTTAGCGTGTTTAGAAAACATGTTTTGTGGTGCGATTTTGCCTCATTTCTTCGCACTTTTCT 102457
102458 TGGCCGAGAGCATTCGTATTTCAATTAGACATTCAGTTAAATAATGAAGAGCATTGTAATCTGGAAAGAA 102527
102528 GCACCCAAAATGGGGAGAAGAGGAAAGGAAGACAGGAAGAAAAGACAGTGCAGAAAATATTCTGTGTAAG 102597
102598 TTCTGTACTGAAATAGCACACAACAAAGCAGGACATTAGAACTAAACATAAACAGCAAACATCAGGTATA 102667
102668 AAGTCAAGATACATCATATATATTTTATTATTTATAAGTCACTTTACAGCTGTAGTGTAAGAGGACACTT 102737
102738 CAGTAAAACTTATTCTAGTGAAACCATCATTTCTACTTGTTGCTGCAACAGGTGTAAAACAAAAATGCAA 102807
102808 AGTGTGCCTATCTGCTGTTGTACATATGTTTAAGAATTAACCAAAAATCCATAAAGTGTTTTGACATTTA 102877
102878 CCTAAGCAGGAAGAAAATTTGGTGTAGTGGGCAGTTCAGGGGATGTTGAACCCCAGTTGACTTCTGCTTT 102947
102948 GACAGAAAGTCAATTTGTCAAGATTTATCTTTATTATCCTTTTACCAAATTTAATAGTTGATCAATACAT 103017
103018 GTTTACCATGATCAGGCTTCGGCAACTAACCCCAGTTTCATAAGATATTTCAGCACCATCCAGGGAATGT 103087
103088 GCAAGTGAAACTCTGATATTACATTATCTATGGTGGTCATTACCTTGGGTCCTTTCTCTGCTGCCATCCT 103157
103158 GCTGTTAGTACCAGACTTCTTGATCCAGAAGATAAAGAAAGGCTAAGGGAATTTTTGTCAACTTACCCTC 103227
103228 ACCCTGTCTGTCATTTATAAGCATACTCAACAGAGTACTAGGTACACATGTTTTAATAGGGAGTATAGTG 103297
103298 CAGTCTACTTAATTTGTTGGAGAGCACTTGGTCATTAGACCATTGTGCCCTCGTGCTAATTTATCAGATG 103367
103368 CAGGGTTAAAAGTAGGGCTTAGTTATGTCCCAATAAAGTGCAACACTTACTATTTGTCTGTACAAAACAA 103437
103438 GGGAGTGGTATACAATAAAAGAAAACATTTGTTTCTATATTTGTTTTTATAGTGCATAACTGGCTCACAG 103507

103508 AAGCAGCAGAAAACCCCTTGTACAATGTCTCAGCTTGTATACTTACACATATATGTACACCAAACAACTG 103577
103578 AAGGGCTACTAAACAAATGCCTCAAGTGAGTTGGAGCTATAATTGAGTCTATCTTTCTACTTTTTAGATC 103647
103648 AGAATGCACCTGTATACTATTCCTACGCGGGGAAACTGTAATGTTTCTAGTACAGCTTTCTATGAAGGAC 103717
103718 TAAATTCGACGGGAAGCGTGGCGTACGATTGCGTATAATTCGAGGTCGATCGAATGGTTGACCTAGCGTT 103787
103788 ATCGAGTGCTCTTTTGAACTTCTACGGTTAGATACGCCTTGTAAGATGTTATTTAGAGTCTACCAGGACC 103857
103858 ACGTTTCGCATTTATATGAACTTGTTTTGGCGATAAAAGGTGCCCCGATCAAAGGTATGTGCTCTTACGA 103927
103928 CAGCCATTGTCTTGCAATTGTTGAAATTGCAAGTGGGGGCTGTTTATCGCCTCATCAAAACTCGTGTTTT 103997
103998 TAATTGCCACAAAATTTTATTTAATATATTAGAGGTACAATAAGTGCTGTATACCTAGAGTGAAAAGAAT 104067
104068 TGTGCAATTCCACGTCCCATGGAAGATATAGGCCTCGTTTTCCGGGGTTATTTACGGAGTACGAAAAACA 104137
104138 TAAAATAAATGAAAAATCTGTTAAAAATTAAGAAAAATACTAAAACTACCAACGACCTAATCCAAATATG 104207
104208 TACAATTATATAATTTCTATTAATCAAGTTAGATTTTTGCTGCCCGGCTTAATTTTGTATGTTTTTTTAG 104277
104278 ACATTTTCTGTTCAAGTCGAAAAATACCGAAAAATCGCCAAATATAATCGAAAAATACATTTTCCCCTCT 104347
104348 CAAGCCAAAAATATTTATACGATTCGTTTCAAACATTCACAGAATACTAAGGTCATCAAGGGCTATCTCC 104417
104418 TGGCAAAAGATGGTATTTTTGTTCTGAACAAAAAATTTCTTCGTCTGAGACGGTCACTCTTAAGGGTGAC 104487
104488 CTGGTGACTAAGCTGTCATTAAAAGTAAATACTAATACTACGAAAATTCTTGGGTGCGTCAGGTTTTATA 104557
104558 ACAACTTGGGTCTGCTGTTTTTATTTCAAATTCCGACGTTACAGCGTGTGACTTTACATTTCAAATCGCA 104627
104628 TGATTTATAATTTTTTGGGCGCTATATTTACCCACACCCGATGCCTGTGCTAAGGAAAATATGAAATGAG 104697
104698 AATCTGAAATGCTCCTAAGAAATTCGATGAGAGTAGCTTACGAAAGGTTAAATCACATTATTTTCTAAAG 104767
104768 TCTAAGAGCACGCACAAAACAATATATTGCGTGCATTTCAATGTAGCTTGCTTTTGTTGTTCTGGGCACG 104837
104838 AGAAATGTCATATGCACGTGCGTGACTGGACAAAGAAGAACAAAGGCTAAAGGAAGCAATGCAGTAGTAG 104907
104908 TAGTAGAAACTTTTAGTAGTAGAAACTTTATCAAAGTGTCATAAACAACCTCTAGCCGGGGATAAGACCC 104977
104978 CTACTAATTGGGGGACACAAAAAAAAAGATAAAATTATAAAAATTATACTGATTATTAAAAATTTAAAAT 105047
105048 TGTAGATTCTTACTGATAAAGCTAAATAAATATATATATATATATATAAATACATGTAAATTGATTGTAA 105117
105118 TCTATATGAAATTATGAAAAAAAAAAAATTTGACTAGATTTAAAAATTATCATGCGGAACATAGGGCACA 105187
105188 GCCTTTGCAATTTTCTATAAATTCGAAATCCTTTTTTCTCATAGTCACTTTAAAACTTTTTAAACTGTCA 105257
105258 ATTGATCTTTCTGCCCTAGGTAGGCTATTCCATAGTTTAGCCCCCATATATCTTAGGGAATGTTTCCCGT 105327
105328 AAGTGACTGTGTTAAATCTTGGAATAACAAAATCGGACAATCTTAAATTATAGTTCGTGTTTTTTTTAGA 105397
105398 AAACAAATCACTAATCCCAGATGGACAAAGGCCATTTTTAACTTTGTACATTAAGATGCAAATGTCTTGC 105467
105468 AGTCTACGATTACGTAGAGTAGATAAATTAGCCTTTATTAATAGTTCGTGGTAAGTGCAATTCCAGTCTT 105537
105538 TAAAAACAGCTCTTAAGGCACGTTCCTGTAAACGCTCAAGCTTCCTACTATCGCTTGCCTTACAAAAATG 105607
105608 CCAGGTCAAGTGGCAGTATGTCAAATAAGGCAACACAGCAGATTTATAGAGAACTAGTTTAGCGTTTGTA 105677
105678 GGTATCAAGTTCCTGAGTCTCATCAGCACACCGATTCTTCTGCCAGCATTTTTACATGCTGTGCTTATAT 105747
105748 GCTCCGAGAAATTGAGTTGAGAGTCTATTGTAACTCCTAGTAGCTTTAGGGAGTCCGCTGACCGTATTTC 105817
105818 CTGGTTGTTGATAGAAATGCGTGAGATCGAGTCCACGGAGGCGTTTGCCTTTTTACCATAACCGATGCTT 105887
105888 AGTGTTTGGTATTTCTTGAGGTTACCAGCCAACAAATTGGACTCGTACCACGCAGTCGCGAGTTCGGCAC 105957
105958 TTGCACTGAGCTTTGCTGTGACCACTCCCGGATCCCGACCGCAATGGTAAATTTGATGATCGTCGGCGTA 106027
106028 CATTGATAACTCCGTATTGACACTCATTGGGAGGTCATTCTGAAAAATATTCCAGAGCAATGGTCCTAGG 106097
106098 GCCGAACCCTGAGGACAGCCTCGGTTGACCGTGCTGTAAGAACTTAGATCCTTTCCCATTCTAACACGAT 106167
106168 TTCTTCTGTCTGAGAGATAACTCTCAAGAAGTCGCAGACATTGGTCGTAGTGTGCTGCACATGACGACAT 106237
106238 CACTACATTACTATAGCAACACACCTATTCCCAAGAGACACAAGCGTGAAAAAGACCCCGATATTTTTCA 106307
106308 CTGTATATCTCAGTGAGCTGTGCGTGGCGTAACCGACTAAGACAGGGCTGGGATCCTGGTTATTGCCTCC 106377
106378 CCATTAATGCGCGGGTACCTCAGGTTCCTTTGTGTTGTACAGTTAGCTAGATAGCGGTGCTGGGGCCGAA 106447
106448 ATCTCCAGCTTAGCCGTAAAGCTATATGCTGCCATACGGTGTTTCACCGCATGTCTGGAAGTATGGTCTA 106517
106518 TTTTTCTCATTTTACCGGATTTGGACGAGGTTCGATTCTCGACCATGTCCTCGAATTGTTTTTTTTTTT 106587
106588 TCATTGTTAACATTTACTATCTCATTCAACATTAATTAAACACATTTACTTATCCCAGCCCTGTCGTCTT 106657
106658 TACGTGGCAGTCGTGATATTGCAGCGTCTAACTAGGATTTGCCCACGGTTACCAGTATGTAGTCTTGACG 106727
106728 TTTACATGGCAACACATACCACAGGGTGACCATAATAAAAATGTCATCGCTAAGGATCTTGAATTGATAC 106797
106798 AGCACAGGGTACCCAAGACTCTTATTAGGGCTAGCAGGCCTAAGGCCTAGTTTCAGGATTAATTATTATC 106867
106868 CAATATGCTTTTCAAGTAAATTTTATATTGAGAATGTACCAACGCACTGTTATACCAGATGCACTTTATA 106937
106938 ATTTTTCGTCTTTTGAATTTTCCGTCTTATCAAATCTTGAGTATGAAAGGTATTACCTAGATTTTGGGTG 107007
107008 TATTTTATTTCTAAGGCGCCATATTTCGCGATTCTTAAAGGAATCAGAATTGAATTTCATATCAGGTAAC 107077
107078 ATTTAACGTGATCTTCATTGTAAATATTCATAACAAATAAGGTGTATGTAAAACTACTTCTTGGCAAATA 107147
107148 CTACGATGAATATTCTGGGGTGCGACAGATTTTATTAAAATGAATTAAAAACCTAACAAAGTGCGCCAGC 107217
107218 TTTTTTATAACAAGTGCGTCGTTACGTGTGATATGTTTGGACGATATACTTAATGCCTGTGCCAAGGAGA 107287
107288 ATATGAACTCTAGAATCGAAAATGCTCCTAAGAGACTCTAGAAGAGTAGCTTGCTTACCTATACTTTCCT 107357
107358 TTTCCAAGGATGAGAAATGGTACGAAAAATAAACAGTTTATCACAGTGAGCCAAAAAAGGTTATTATTTT 107427
107428 ATAATAACCCAATGAATTATTGTAGGAAGCTTAATAGCAATAACAAAGTTGTTACTATAATCAGCACAAT 107497
107498 TTGATTCTGCATTTTAGAACGAAACAGGTCTAGAATTTTTTTTTTTTCTTTTTTGCTATCCGAGCCTCGGA 107567
107568 ATGTTCTAAGCATGTTCTGAAAATTTGTTGAAATCTCTGGCTGGACGTTCTTATAAAAAGAGTGCATTTA 107637
107638 TCTTTAATGTAAATATAACATCAACACTTTTCATAGTACAAACCTCTTATGTTAAAAGCCTATTTTATTT 107707
107708 GACCTTTTATATACATTATCTCTCAAGTGGAACCAGCTACTATCTCGACCCATTTTTACTTTTGGCGTGC 107777
107778 AGTTTTCTAGTGACATTTATTTACAACACAAAAAGTGATCCCGCCACCATGTGACGAAAAACAAATGTGC 107847
107848 TAAAATAACAAATTTAAAAACTGTAAACATACTGGAAGCAGGGCTAAGGTCATACTAATTGATATTAAAA 107917
107918 TGGATTCCCAATAATTATACCAATTCCCAATAGGTATACAAAATAGACTGTACAAATTTTTTTTTATCCC 107987
107988 AAATATTTAGAGTTCCTTCAATAATAATTGATACGAGGGGTGGTGGTCTGCGCGCCTGATTGGTTGGCAA 108057
108058 ATCATAATTGTGTTGGAAATAGTTTTCCATTAGTTTCATAGACATATATAGGTTAACGTTTTCATGGCTC 108127
108128 GGTACACAACGTTTGAACAACAACATATTTTCACTTATGGTATCACCCCACCCCTACGGGTGCAAAATCC 108197
108198 AAACAGACAATTTCCCCACGCTGGCTACTCTAAGCGTCCTAACACTGCTCTCTTCATTCCTTTATAGATA 108267
108268 GAAAGCTTTTAGCTTTCGTGAGTAGTCTCAGACAATAGGATTTCAAGACAAGACTTATATCCTATCAGAT 108337
108338 TTATACAGTTCAAAACAACAAAAAAACATGCGTCTCCGGGCCGATACTTGAAAGGCACAACACTTAACAA 108407
108408 GTCATGCAAGCAGTTGTTGTTCAAAACTGAATGGAATGTGTGTTGATTAAGCTGCGTGGGCTCAGAGAGA 108477
108478 GATAACGTGAGTTAGAAAGATTGACGAGTCATGCTTAGTTTGCCCTGTACAATACCGCTTGGTTGGAGCC 108547
108548 GACGAGAATGTATAAACGGCTTTCCTGTTACTCGGTGATTAAAAATACACATAAGAATGTGCTTCCTATC 108617

108618 ACGTTTAGCGTGGAATAATCCACGCGGATATGTTACAAAGAAAAAGGAAAAAAGAGATGTACTTAGTGAA 108687
108688 ATATTCCTAGCTATTAATAAGACAACCAAATAGAGTAAATGAGGACGCGACTCTTTATATAATTTTCTAG 108757
108758 TTGTCGAATTTGAATGATGGACAGATATCGAAACATGGTCGGTCAAAGTTAAAGACGACAGGCGATCAAT 108827
108828 AGCACCACCAGCTTTATTTTTCGAAAACACCAAAGTTGCTTTGTCTGGTTGTCAGTCAACCCATGAATTA 108897
108898 TCAATCGCTTACCTCGGTGCCACTCTCTGAATAGCGGTTGCCACTTTGAATTTTCTTTAATTATTTTAGA 108967
108968 TTAAATTGAATGTAAAAAGATAATTTCGTTGCGGCTTTACAAAAAAGTTTATTCAGCTAAAAAGTTCTT 109037
109038 TGACAAAGCCGATTGACTTAAATATGTAAACATTTATCCCAACTCAAAGCAGCCACTGGTTACAGTTCAA 109107
109108 TGTAATATTCCCCCGTGGTTGTTAGTAGGGGATTATGGCGTTATTTATTCGGCAAATTCTACGCTTCTGC 109177
109178 TATTATCACCAAAGCCGCAAGATTGCCTTCGCGTGATTCCATTGCAAGCAATTTGCCCAAACTTTTGAAT 109247
109248 CATTTGCATATATTATTGTACGTTTTGTGACAAAGAGCGATGACGCCTCAAGTGCCTGCAAAACAATGAA 109317
109318 ATCATCTCGGACAATGTAATTGTTCCTGAATATTGTTCGGCTTGCGGAAAGGCAACTCAGCACACAAGAA 109387
109388 GTTTCGAAAACGCTCCCAAACTACGTGTGCAAACAACGAGTAGAAGACGTTGAGGATCAACACAAAGTTT 109457
109458 TCACACTATATGAAACTTGGGCTTATTGATTTCCCATTTGTTGCGACGATCTAAACAATTCGAGGTAAGT 109527
109528 GAATTGATTTTGAAGTAACTTAATTTGTACTTAGCAATAAGTTGGTAAAACTAGTTGCATTTATAAAAGC 109597
109598 ATAGAGTCCCTCGACAAGTAAAGTAAAAGAAAACACGTTTTTACGTGTATTAAACTGGCTGAGTGTTTAT 109667
109668 TTTGTAATCAAATTAAGTATTTTCAAAGAGTACATATTTGCTAAATTCTTACTAAACAGAAGCAGTTGCT 109737
109738 GGGGTTCCATGTTTCAAGTTGTCGAATGTTACAGGATTTGCGAAATTCCAAAAACGCATCGCCTAAATAA 109807
109808 TTATCGCTTATGAAACAATATCTTAGATTTAGTCGACTTCCAGGATTTGGCTGAGAGTAAAAACACACCC 109877
109878 TTGCAAATGTGAAATCATTTGCGTATCATCATCACAATTATGCAAGCTAGACCAGGGTTCCGTTCAATCA 109947
109948 AGCTTGGTAAAATTTCAGGTTGTTTAGCTTTCGACATCAAAGTTCTGGCTTCTGAAGCAATTATAACTTT 110017
110018 AGAAACACTTTGCAAACTTAGCGGGTGTGTTTTTCAGTTACTCCGTAATTTCTTAGCATTGTCTTATTTT 110087
110088 ACGTGAGCGCGCTGTGTCTAGAGGTAGCAGTAAACTTTGCCATCCAGGTGGTCATGACGATAATCATGTG 110157
110158 TGCTCAGTCCTAAACCTGTTAGTTGTGTACTCTCATAATAGAATTTCACACCTTGCGCTGCGCGCCCACT 110227
110228 CAAATTTTATCACCTTTAAATTCTATACCATCACGACAACCCAAGAAGCCAACTAATAAAACGTTACATA 110297
110298 ACAAACCTTCGAAATTAATGATTTAGTTTATCATTAGATTTTCAGAGACTTTCGTGAGTTTTAGCGTTAT 110367
110368 CGACCGTAATTAGAAATTCTTTTATTTATTTATTTAGTATCGAAACTTTAACAAAGTAGTTCGATTGATTTCCG 110437
110438 TGTTTAAGTAATATCTTAAGCGGCCAGTAAGGGCTGTGTTGCCACTATCGCCTTTTTCCAGCCATTAATA 110507
110508 TTTGAATACCAACGGGGTTAGTAAAATGTCTGCTCAGGGGATTAAAGTAATTTCCTACTGAGAAGTAAAA 110577
110578 TATAGTACTTATGAGGTGGGTAGCTATTTATAATGTTGCGCTGTGTGTATATTGGTAACGCTCAAAACAA 110647
110648 AATAAATGGTTCGATAGTTTTATTAAGCGTAACTTTCAAATCCTCAAATGTTATGAATTTTTAAACCATC 110717
110718 AAAATTTTCTTTCTAATGTTGTATTCTTGGTGAAGTGATAAAGGTACTTGTATTGAATTTCCAAACTTCC 110787
110788 GTGATCAGTAGTTAAAACAGATGCACGCTAACACCAAAAAGGCTTATTACTTACTTACTTTATTGCCATT 110857
110858 TATCACTGGTTTTGGTTCCATGGAAATGGAATAAATAAGAAAATATCTCAACTTAAACAAGCGTTTGTTT 110927
110928 ATCCGTTTGTTTATTCTAAGCATTACAATTCTGGATAAGCATCTCAGCCAAATAAGAAGCAATTACACTT 110997
110998 TAGCCAATGTTAGCTTGCGGCGTACCCGTCTCTCTCATAATACCAGTCCACTTATAAATAACACATCGCG 111067
111068 CTTTTGTAGAGGTCCACGAGAAAATAGATGGATTGAATGTCAAGAGACCCCGTTGTTAGGCGCCCTGGTG 111137
111138 TCCCGAAAAGCAGGGCCATTTCTTTTGTTGAGGTGGCTCTTGGGTGCTGAGATGTTTCAATCAAAATAGA 111207
111208 TTTTTCAAACGGACGAACTATTGCATATTCTCGTCAATAACAAAAGAATAGGTCTAGCGTACGCTAACAA 111277
111278 AACTGATGGAGATTTCATGATTTTGTGAACTTTGAACGAAAATCATTTAAGTTTTACTAGCCAAAGTGCA 111347
111348 AGCTTTTATAGTTTTGTGATATCTCCTAGTGCAATCTTGCGCGTGGCGCCGTGCAAAGCATGTACACTAC 111417
111418 CCGCCATGACGACTTATTGTAAGATACTGTTTCCATGTCCCGGAGTGGTCTCACAACAAAGTACCTGAAC 111487
111488 GAAAACAAAAAGGAATCTAGATGCTTATCTAGCGCGCCTTGCATTCTATACCAAACGGCATTCAAAATGC 111557
111558 TCCACAGGCTTAAACCATGTCACCTGGACCCTCAAGTTTGTACAATCGTTGATCAACTGACAGACCGACC 111627
111628 AATACACTTTACCTGACACATTTGCGCGATAACAAAAGTGAAATGAAAGGAAAGACCTCATGCAAACACA 111697
111698 ACTAAAGGGCACAGCGCGAGTTTCACTGCAAGTTTTAGTCAAATCAGCCAAAATACCCTTTTTGTTTCAT 111767
111768 TATTTTAATGTTTAAGCTTTAACTATGAGCACTGTAAAATTAAAGTACTTATTAGTTGTAATTTGGCAGT 111837
111838 GACGCGCCTTAGTAATCAATGAAGCTGTGAGGCGCTGTGGGCACTTAATGCATTTATTACTCTGTAGTCA 111907
111908 CAGTCAAGTGAATTTATGGTATTTTAATTTGGTTTCGACAAAGCGCGAGTGCTTACGTCTATACAAGTAT 111977
111978 TAATCACCGAATGACACGAACGCTCGGTCTTTGTTAGGGTTTTGCGCGCTAAACCGGGCCGAGGGTATTT 112047
112048 GTGCCTTCGTTTAATAAATTAAGCAGTTTCGCTTTAGCGATCTGTTTGTTGTTTTCGTCTATTTTGTTTA 112117
112118 TTTGTCTGTTTGCATGCGCCATCCGTTTCACTTGCTAGATATCATGATATTATAAAGTCGTCGAAAATTA 112187
112188 GGTAAGTTATTCAAACTGTTAGCCACCTTTTCACTTATGTAGCTGCACTAATTAAGTATACAAAAAAGGA 112257
112258 AATCTTTTAAATTTTCATTCAAAATTCCATGCAGATAATATTAATATTTCGCAGTTTTTGAAGAGGAATA 112327
112328 ACACAATTGTTAGCACATTGTGCGAGGCTGCTTCGTACTCTGACAGTCCTGAGACACGCAAGATGAACTC 112397
112398 CAGTACCGCGCAGCTCCGACCTTTTTAGTTTTTGTTGATGGCGACATTTTACCGACACACTGTCACATTT 112467
112468 CCGAGGACAATAATTTTGTGCGCAGACATAGGTATCATATGGACAAAGCATAGTCTTATAGTATTTGAAG 112537
112538 ATTCTTTAACAATAAATGACCCACTTTTTGGCGGCCAAGTGGGGGGGGGGGGTGCGCGCACCCTGCGCA 112607
112608 TCCCCCCCCTGTGTACGCGCCTGTCAATGATTGTAAATATCGCCAAATCGTCAACTACATATGTTGTTTT 112677
112678 GAGTACAATTCAATTCAATCGTTCAATCGCGGAAACAAGCCGAGCGATTTTGAATGTAAAGCACTAGTAC 112747
112748 AGGCCGGACCGGATTGCGAGTACAAGCCGATTCTATCACAATACTAATCGATCGATCCCGAGTACAAGCC 112817
112818 GGACGACCCGAGTACAAGCCGAACGATTCGGGTACAAGCCGGACGATCGCGAGTAAAAAACGATCGTTCG 112887
112888 CGAGACAAGCCGAACCATCACAATTGTCACACGAGCAATCGCGCGTACAATGCAGATGAGTGCAAGCCGA 112957
112958 ACTGGGTGCAAGACAAGCTTGCTTTTACAATAGATTTGGCGTTTTTAATTTAGAAATAATAATGTTGTAG 113027
113028 CGGTCGAGGGCAAGAATGCCCCTAGGACCGACCCTAATCCTAAACTCTGCCGTCGCCATTGGAACAGTCA 113097
113098 CTTACGGAGTTCGTGTTTATTTAACAAATTGACCCCTCAACCGGCCTGTACCGGCTTTGGGAAGTACCCA 113167
113168 CAACCCAAAAAATTCATAAATGCAAACTAAACACAACAAGATGAAGATACTCAGGCATCAGTCTAAGGGA 113237
113238 TAAATGGTCTTGAAGAATGCATCCAACCATGGTGTTGGCAACTACTCTTAAGCCAAATAATAGCACAGGT 113307
113308 TCTTTGACAAATTTCAAATCGAACAAGTGAAGAAAATCAAAATACCACACAAGGGAGAGAGATCAGCAAA 113377
113378 CACAGCTCAAGACACAAATAGATACCTTTATTCTTATCCTCCAGGTACATTTCCTTGGCCTCAAAACACA 113447
113448 ATGTCCATTCCTGAAGGCTTGTAAAACTACGAAGATGCCTTTACGGATTGAAAAGCATTCTGGGAGATCC 113517
113518 AGGGAAAAAATTCACGAGGGGAGGGCCAGTCAACACTCCTCTCCCCAAAGTCAGATGGTTTTTTATAAGTC 113587
113588 TTTTCACCCCGAAGCCAAACAAATCAATTCCAGGTCATACAGACCCAGAAGAGTAGTGTAAACAATTTTG 113657
113658 GAATCAATTTGGTTTCAGAAAAGACAGCATTTAGGAGAGCTGTGGTGATTCATTAGAAAATTATTTTCTC 113727

113728 ATCCAGGCAAAGCCAAACAAGAAAAAATCATCATGAATCCACCTTTGCTTGCAAATATCCATAAGCAAAG 113797
113798 CACTCAATTATGCCCCAAAAGACTTCATGATGTCCTGAGACACTCTCCTAATATGCTCGTAGCTGTATTT 113867
113868 TTGATGAAAAATACAAGCAGCCATCAATTTGTGCTCGCTTCAGCACAACGACGAGGGATTAAAAGTGGGG 113937
113938 ACCGCAAAGCACTGTTGGAAAGCTTGGATACCGCTGACTAGGTGCAGCAGTGTTTGACTTTGACGGGCTG 114007
114008 TATAAAACTCAGAATAGGGGGGAGGTATCTGTTTTCAGTCTGTTTTTTTGTAAATTCAGCTCAAAAATAG 114077
114078 CCAGGAGTCAATGGGTTAATTGCCTCAACGTCAGCGTGCTTTTGACAAGAAGTGCAGGGTTGGTTAGTGC 114147
114148 GTTGGACATTTATTGTTGTAGATTATATTTGAGTTATAGATTTAGATCTCAATTGTATTAAATAACTTGT 114217
114218 GATGGTTTTCTTTTTCAATAATGATGCAAACCGGTTCTCATTTTGTTCAGAATTAGTGTGACAAATCCAT 114287
114288 GGCTTAGACTTCTTCAATTAGACAAATATTCTGTTTTCTCATGCCTCGAATATGCACTTGCAAAGCGAGG 114357
114358 GAAATGGAAGAAATCGCCTTCCATCTGTTTAGAAGTATTTACTACGCCTTACTTCCAAATCTTAGTTTAA 114427
114428 CAATTGTCAGAAAATTAGCAATATTATTTTGCAGACAATTTTTATCGCAAGAAGCTATTTGGTTTGTCA 114497
114498 ATGAACAACTTCTTGAAATAAAAACGCCAGGGGATTTTGAATATTAGAAGACATATTCTTTAAAACATAA 114567
114568 AGATACATGTATCACTCTTGTAGAAAGAAAAGCATTATCTTAGTTCCTTTTTTTTTTTAAATATTTTTTT 114637
114638 TCTTTTTCAAAGAACTTCACGTCACCTATATATACAAAGTATAATTTTTCCTTCGACAAAAGAATATCTG 114707
114708 GATTTTGTAGTTTAGTTGTGACTCAAACGTCGATACTGATATATTTTGAAATATTTCTGAATAATTCTAA 114777
114778 AATATCTACCAGTTGTATGAATTACTTACAAGCTAACTTGTTTTCTAAAGGAATTCAGAAAAAAACATGA 114847
114848 AAATATATAATTCAATCACTCACTTTCCTTTTGCTTTTAGAAGAAATTTCAAGAAGAGACAAAAGACATA 114917
114918 TATATAGACAAATAAAAAGTTTATAAGGAAAGTGTAAACACTAAAAAGGACATCCTTATATGCGGCTTTT 114987
114988 AATTTTCTACATGATCCATTTATCTTTTGCTTCCAACTGTTATTTATGTTCGAGCAGCATAGCAGGGCAC 115057
115058 CAAAAAAGATTAAGATTTTCTATAATGCATATCTTTTTTTTAAATATGGTCACATCTTTGCACCGATAAT 115127
115128 CATAAACGCAGTGGACAGCTTCGCCTACAGATCATTCCGTCCGCGTGTTTTATTCAAATAAATCAATTTT 115197
115198 GTTTACCCGTTGGAGATGTATGTATGCGCAGTGAGTCTTCGCCTGCTTTTTTATTCGAAACAAGGAATTT 115267
115268 ATACCATCCTTTCAATTGATTTTACAAGTATATGTTAAAAAAGTTATCAATTGATTTTACAAGTATATGT 115337
115338 TAAAAAAGTTAAAAGAACATTTGAAGTGAATTAATTATTATACACCTAATTAAAAAAAAGAGCGTTATCA 115407
115408 TAGTGTAAACGGCGAGATCCGAATGGCAGTTACAAGACCGAACGGTGCTTATGAACACCAGTGGTGCCAG 115477
115478 AGCCTTCGATAGCCTTTATAATTGATTATTGTATGATTGATTGATCCATGTTTTCCTGCATTCGGTTGAAAAAA 115547
115548 AAATTCGACACATGGTATCCTTTGGTTGTGGAACTGCCATATGCCAATCGCCATATATCGTTTGCACTGA 115617
115618 CAACGTTGCGGTTCTTGAAAGAAGTAGTTTTCAAAATTTTTGTGGATTTTATCAATGAGTTTTCTCTTTG 115687
115688 TTCCCCTTTTTTGTTTTCTTTTTATTTTTATTTGGTATGTTTTTGTAGAATAAGCCTGTTTTCCAGTCAT 115757
115758 CAGATATTTTCGCTATTTGCATAGTTCATGTGCAAATTCTGCAACAAAACGGACGCTTTATATTAGATTG 115827
115828 GTTTCTTTAATTCAAAACGCACAAATACACAAGGTTAGCACATTAAACGAGATAATTATCTAGCTTCTTT 115897
115898 TACAACAAGTGCTATTCTTTCTAACCTTCTTCAACGATTAGATAACCTTAGCTAATATAGTTTAGGTGAA 115967
115968 AGCTTCAAAACAAATTTATTATTTTTTTTTTTCAAATCATAACCTATAGATTCAATTTTATTTTAGGCCG 116037
116038 GGTTTGCTCTATGTTTGTGGCCAAATTGTCTAGAAATCACAAGATGCGGTACTTGAGAAAGCACCGCTTG 116107
116108 TTTTGAAGGTTTGATTTTGAGATAAAAAAAAAAACGCAAAAATGCGTTAATTATTTGTCGCCTGGTTACTT 116177
116178 CATCAAATCTAACAGCCGCGAAATCCCTTGATCGCGGCCTAATGTTCGCACGCTTCTATAGTTCACTCGA 116247
116248 GACCTATTTATCTTTATATCTTTTAAAGCAGCAGACCATATCCATCGCCATTTTATTTCTTGCTCTTGTG 116317
116318 TTTCACTCCAATTCTCTTTTTTTATCCAAGGGAAGATGTACGCCCTCTCCCCTCCCTCCCACCGCCCTAA 116387
116388 ATTGCAGATCGTCAACGATAATATTTCACGGTTATCTGGTTATTACACTCTCTCTATCTTCAGGTCCGAT 116457
116458 GGTCAAATAAAATAAAAACAGCAAAGAGCTTAACTTTCTCAAAAAATGTACCTGTGTTTACCGATTAACT 116527
116528 TACGAACTTAGTGGGTTTTTTTTTTATCCGCCAGCAAATTTGCTTCCCCGGCGACTTTCTTTGAGCCCAA 116597
116598 TTTTTTTATGGAGAACAAGCGACATTGATTCCTAATGGCCTTGCATTGTACTAAATACCGTCTTTAGTA 116667
116668 ACGGCATTATAAAAAACATGGTCCCTAAATGGTTCAAAAGACTGACAAATACTCCGAAAAAGAAGTCATG 116737
116738 CTGTTGCGTCCTCTCAAACTAGAGTAAATGATGGAAAATTCGAAATCCCACATTTAAGCAGTTGGGTTAC 116807
116808 AAAACAGGACTCACTCTATAATCCAAATTAACAAACGCAGAAGCCGATCCTTTGATAAATTTGACATTGA 116877
116878 CAAAACACAGTTGGGAATGTTTTCCTTTTCGGAATAACTAGCATTTTTCCCTTCCCCTTAGGCATTTTTA 116947
116948 CTTAACAAACTTATCGGCTAAGAACATGCAACGAACATTAGAGAATTAATCAACTTTTGGACTTTTCAAG 117017
117018 AATGCTAATTTTGCGTCCAATTGCAAGGCTCAGATATGAATTGAGAAGTATATTAGAGTGCATATACTTC 117087
117088 TTGAACCCTTTAATACTTGAAAAATTGCCAGAAAAAAAAAATGAATGTAGAAGAAGGTTTTGTAACAAAT 117157
117158 TAGTTCTAAATCGTGGGCGAACAGATGATAAGGTCGTTTCTTCGGCTTTTCTTTTTCCAATTAAAGATCG 117227
117228 ATCAAAGACAATTTTTTCTTATACGGGCGGGCTTTCTCATGTGATGAGTGGTGGTGTATAACCCTCTTCT 117297
117298 ATTTATTTGTGTAGCTATTGTCGAAGACACTATCCACAGTCAAGTATTGTTTGGACAATGATTAGATGTA 117367
117368 AAACAAAGATTGCTGATATATTGCCCGCGAACCATTGTTCTCCACGTTTAGCAAATAGGTTTAAACATAT 117437
117438 TCAGCAAGAGCTTAGGCTATAGTATAAGTAAGGCTATAGACCAGGGAATTAATCTCAAAGACTCTTAAAA 117507
117508 GCTAAAATAACAAAACAACATAAAATCCCATAAGTGAGAAAATGATCCTTGTACGTAAAGACACCTGACA 117577
117578 CAGAAATGAATCAAGCTTTGTATAGACAAACTAGGTTTTATAAGGCCAATTAAAGTTCGCTCACATCAGA 117647
117648 AAGAATCGCCTGTGACGATGTTTGCTTATTTCCCGCGGGTTTATGCACTCGGCTGTTTGCAACCGGTTTT 117717
117718 TGATGTACCTTGAGAACAAAAATACACAAAGCATTTTCATATCACACACTTGTTGGCATAAATGATATCG 117787
117788 CACTTTTTTCTTAGTAGCAATTCAGGTAACCTTTTATAAAACACGGAAGGCAGCCGTTTTTGAATCATCA 117857
117858 GTTCTTTGTTGTTTGTTGGGATGAGCCTTATTTATGTGTACGCAGTTGTAGTTTATAATAAGTTGCAGAT 117927
117928 GAAAAAAAACAAATTGTTCAAGTTTTTAGTTTTAATTCCGAATCTAATTGCGTACCACTTATCCAAATTA 117997
117998 TCCGAGTAATGGCTTGAGAATACAGAAAATGAGAAATTGAAAATGCAGATGGGAATTTGAAAATAAGCCT 118067
118068 TCTGTTTGGTAAGAAAAGCGGCAATCGATTAGCTACTAAGGAAAAACACCGCCCGTGGCTTGCAATATTT 118137
118138 CCAAAACTATATTTTCAAATTTGTACTATTCGGTATCTGGGAATTGCTTCCTCTTGACAAGCAAGACCGA 118207
118208 TATTAAACATCTTCTTAATATCATATATATATATATATATAATATCATATTACTTACACTCGTTGAGA 118277
118278 AGTAACATTTAACAAAAAAAAAACGTTTAGAAATCCGTTAAAATCAATTCGTACTCTCGGTACAGCATAA 118347
118348 TTTAACAAAATCCAAGCCAAGGGCAGGCTGTTCTCTTCCCTAAAAGTTGAAAAAAACCATTTTCACACCA 118417
118418 AACAACAATTGGTAAAATACTCAAGCCGCTTCGTTTCATTCTAAAGAATAGCCTGAATAATTGTGAAACA 118487
118488 GTCGGAACGATCAATGGCAATTATATTACCTAGTTGTTTATTTGCGAGTGGACTGTCTTTATTAACTTTA 118557
118558 CTAACTGGTTATGGAAGACTGGAACTCGGCGGTCGGCTTTCTGGACCAGTAGGCATAGATAAACAAAGTT 118627
118628 TAACTGAACCTCTAAGATACTAAGATAAAAAAATAGCATGATTCTTTACGTAAAAATGCGTTTACGAATA 118697
118698 AAGCGTTTAACTGTTAAATGGGAATCTGTTAGGTGGAATTTTTAGAATCTTTTGTTCAAAACAATGCGTA 118767
118768 TGGGTGAGACTTTGCAGGCTAATTATCTACCTTGAGTTTATTTATGCGCCTGTTATTATGTTCATATCTT 118837

118838 CCCCAAAATGAGCAAAGTGTGAGATTTTGAAAGTGAAGAAACAAAAACACAAACGCTATGATCTTTGTTG 118907
118908 AAGTTTGAAGTTTAGGCCTAGCGGGGAGTATTCGTGGATGGCGATCAGTTCAAATTTACTTTGCTATTTC 118977
118978 ATTCCCCAGGCTTTTCCAGAGTTTCGAAATGAACTAATTTGGTTGCCAAGCTTCGTTGAACGGTGTAGTT 119047
119048 AATGTGTCTTAATTAAAAAAGCATTTTACATTCGCCAGGTGTCCCTGTGAGTAAATTACGCCGAGAGGCA 119117
119118 GCACTAAGCGTGCGCGTAACATTCAGAGTACCGTGAAAAAAGATAAGTTATATCTCTATACCACAAGTGG 119187
119188 ACGATATCACACCGGTACATGTATCACTACGAAATGATTTAAACTACACCACACTCTCTTTTAGATATAC 119257
119258 AAGATATAGAAGAAATACATTAGTATAATTTTCACTCTGTGATGTCCCCCCTTCTCCAGATAGGGGGGGA 119327
119328 GCTTGTATTAAAGTATTACCCGCATCACGTGATCTCATTGCAGCCACTCAGAGCGCGCGCTTTCCGTATA 119397
119398 ATGTAATTGACAAAAGGTATTGTGTGCGACCTTTAACAAACCAGAAACCGTTCGCTCACGTAACTATAAC 119467
119468 ATATCAGGTGGTGAGTCTCAGAGCTCACATTAATCTGGAGCTTGCACCCGTTCACTTAACTCATTGAAAA 119537
119538 CCCACAGCACACAGAAACCCAAGGTAAGAATAGGCTTTAAATCAATCTTGCGAAATCTCTCGCACTTATA 119607
119608 TTTACACGTTACAAAAGGCTTTGCACACGTCTTGGCTATTGAATGTTGCTGGATTATTAACAAATTGGCT 119677
119678 TTTACTCATTCTGCGGTTTGCGTAATCTGTTAAAGAGTGCTCCACACCAGAAATTGACTCAAATATTTGT 119747
119748 TCAGAGAGAATTGCGGTGCAGATCTGGCCAAGTAACATTATGGCATTAAGTCGTTGGTGTCTGGATGCAG 119817
119818 TGGAGTTATCAAATATATATTTGTACAAGAATTATCTCTTTTCAAACTCTATCAATTCGCGTGGAACTTG 119887
119888 CTGTAGCTTATTGAATACTTTATTTTATTACTTTTTGACTGTCGGACCTTAACGATTGTATAAATAACAT 119957
119958 AAAACGGACGCATTTTTCAGTTGATATCCAAATGTATTGTTTGTTATTCAAGTAAATTAAAAATACCCCC 120027
120028 AAAAACGCCAAACACATAAAACTAGTACAACATCTAGTGTTTAGTGCAACTAAAATTTGATTGATAAAAT 120097
120098 GTTATTTCTTCCGAAAAATATGAGTTAAAACAACGTTCGTGACTAACGATATTTTCCCAGATTATCAATT 120167
120168 TAACCTCTCGTTGTACTGTATTGTATAAAGTTACCCATAACTGTAATATCATCTGAGTACATACTCGTAG 120237
120238 CGTACATCACATCTATTTATAAACCAGATAAACAACGAATACAACCAAGGAAAAAAATATCCTCAAATAC 120307
120308 TGACGAATAAAACTTCTCATACGCATATAACTTCATAAACTAAAACAAACTTTCACAAGTTTAATAATTA 120377
120378 ATTATTAACCAGCTTGAATAGGCGTAAATGCCGAATGTAAAGGGAGAGAAGTAATAGAATGAGTGGAGAT 120447
120448 TGCTTAAAGGCGAAAAGTTTGCTAAGTACAGTACTACAGTTTTACTAGGAAACACAACGTAGGAAGACGC 120517
120518 TATTGCCGCTTGTGCAAGATCTCCACTCTTTGCCCTCATTTCCGCCAATACCTCTAATTATCTACCGACG 120587
120588 TTGCTAACACATCTAACAGCGCTCAGAGTGAAAGTAATAACTTAAAATTTCCATTTTGTTTTTCTTCTCT 120657
120658 ATTACCGATACGTCGTTTTACGGTACTTGATTAAATGGCCGCTTTACCTCGGGTGCTGTGTTAAGAACCT 120727
120728 CAAAACTCGATTAAATCTTCAGGATATACCCTGATAGTTACACTATAACTATGTAAGATGTAATAAAAAC 120797
120798 ATTTATGTGCCCCGGAGAGGTCATTGATTTTAATCTAGGTGCGTGAGCACTTATGTATCTACGTCGGCGG 120867
120868 AAAACAGAAATCCGCCCTTTTCGTACCTAAACGTACATCCTTCCGATCGTTAAACCGTCCAGAAACGAAA 120937
120938 AAATAACATAAGTAGAAACAGTAGTACATAGTACACTACGACTAAGGAGTCGAAAGGAAGAATATTTAAC 121007
121008 TTTAAACTTAGAAAAGCTTTGACTTACACTATGAGCTAAGATTAAAACTATGCGTCAAAAAGCCAGGAAA 121077
121078 GAAGGTGAAAAAGTTCCTTTATAAACAAATTTCATTTCTCTTTAGGCGCTTTCATATTTCCGATATTTTT 121147
121148 CCAATTTGCAAACTATTAATATCGCTAAGATTTGTGTTACGATGTTGACCTAGACGACAGAAACCTCTAG 121217
121218 AATACGATCATTTGAGCAGATATGTCACATATATGTAGGTCAACATCCTATTACGTCAGAGTACACCAAA 121287
121288 TTTCATCGGCAAGTCTAAGTAAACAAGTTTACCTGTTCTATGGCAACTTAAGAGCTTAAGGTTTACCTTT 121357
121358 CGTTTGATACTGGTAGGTGGTTTATTAAAAAGCGACAAAAGTCAAGCTTCTAGCGGTTAAACAATCATCA 121427
121428 GTTTTGCTGAATGTAATAAGCATTACAGTAGCACACTAGGAGTCCCACTATGAACTATATCTACCGGTCT 121497
121498 AGAATCCCGTATTCATGACGCGAGATAATGGAAAGTCCAAGGGAATAGTAGACTAGCGACCGGTGCCAAC 121567
121568 ATCATGGTATTGTTTGGGAACGTTGTTACCTAGATTATTCTAGCCCTCGCCTTTAATTATTTTGATTTTC 121637
121638 GGTAACCTATACCATCCTAAATGCACTATTAAAAGGTTTTTTGGCAATTTGTGGAAACATCTGCGCCTTC 121707
121708 TTGATTGTCTGTTAATAATAAACTGTAGAGGCAAACACGAGAAGAGAAGCTAAGGGAGCAAGTGAGGCTA 121777
121778 AAAGGTGTAGTATCTGGATTTCTGAGAGAACTCGTTGTCTTTTTTTTGCAAGAAAAAAATAACAGCACTG 121847
121848 TTAAAAATATAAAAGGGCCTACGTCTGTCAATTCCTTCTGAATTGATTTTCAATTACATAGGATCAAAAT 121917
121918 CACAATATAAGATAAGTAAGTATTTATGTAATGTTTCAGATTGAGTTTTCTGCTCGGTCCGTATTCATT 121987
121988 ATCGTCCAAAAACGCCCCCCCCCCCCCCCCCTTCAAAAGTCAAATGGCCGGGCCCTAATGGTACTGTACA 122057
122058 GGATGAATACGTTTCGTGTTGCGTGCAAATAAACTTAGCTTGACATTCGTAACAAAACACCTCAAATGTC 122127
122128 TACGAGTAAATGAATTCCACGTGCTGAAAGACGCTATGACAGACAACACTTCTGTCTACTGGTGTTGTAA 122197
122198 ATCAAAATAAAATTAATGAGTAGGAGTGGGAAAAGCAGAGTCTTAAGCGAAGAAATCATTTATCTAGAAC 122267
122268 TCGCGGGATATTGTGTTAGTTTGTTCGTTGCATTATGTTTGTAATGGTTGTACTGTTTATACTGCCAATG 122337
122338 TGTTCATGTGACGTCAACAGTGTACTTGTACTGCGGAGAGGGGTCAAGATCTTGCCATCACGTATTCGGG 122407
122408 AAAAATAACATCTTATTTCTCGTTTGCTGCTTCCGCCCAGCTCCAACACACGAAAATAGTAGTGTTCTTC 122477
122478 TCGGTATTATGACTTGTGACTGAATGCGAAATCTCCCAAATCACGAGCGTTTCTGTGACAAACGACCAAA 122547
122548 TAACACACAAGGTAATTGATAGTTTAGGTGCGGCTGCCTCAGAGTTGTTTACGCATTAGTTGCTCCCTAC 122617
122618 AGCACGACTATCCTACGGTAGCCGCTGTTCGGATTTCAGTACGATTCTATTAACCGCGGGCTCAAATGCG 122687
122688 TTCCAAGTCGTATAAAACGTGTACGCTAAAAACGAGAACGTGAATACGCAATTAAGTGCTCTGTGATAAA 122757
122758 GCCGCGCGTTTGAACTGTTTATTTTCCCCTTTCCGTTACAGAGCTCTTAAAGAGCCCTTAAAGAGTTCTT 122827
122828 TTTTAAGGAAATAAATAGAGATAATTGTATGCTCTGTTTAGCCTAAAGCAGACAGATTATTACAGACCCA 122897
122898 TAGCATTACAAGTGAGCAGCATGATAGATTAATCAGTCTATCCTGCCAATTTAAAAGTCTATCCCAATTA 122967
122968 AACAGTCTATCGGCAACTGTGTTGCCGGAGGTTGTTTTCAGCTCCGATTCATCCTTTTTAATGGTGTTTT 123037
123038 CCCCGTAGCTTAATGAGGGAAGAATAGCCGTTGTCACGATACTTTACTTTACGGCTCCCGTGTTCTATTT 123107
123108 TCGGTTTCACAAACCCACCGATTTTATGAAATAAAATCCAACTCCTATCACTTCGATTTTGTATTCGAGA 123177
123178 AAAAATAACACCATAAACTGATAATATCGCCGAGTTCCAATTTTGAACGCTATTTTTTTTATAAGAAAGT 123247
123248 TTAAAATGGCTAAACAGCCTTTATTATATTCATTATATGCACATTAGACAAACTCGATGGCATTTTTTTC 123317
123318 ACCCCTTCAGCAGTTAGCATTGATTTCTAAAAGCGAATAATGTAGACCTAATTACACTTCGGAGCATGAA 123387
123388 TAAAAATATTTCCCCGTCGACATTGTAATGTTTTGATGGACCAAATGGGCTGTTCTCACGACGTTTTGTT 123457
123458 TATCCGAAAGCTCTACAGAAATGCAATTGTGCACCTATTACTTTCAACATTGCTTGCTGTGTATCATCAG 123527
123528 TGGAGTAAAATAGAACCCAAACCATTGAGCAATAATAAACTCATTGAGCCCAAATCATTCAGGAATTATC 123597
123598 GCTAATCACCATATAATGTTTGCACGTTTTTTTATAGAATTGCTAAGCACTGTAAAAAAAAATATTTGAT 123667
123668 AGCGAAGGTAATTTTGTAGGCATTACTAATTGACGTATAAGACTGTTAGTGTTGCCAAGTGTTCCAAGGG 123737
123738 TCCGGTTTATGGTAATATTTTTCCTTTTTAACCATGCTTCTCAGAGCCTAATGACATAACGGCACTACCC 123807
123808 TTTGGTTCAACCTTCAGAAGTTGCTCAGATTAAGTTATTTTGACGCAGATTCGCAAGTATACAGCCCATA 123877
123878 TTAATAGTTTTCGTCTATTTTCTCTCGAACTTTTGTAACAAGGTAGAACTTGCTATAACAAATATGTCTT 123947

123948 GAATCTTTGTTACTTCTTTGGTTGAGGATAAAATCGTCGAATAACAAGTCAACTAAACTGGCTTATAAAT 124017
124018 TCTAAGTTTAGGTTAATGATGTTTTGTGTTTGTGTTTAGGATGAGCGATACCCTGACGAGTGGCCCCGTA 124087
124088 CCCGGATCTGCCTTGTACCATCGCATCCCCAAGTGCGCGCGTTGCCGGAATCACGGCGCGCTCTCGTGGC 124157
124158 TCAAAGGACACAAACATTACTGCAGATGGCGGGACTGCACTTGCTCCAAATGCTTACTGATCGCTGAGCG 124227
124228 ACAACGGATCACTGCAGCGAGAGTTGCCCTGCTTCGACAACAGCGAAAGAACGCCACTGGCGATGGGGAC 124297
124298 AAGAAGGATATATTGAGAAAGACCACGGAGAAAACGGAAGAGCTGCGAGAGAAAAGATTCAGTGAAGAGG 124367
124368 CGTTTGTCCGGCCTTCCATGGTGCTCAACCCGCTTGGATCTCTACACAGCCCGACCAGCAGCGAAGGTAG 124437
124438 GAGCACTTAAAGACTAAGCTAGAATTGTAAAAAACGGATATTTTTTTCGTTGTGAAAAAGTCGCAAAAAC 124507
124508 TAGGCTGATCGTAAGACAAAATTATAGTAGTCACTATACCTAGGGGCGGATACCTGGGGGGGGGGGGGAG 124577
124578 GGGGGATTGGGTGGATATGCATACCCCCTTTTTGAGCAAAACAAATTGAAAAGCCACTTTGTTTGAAGAA 124647
124648 ACTACGGAGAAGATACTGTCCGTGTGCCTGTGTACCATCGCCTGCTTGACTTTGCTGACGCAACAAATCT 124717
124718 TATTCAGTGTGAAGTATTGTTAGATCCATGTGACCTACCAAGAACCACTGGATCAGTTGTTAGCCACCCA 124787
124788 TCCGACCATTATCCACCCCCTATTTTGAAATCCTAGATCCGCCCCTAATACGTTCCTTGATACTGAAATA 124857
124858 TAAGTAGTTAATATGTCTTCTGGTCAAACCCGATGTTTTATTGAGTGATAACCGTCGATTTTCTTAATAG 124927
124928 AGAACGACGAAGGAGGATACCAGCACGCCTACCATCAAGAGTCGGATACAGAGACAGACCTGAGCGAAGC 124997
124998 AGAAGGTCTACACCATAAACCCGAGGCTATCATACCTATAGTCAGCGGTATTCAAATCAAAACCGAGCCG 125067
125068 AAGTGCAACGACGAGGAATGTAACCCTACAGAACACAGGAGTCTCGCTGCTTCCCCAAGGCAAACACCTT 125137
125138 CCCCGAAACGCTCGATCGCCGAAGACAGCGACGAGCGTCAAAATGAGGAGCCACCAACAAAAGCTCTGAA 125207
125208 GAGAACAGACAGTCCAAATGGAGCCGTGCCCAACCGCAAGCTACACCCCGATCCTCTAGGCCTCCTTTTG 125277
125278 CGAGCATTTCCTGGACATTGTAAAAGCGTACTGGAACTGGTACTACAAGGGTGTGGCGGGAATGTAGTCC 125347
125348 AGGCAATCGAATGCTTGCTTAACAGCCAGGAAAAGAAGTCGCAAGGACTTCAACTGCCTCTGCCGATATT 125417
125418 GACTGGCTACCCAACTTTACCACGTGATCTTCATCCAGGTATTCACGGACCTCCTCTATTCCGTAAACCG 125487
125488 GAGCTTTTCCAGCACCCGTTCCAAAGAGTCATTCCCATCGAGTGTAGGTACCCACCACCGCCTCTACTCA 125557
125558 GGCCAAAACCGCAGACCACTACATACGGCGGATTCACTTCAGAAGCACGCGGAATACCGGAGCCCATTGT 125627
125628 GACTACGGGCTCAGCAATGAACGATAGAGTGTCACCGGGTGCTCGCTTTTGCACCAAATGTGGCAAAAAG 125697
125698 GCGGAGGTGTCTGATAACTTTTGCGCCCTATGTGGCCAGAAATTAAGCAAATAGATATACCACATTCATT 125767
125768 CAGCAAGTTGTGGCCAGCTTTAAAGACTATAAAAGCTATTTATAAAGATGTCGTATATTTCCTATTTATG 125837
125838 GAAGAACAATTTGGACGCAGACAAGTTACGAGGCCAGTAATATGGCGGACAGAAGCTAGAGTAGAAAATG 125907
125908 TTAAAAGCTGTGCTGACAATGTTCGAAAGGAGGTGTCCGAAGGAGGCGTAAAGGATAATCGCAATTTAGC 125977
125978 TGAATCTTTGGGTCTCATGTACATTCTCTCAATTCATATTATGACAAGCAAAACCCACAAGGAAGATAAT 126047
126048 ATTCTGCTCATATCCCCCTCGAATTTATTTCTCTAGCTAGGAAACAGGTTTCGAAAAGTTTTAAAGATGA 126117
126118 CAAATACAGAAAACTTCATATAAGTGTTTTTATAGTATTCAGACGTTCATCCAGTCGCTTTTAGATGTTT 126187
126188 TCCAGTAGGTAATTCATGCAAAATAGGTTTTGGTGTAGGATTGTTGTCGTTCAATATTGAATTAAAGTTC 126257
126258 TCAACAACACTTCAATGGGAATTCATTGGGAGACAACGAGAGGGGAGGGATGGTGAGTTAGCGAGAGGGG 126327
126328 AGGGAAGAGGATTCAGCGAGAGGGGATTACTGAGGAGTAAGCGAGAGGGAAGGGATAAGGAGTTAGCGAG 126397
126398 AAGGGAGGAAGGAGGAGTTGTTAAAGGAAGGGATGGGGAGGGATGGGGAATTCAGGACCTGTCACACGTG 126467
126468 GACTTTCGGCTGCAACTTGTCTCGCAAAAAAAATGTTGCAGGTCGCACGTCGTGCGACTTTGAGAAATGC 126537
126538 AAAACAAGTTGCAAAGGGGGTGTCACACGCAAACAAAATGCACGTGCGACCTGCAACAATTTTTTTGGCG 126607
126608 AGACAGGTCGCAAGATAAAAAACGTACGTGTGACATGGCCTTTAGCGCGAGGGAGGGATGGGTAGTTAGC 126677
126678 GCGAGGGAGGGATGGGTAGTTAGCGCGAGGGAGGGATGGGTAGTTAGCGCGAGGGAGGGATGGGTAGTTA 126747
126748 GCGCGAGGGGTGGGAGATAGGGACTCGATTAACAGCGGGCTCAAATGCGAGTCAGGTCGCGGTGAATAGA 126817
126818 GAAAACCATAATATAAAACGTGTTCGAGAAAAACGCGAACGTGAACACGCTATTAATTGCTCTGTGAAAG 126887
126888 CCAAGCAATTAAACTATTTATATTTTCCCATTACCGTTATAACGCTCTTAAAGTTGCTTACAGTGTATCA 126957
126958 TTAGTAAAAAAAATAAGCCGAACCATTAAATTGAGAAATCATAAAGTCATTGATCCCAAATCATTCAGGA 127027
127028 ATTATCGCTAATCGCCATATAATGTTTGCATGTTTTTTTATAGAATAGCTAAGCACTGTAAAAATAATCT 127097
127098 CTTTGATTATAACGAAGCTAATTTTGTAGGCATTACTAATTGACGTATAAGACTGTTTTGCCAAGTGTTC 127167
127168 CAAGGGTCCGGTTTATGGTAATATTTTTCCTTTTTAACCATGCTTCTCAGAGCTTAATGACATAACGGCA 127237
127238 CTACCCTTTGGTTCAACCTTCATAAGTTACTCAGATTAAATAATTTTGACGCAGGTTCACAAGTTTACAG 127307
127308 CCCATATTAATATTTTCGTCTATTTTCTCTCGAACTTTTTTTGTACCAAGGTAGCTCAAGGTCGAACTTT 127377
127378 CCATAACAAATATGTCTTTAATCTTTGTTACTTCTTTGGTTGTCGAAGATAAAATCGGTGACGGACAAGT 127447
127448 CAACTAAACTGGCTTTTTAAGGTTAAGTTTAGGTTAATGATGTTTTATGTTTCTGTTTAGGATGAGCGAT 127517
127518 ACCCTAACGAGTGGCCCCGTACCCGGATCTGCCTTGTACCATCGCATCCCCAAGTGCGCGCGTTGTCGAA 127587
127588 ATCACGGCGCGCTCTCGTGGCTCAAAGGACACAAACATTACTGCAACATTACATCGAGCTTTGGAGTTGA 127657
127658 GCACTTGTGGCTTGTAAATCCAGAAAAGATTCCTTCTTTCACTTCAGCAGCATTAGTTTTCCTTCTCGTA 127727
127728 CAAATATTTAAAAAAGCCATATAATAAATAACTTATTAACCTCGCTTGCTCGGTCCGTACTGAGAAGNNN 127797
127798 NNNNNNNNNNNNNNNNNNNNNNNNNNNNNNNNNNNNNNNNNNNNNNNNNNNNNNNNNNNNNNNNNNNNNN 127867
127868 NNNNNNNNNNNNNNNNNNNNNNNNNNNNNNNNNNNNNNNNNNNNNNNNNNNNNNNNNNNNNNNNNNNNNN 127937
127938 NNNNNNNNNNNNNNNNNNNNNNNNNNNNNNNNNNNNNNNNNNNNNNNNNNNNNNNNNNNNNNNNNNNNNN 128007
128008 NNNNNNNNNNNNNNNNNNNNNNNNNNNNNNNNNNNNNNNNNNNNNNNNNNNNNNNNNNNNNNNNNNNNNN 128077
128078 NNNNNNNNNNNNNNNNNNNNNNNNNNNNNNNNNNNNNNNNNNNNNNNNNNNNNNNNNNNNNNNNNNNNNN 128147
128148 NNNNNNNNNNNNNNNNNNNNNNNNNNNNNNNNNNNNNNNNNNNNNNNNNNNNNNNNNNNNNNNNNNNNNN 128217
128218 NNNNNNNNNNNNNNNNNNNNNNNNNNNNNNNNNNNNNNNNNNNNNNNNNNNNNNNNNNNNNNNNNNNNNN 128287
128288 NNNNNNNNNNNNNNNNNNNNNNNNNNNNNNNNNNNNNNNNNNNNNNNNNNNNNNNNNNNNNNNNNNNNNN 128357
128358 NNNNNNNNNNNNNNNNNNNNNNNNNNNNNNNNNNNNNNNNNNNNNNNNNNNNNNNNNNNNNNNNNNNNNN 128427
128428 NNNNNNNNNNNNNNNNNNNNNNNNNNNNNNNACCTTTTACCATGCTTCTTAGAGCCTGATGACATAACGGA 128497
128498 ACGATTCTTTGGTTCGACCTAAAAAAAAGTTACTCAGATTAAGTTATATTGACGCAGATTTGCCGGATAC 128567
128568 AGCCCATATTAATAGTTTTCGTCTGATTTCTTTGAACGTTTGTACTAAGGTAAGACTTGCTATACAATAT 128637
128638 ATGTCTTAAAAGCTTTGTTACTACTTTAGTGTCGAAGATAAAAGCGGCGAGTGAGACAAGACAACTTAAC 128707
128708 TGGCTGATAGATTTGTTTTTTTTTTGGTTTATTATGTTTTATGTTTGTGTTTAGGATGAGCGATACCCTA 128777
128778 ACGAGTGGCCCCGTACCCGTATCTGCCTTGTATCATCGCAACCTCGAGTGCGCGCGTTGTCGAAATCACG 128847
128848 GCGCGCTCTCGTGGCTCAAAGGACACAAACATTACTGCAGATGGTGGGACTGCACTTGCTCCAAATGCTT 128917
128918 ACTGATCGTTGAGCGACAACGGATCACTGCAGCGAGAGTTGCCCTGCTTCGACAACAGCGAAAGAACGCC 128987
128988 ACTGGCGATGGGGGCAAGAAGGATATATTGAGAAAGACCACGGAGAAAACGGAAGAGCTGCGAAAGAAAA 129057

129058 GATTCCGTGAAGAGGCGTTTGTCCGGCCTTCCATGGTGCTCAACCCGCCCGCATCTCTACACAGCTAGAC 129127
129128 CAGCAGCGAAGGTAGGAGCACTTTGAGACTAGGCTAGAATTGTAAAACACTAAGACTTTTATCGTTGTGA 129197
129198 TAAAGTCACAAAATTCAGTCAGGAAAAACGTATAGGAGGATGGTCTTTTTCTTCGATCTTAGAGTAATAG 129267
129268 TTCTTATAGCATTAATTTCGAAAATTGTGTATTTGTAGTTTCAATTTACCTGTTTTTAATACTGAAATGT 129337
129338 AGATAGCTAATATCTCTTCTGATAAACATTGTTATTTTATCGAGTGATAACCGTCGATTTTCTTAATAGA 129407
129408 GAACGACGAAGGAGGATACCAGCACGCCTACCATCAAGAGTCGGATGCAGAAACAGACCTGAGCGAAGCA 129477
129478 GGTCTACACCATAAACCCGAGCCTATCATACCTAAAGTCAGCGGTATTCAAATCAAAACCGAGCCGAGGT 129547
129548 GCAACGACGAGGAATGTACCCCAACAGAACACAGGAGTCCCGCTGCTTCCCAAAGGCAAACACCTTCCCC 129617
129618 GAAACGCTCGATCGCCGAAGACAGCGACGAGCGTCAAAATGACGAGCCATCAACAAAAGCTCTGAAGAGA 129687
129688 ACAGACAGTCCAAATGAATCCGTCCCCAACCGCAAGCTACACCCCGATCCTCTAGGCCTCCTTTTGCGAG 129757
129758 CATTTCCTGGACATTGTAAAAGCGTACTGGAACTGGTACTACAAGGGTGTGGCGAAATTGATTCCAGGCA 129827
129828 ATCGAATGTTTGCTTAACAGCCAGGAAAAGAAGTCGCAAGGGCTTTAACACCCGTTCCAAAGAGTCATCC 129897
129898 CCATCAAGTGTAGGTACCCACCACCGCCTCCTCTCTGGCCAAAACCGCAGACCACTACATACGGCGGAAT 129967
129968 CACTTCAGAAGCACGCGGAATACCGGAGCCCATTGTGACTACGGGCTCAGCTATGAATGATAGTGTCACC 130037
130038 GGCTGCTCGCTTTTGCACCAAATGTGGAAAAAGGCGGTGTCTGATAACTTTTGCGCTCTATGTGGCCAAA 130107
130108 AATTAAGCAGAAAGATAGAACCATACATTGTGCAAGTTGTGGACAGCTTTAAAGACTATACTAGAGTGTC 130177
130178 TAATAGAAAGGAAATGCTGCGACAGTGA 130205

DMRT E

136538 AGAAAAATCGTCTCCAAGTCGTGTGCGAGGATATTTCGTCATATTGTTTGCCGCTATGGAAATTCGTTTT 136469
136468 GTCACCTTTTCCAGTAACACTTTTTTGACAATCGTCCTGAACAATGGAACGCGCTGAAAAGCCAGCATTC 136399
136398 CGACGGCCTCTCAAGGGAAATCTGCGATGATGTCATAACGAGTCACGTGGGTACACTACTACTTCCGAAA 136329
136328 GAGTCGGGGAAGGTCTCATTGAGAAGAGAGATCGCGTACTGTAATTGTGATGGTTGCAGATATGAAGGAA 136259
136258 GAAGCGTTAGATTCGCCCCTGGATAAAAACCATCTCTCAAATAGGACGCCAAAATGTACGCGCTGTAGAA 136189
136188 ATCACGGGATATTATCAGACCTTCGTGGCCACAAGCATCAGTGCCGGTTTAAGGACTGCGCGTGCAACGA 136119
136118 ATGCATGATTGTAGCTGAGAGGCAGAAGCTTACTGCCGCAAGAATAGCGCTGTACCGTCAGCAACGAATT 136049
136048 GACGAACCCGAAATGAGAATCAACGGGGAAGACGCTCACAGCCTAGTGATGCACCCAAACGGAATGTGGA 135979
135978 ATGGTGAAGCAGACAAATCTCTGGATTCGAGTAAGTTCATATCTTCCTTGTATTATCCGTCATTGAATAG 135909
135908 CCATTCTATGCCAAACATCGCTTTGTACTTAAGTCCTTAGAAGGAAGGCTTCTTTATCATCTTTTTCATT 135839
135838 CGTCTTTGAGACTTTCTTAACGTCTTACGGTCATTCAGTGATTTTCTCTTAGTCCCGTTAGAGAAAATAC 135769
135768 TTGTAAAATAGCATTACTAAGTTATTTGATTTTTTTAACCAAATAACCTTTGTCACTTATACTGTTGG 135699
135698 AATTATTGCTTAATTAATCAAGCAAGTTCTTCAACAAAAAGAATTCGAATGTAGGTCAGTAATTAGCGCT 135629
135628 CGATTTGGTACCATGCAAGTACATGTAAATTTGTTCGAACGTTAATTAAAGAGTCATCAGCTGCAAGTTC 135559
135558 ACGCCATCCTTCGCTGATTGTAACTAACTTGTACGGGTACCAAAAAAACAAACCATTTTGATCGCCTAAA 135489
135488 ACAACATGTTTCTCTCTTTTTTTTTTTCAAATGAATGCGTTTACCAAAAATCTACAAGCTTCTCACTTTT 135419
135418 AGAGACCTTTTTTCAAAGAATTCAAAGTCTCACGTCCAATGTTTGCTTTCTGCTTGTTGCAATTACAAAA 135349
135348 CTATAAGTATCACTTAAGCATTCTCTCCCGGTGCAATCAAAAACATATATAAAAGCTAAGCCTAGGCTAC 135279
135278 CAATAAACAGAAAGGAGTTTCCGATTGTCTAAGCTTGGCACGTGATGTTTGTGGCTTTCCGGAAATCCGC 135209
135208 ATCAGGTTCTATTTGGAAAACAACAAAAATATATCTCTTTATCCAGGAAATTGTTGGAAGCAAAGTAATC 135139
135138 CGGGTTTTGTTGAGCAAATTCCACTCTAACAGTTTGCTTTTCCTGTAGAATCAATTCAAGCTATCCATTC 135069
135068 AGGTTTTCGTTACATTGTGGTACTTTAGCGTCGGTTTCTTTTCCATGGTTTTGTTACCGAATTATTCATG 134999
134998 GGATAAGTCATTGTGCTACGACCTCTAATTTGCGGCTTGTTTTCTAACAATGCGGGACTGGCTTGCAATG 134929
134928 GGTTATCAATAGAAACAAAAGCCGCCTTTGAAAGAGAAAACACAGAAACACTGACTTATTTACAGCCACA 134859
134858 GACAAAAAGGCTAAATTACACAAGACACCATTTTGCCTCCGCCGCGTCAATTGAGGGATTTTAGCAAGTA 134789
134788 CGAAATAATTTAGATCATTTTTCTTAACAATTCCTTGAGACCTCTAAAAAAAAATTCTAGTTTTAGTCTG 134719
134718 TGACGCAACCCCAAAACTTTTTATTTCTGAACACTTTTGGTTATTTTTTTTTTTTAACATTAGGGTCTG 134649
134648 AACTCGACGGAGTTAATGGTCGAATTAACTTACTGTTGAATATAATGCATAAGTTATTTTAACAAAATGG 134579
134578 GAGGTGGATAATGGCCGGATAGACGGCTTATAACTGATCCAGTGGTTCTTGGTAGGTCACTACATAAATC 134509
134508 CTACATACTTCACACTGTATTTAATTTGTAGCGTCAGCAAAGTCATGCGAAGGCGCATGGACAGTAACTC 134439
134438 CCGTCCCTCAGACATATAATTAACTTCAAACGAAAAAAGGAGGTGGATTTCTACCCAATACACCCCCTGA 134369
134368 ATCCGCCCCTGGTACAGAACTTAGCATGAATTCGCATGAAAATCGCAAGAAATCGCCGAAAACCGAGACT 134299
134298 TGGCGACATTTTATTTAATGCGTTTTGAGGCATGCGCAAAGGGTTCTTGCCAACGGGTATCGTGACTCGT 134229
134228 TTGATTAAATTCATGATTTTCATCTCTTTTCTTCGTGGAGCACCATTTTGATAGCATTTTATTTTTCCTC 134159
134158 TAAGATTTTTGCGCTGAATGTAGAATTCTTTTCCAAATGAACAGATATTAAACGGGTAAAGCAAGCTTAA 134089
134088 GTGACTTGATTTTAGTTTTGGTCGTCAAGAAAAACCTGGCAAAGAAAAATAATTACTAATAAAACTTGCA 134019
134018 AAACCTGTTTTTATGGACTGAACCCTTCGTTCGTAACAAATGTTTTCGGTTTTCCCGTAATGTAATTTAG 133949
133948 TTTTTGGTGTCTATGAAAGTCTGGCATCACTTCAACGTACCTCAATTTAAAAAAATATTTCTAGGTATGT 133879
133878 TTTTAGTTCGAGAAAGTTTTTGTAAAATCAAATAATGCACTTCTATGTTATAATTTCGCAAACAATTGTA 133809
133808 ATTTCCAAGGCTGGATTTGCATCTTCTATTACAAAAAAACTCAAATTTGCTCAAGCAAGACTCCCTTTCA 133739
133738 AAAAAAGACATGAATGAAGCTTTTTTAAAATCATTATAGGAAACAACAGTTTTAATTGAAAGAATGTCGA 133669
133668 TAAACCATTCCTTTATTGTTTTCAGTCGAAAATGCATCGGTTTATTTTCAAGAAAACCTAAAAACCCCAC 133599
133598 GATTGAAGTTAAATATATTTTATTATGCAGGAGCATAAAATGGCGGCGTTAATTCTGAGCATACGCATCA 133529
133528 ATTGATTTATATTGGCACAAGTTATTTCTGCGCGTACTGTAAGCGGATAAAAGTTTAACAAGTGCTTTAT 133459
133458 CGGTATTTCCCGATCAAATAAACTAATATTCGGTCTTCTCTCACGTACTTTTCCCACGGTACCACCGGCA 133389
133388 CTAACCGCTACTAATAATTTACAGTAATTGATCGAGGCGGCAAAAGAAGCAAACCGGCTTTCGGAACTTA 133319
133318 ATTAACTTTTGGCGGCGCAATTCAAACTTCGAATGCGCCGACCGATTCGCAGCTAGTTGAACAGATTCCG 133249
133248 TTGATAAATACCAATATTATCAGTATTATCATAGGGTTTGCCGAGACCGTGGGAACGGGCTTTGCCTCTA 133179
133178 GAACTCGGTCTTTCTCGTTTGCTCGGTATTTGTTTTCTACCGGGTAGGTTTATTTGAGCCGCTAACATGC 133109
133108 CTACATTCGGTGAAGTGACTTCTGCTAGTATGACAGCAATGCGCATCGCTCCAGTCTCTAACACTTTGAG 133039
133038 GACTCCTTTCGTCAAAATTCAAGGTGAATTTCAAATTTACGAGATATTTGGCAAATGTTAAACGTCTTTT 132969

132968 TTTCTTACAACTACTTTTCTAAAATTCATATTTTTTTTCTGTTTGGTGTAGGTCATCAATATTCGCCA 132899
132898 TTTAAAAAATAATTAATCTGTCGTACATAGATTTATTATACACTTTACACAAGTTAATAATAAGTATCTT 132829
132828 ATGAGGTTATTATATACTATACACAAGTTAATAATAAGTATCTTATGAGGTTATCGTTGCTATCAGGAAA 132759
132758 AGACAGAAGTTAGTTGTGAACAGAAATACGTTTTCCATCAGTTTTGCGGTCTTACCCATATCTGTGCTCT 132689
132688 CTTAAAGCCGCATTGTCACCAGTTTACTTCCGATCGGTTACCTAACAATATCCGATGATTTTTAACGGAA 132619
132618 AACGCGAAAAATATTTCAAAATATTGAAAGCAGTGTGTCTATTGGAAGTTTCTTTGAGGATGTGATCGAT 132549
132548 AATTTAGAGCGGGTGGCCTGTTAAGTACATCGAATTCTAATATATTCTTTTGTCTTTTAACGGTTAAGGT 132479
132478 TTCCGTCGGACCTCCGGAAGTAAACTGGTGACAATGCGGCTTTAATCGTGAGCTGTTGAGAGTGCAAAAC 132409
132408 AATCCACGCTGTAATGTTAGCGTTGTATTTTGCTTATTCGCAGGTTTCGGTAAAAGAAAATACCCGCCGT 132339
132338 CGACTGACTCTGGCTCAAACAGTCCCGGCAGCGACCAGAGTACATCCACAAACGGACTCAAGCGCCGCGC 132269
132268 CCTGGATTCCCCGCCTCTTCTCACAAACGGCGCCAGCAACGGATCCGTACCGCTTCCACAGGACGTTTTA 132199
132198 TGCCGTGCATTCCCCAACTACACTCAAGCGGCGCTGGATATCGTCCTCAAGGGCTGTAACGGTAACCTCA 132129
132128 TGCACGCGATTGAGGTGATCACACAGTGCGAGACCACTAGTTCCAGACAAAGCCCTCATCTACAGCCCCC 132059
132058 GGTCATTCCAAGCAGCGGGGAGCCTGGCACTATGAATCCTCTAATACCACCCTTGTATAAAATGAACTAC 131989
131988 ATGAATGGACAATACAGGTTTCTGATGCCTCCCCCTGGTATGATGCCTTTGGGTTTCTCCATGCTGCCGC 131919
131918 CTGGTGGTGCCCCCCCTTATTTTCCGCCGCAGTCAATGAGTACACCTGCTTACGAGGACGGGGCAAGCAA 131849
131848 TAGTCACCACACGGAGAGACCGCTGGACGGAGAAAGAGATACCGCGGTCGTACCGAAGACGGAGCATGTC 131779
131778 GAGCCAAGTGTGTCGCTAACGAATGGGAACGCTTCCTCGCCAAGAAAACGCTGTAAATACTGCGTGACGG 131709
131708 AACTGAGAGATAGTGATAGGGCCTGCCACCAATGTGGAAGACTCGCTCTATAAACCTGAAATATAGCAGA 131639
131638 GAAATTAGTGCACTACTTTACTTGTGGAGCGTGGATGTTTTCCCGTTTGCTCCAATGATAGTTTTGATAA 131569
131568 AGTATTTCTAATATTTTATATGAATTCAAAGGACTCGAAAGAGCGAAATAATTTTATTTTTAACGTCTAG 131499
131498 TAATATTTTCTTATGATATTTTATTGATCGATATCGTTGTACTAATAACATTTGATGTTATGTTAGTTCT 131429
131428 ATTACGCATTATTTATTATTTTATTTATAGCTAAGCCTTTTATCACTGCATTTGCAGTTTCTTAAGAATC 131359
131358 TCATTTGAGAGTTAAAATCACCAGATGATTAAATTTTGCATAGAAAAAAATACTTTTGGTAATAAATTGA 131289
131288 AAAACATGTGACCAGCCTGTTTCTTTTGTTCCTTTCCTCATTGCAGGCCCGTACGCAGGATATTTCTTGG 131219
131218 GAGGGGGGTGCGAAATCCGAAAAGTGGTCCCATTTTCTGTGTGTGTGTGTGTGTGGGTGGGTGGGTGGGG 131149
131148 GGGGGACAAAAAGGGAT 131132

DMRT D
795852 AAGAATGACAGTGACACAGATGCAATTTTGCGCCAGCCAATCACGGACGTCGGCTAAATGAATTTGGCAA 795783
795782 AAGACAGACAAAGGCGTCGAGACGATTAGTTGTCGCAGTAATCAAGTACTAACATTGCTAGTTTGCTGGT 795713
795712 CAGCCAATACGACTGTGCTTTTCAAGATATCTGGTCTAGTTGCCCGGGGCATTTGGAGTTATGAGCGAAT 795643
795642 CTGCGTACATGATACAGGGGAGTTATCCCGAGCGAAAACCCCCTAGAATGCCGAAATGCGCGCGTTGCAG 795573
795572 GAACCACGGAGTCGTCTCCTGGCTCAAAGGCCACAAGCGATACTGCCGATGGAGAGACTGCAACTGTGCC 795503
795502 CAGTGCACTTTGATCGCCGAAAGGCAGCGAGTTATGGCAGCTCAGGTAGCACTACGAAGGCAGCAGACCC 795433
795432 AAGAAGAGAGTATGCGAGTACAAGCCGGTCAAATACCTTCGAATTACGTCCCGTCATCGCCACTGCCTCC 795363
795362 AGCGAACTTCACCGAGCTCACACCGCCCAAACCATCGCCAGGTATAAGAACAGTTCACTAAAACACTCTA 795293
795292 GCGCCCCCTTTCTCATTTTATTACACCACCCACGTTCACTCAATCAATGTTAAATTACTTTGGGCACTCG 795223
795222 AGCAAGTTTATAACATGTAGCAAATTAATATGTATTTGTAGTTTCTTTAGTCTGTCATTTGTGGGCGCGA 795153
795152 TTGTGTCAACAAAAGTTCTTTTCGAGCGTGTGTGCGCAAATGAATCTTAAATGGAAGAAAGGCCCGGGTC 795083
795082 AAGTCGGCTGACCTGAATTGTTAAATGAAATACTTCGCCTCTGACGAGTTAAGCATTCCGTGTTACAAAC 795013
795012 TGGATTGCATATTCATGAGAGAAACATTTTTACGTTACGTAAGCAATATCTTTACTTCTACAAGTGGGTA 794943
794942 CTTGAAATTTTGAGAAAGTAACTAAAGAGCAAAGCTTTTGTTATTGTCGATACATGCGCAATAATTCGGT 794873
794872 GGGAAAATTAGCAGATCGCTTTCATATTTGTGATTGAGTACGCTTGGGTGAATGGACCATTCCAGGACTA 794803
794802 ATTGAGCAACATGTGATTTTCAATTTAGGTGTTTACTTTGTTGCAGAAATTGAGAATTTAAGTAATCGCA 794733
794732 ACACCACGGTCAAGGAATCTGATTTTCAAATGAGGATAAAAGACATATCTAGATTATATAATCATTGCTT 794663
794662 GGAAGAAAAAAACATATTTCTTATCTGAGCGCAGTTAGCTTTTTTTATATAGCCAAGGCTAGACAGCGTT 794593
794592 TTCTTTTTTGCATGTGTTTTTTTAATAACCTTATACTGCATACATAGTATAGTTTCTTACTCTACGTGAC 794523
794522 ATTAAAAAAGATTCTTTACAAAGGAGAATACACACCCTTTGTCTGTGCGATTACTTGGAAGCCTTAAACA 794453
794452 ACAGTGACATTCAGACTTGGTCACCATTAAACCTGCTTCCTATGAATTGTCAAATAATATGAGAGAGTAA 794383
794382 AGTAAAGTAAACAACCTGTGACAGAAGAGAACAGTGGAGCATCCATTAGCAGAGACTCAGGTACTACACG 794313
794312 CGCGGAGGCCGGCTGTAGGCAAAAATATTTCTTTTGTGCGCGCGATTGTTTAATGGTTGCAACCGGAGTT 794243
794242 GTGATGTCTCGTTTAGTGTTTTGGCACATTGTTGTAGAAAGAACCTGTCTCCTAGCAAATGATAGACTTG 794173
794172 TACGACCTGTCTTTGCCACTTTTATGGACTTCCGATGGAATAGATAGACCTTTCCACTGTTGCTGACTTC 794103
794102 AGCTCTTATATTAAGTTTGAGAGGTTTAGAATGATTTAGCATTTTGCTAGCTTGCTTAGTTTAAAGTTTA 794033
794032 CACCTTTAATTAGCATTCCACTTCACGAAGAATTAGAAAAGGTTCTTAAAGGTAACAATCTTATTGTGTA 793963
793962 CTCTCAATTTCAAGAGTGCCATTAAAAAAGCGTTTAATTTTAAGTTCTTAATTGTCGAAGCTTTTTATCA 793893
793892 TCAATATTAGCTCTTGACTTTATTAGATTCCGATCTATCTTCCTCATATCACTCTATTGGCAAACATTAT 793823
793822 AAAGGAGCGTTTGTTTAACTCAGCGCGGACGGAATCATTCTCAATGTGCGAGTCTTATATCCAAGCAACC 793753
793752 CTGTCTCATTTTTCGAGTTATTGCTAAAAGCGTGAGATGATAAACCGATACATATTTTATTTGCTATTGT 793683
793682 ACCCTAATTTCCGGAATTTTAGACTATAACGAGATATTGTCATTTATGCACTCGGATGGGAATGTTATAA 793613
793612 TATTTCCCGTTTTCATCCGAGTGCATAAATGACAATGTCTCGTTAGAGTCTAACATTCTGGAAATTAGGG 793543
793542 TACAATAGCAAATAAAATACGGCCCGTACCCAGGATTTGTCTTGGGGGGTGCGAAATCAAAAAAAGTGGG 793473
793472 CCTAACATTTCCGTGGGGGGAGGCAGTTCTCTGATAAAAATCGAACGTTTTATAACATGAGTGTACACCA 793403
793402 AGATATTGTATTCTTTATTTGAGCTATGCGACATATATTTCAACAGAGTTTTTAGCTTTCCGATATAATC 793333
793332 CTAAGATTACTTTACTGCCGTGCAAGTGAAAGTTGTTAATGCCCACTTACAAAGGTAAAACGGAGGGATT 793263
793262 ACAGTTTTTGTTGTTGTCGTTGTCTTTGGGCCTTCCGATATATGTGCTTAACTGCAAAGCATGCTCTTCT 793193
793192 GTACTCAAAACCGTAAAGTTTCGAAATTACGTTCCATGCAACTAGCTTTCAAATTCCTTTTTTGATAGGC 793123
793122 CGATATCCACGTGGGCTTTTTATCGATGTTTTTTTTTTCGTTAGTAGACTTTTCAAAAAGGCTTATTTCG 793053
793052 GATGGGCGTCTTCAAACTCTTTTCGCTCCCTCGTAAAAGTACACATAGTTCTTTCAAGGTAACTGCCCTA 792983

792982 TATCCCAGTCTAGTGCTGGGAAAGTACGACCACTTCGTTCTTTGTTTATAAAACTCTACGTGTCTACCCT 792913
792912 TCTTAGAGAAATAATCAAGACAGCGGTATAGTACACGTGGTTGATACATTGTCTGTTTTTCACCTGCCGA 792843
792842 TCTGTTGGGACGATTCTGCGTGTGTTCATTAGTGTCGGGCAATCAATAAGCGGCCCGAAAAATCGGTGCG 792773
792772 TTAAAATCCGCCTTATAGAAATATGTTCGCCATGCGCTAAAACATCTGAACTGCTTTTGTTTAGTCGAAA 792703
792702 TGTTATAGGTCAAAAAAGGCTGGGCTTACTTCTATCAAGCTTGTGACGCGTGCTTGAGACATGAGACATT 792633
792632 TCTTTTTGCGTTAAAATTATTTTTGAAGGATAAATTCCTTTCTTTTCTAAGAGTTTTTCGAGGGTGGAGC 792563
792562 CTTCCACGTTTTGTTTTTTATTTTTAATATTTAACTCCCTCCCCCATAAAAATTTATGCGTGACTAATAT 792493
792492 CACTTCTAATGGATCGTTCCTAATCATCTTTCTTTCTTGTAGAAAAAGAGACAGAATTCCGACAAGAAAG 792423
792422 CTTCAGTTCTCCTGAGTCAAAGCTAACCGAGGTAAAAATCAAGGTTGAACCTATCTCTGAATGCGATCAA 792353
792352 AATAACAACCTAGAAAAAGACCACGAACAACAACCGAACAGAAAACGAAGCTTTAGCGACAGCGAACCCG 792283
792282 AGTACAGTCCGAGAGTTTCCGAATCGCACAGCCCCGTGCCTAAGAAAGCCGTCATATCGGAGAGTAATCT 792213
792212 CGTAGTGCTGCAGAGAATCTTCCCTCATCAGAGTCGAGCGGTACTGGAGCTAAGCCTGAGAGCGTCAGGA 792143
792142 AATGATCTAGTCAAAGCCATAGAATCACTTACCCCAGATGAGAACAACAACCTCCGTCCCTTCCCCTGTC 792073
792072 TGTTCCCAGCGCGAGGTCATGCGCACCTTGGCGTCCATCCTTTGCCACCGAGTGATACCGAAGGTGCGAA 792003
792002 GTCAGCATTTTCCACCATTGCCAAGTCAGGGACATTTATTCACCCTCGAGTCATGAAACCAAAGGAACAT 791933
791932 TATATCAAGAGTCCTACGTCTAAGAGCCCCCCATCAAAGAGTGCCTTTCACCCTCACCCTAGTTACGCTG 791863
791862 CCCATCCAATAGCTAGTCCAACAATGAAGGAACATTTCCACTTCCCGTCTGTACCCTCTCACGGGTACGT 791793
791792 TTATAACAGGTCAGCTACAGCTGCTCTCCTGTCGCTTAGCAACGCACAGCAACGAGGCGGTGCACCGCCG 791723
791722 GTTGAACCCGCGATCTGTGTTGAGTGCGGATTCGTGGCGAGACCTGAAGATAAATTCTGTAGTGAATGTG 791653
791652 GAAAGCGGATGAGCTGATTGGACATGTCCAGCGTCACGTGATGAGTAGATGCCGAGACAGAAACATTGTT 791583
791582 TTTACTGTTGGTGAATTATTGTGGTTGATTTATGTATTATTGTATGATATATTTTGTTATCTCTGTAAAA 791513
791512 AGAGTGCTTACACTTTGTCCCTAATAGTTCTTAGTTCTAAGGCATTATTTTTGTATTTGTCTTTTCTTAA 791443
791442 ACTGTTT 791436

DMRT C
50386 AAAATAGCAGTCACATCGCTCATACAGCTATTCAAAAGTAGCTTAGAAAGAACCAATCAGAACCTTGAAG 50317
50316 TTACAAAGAATTCACTCAAATATTACAATAAGTCTTTTAAGTGTCGAAAGCGCTCAAGGTGATATAGCTC 50247
50246 TGCTAGTGTAATTGCATTGAAAGCCGCAAATTGCAAAGCTAACAGTTGCATTCGAGCGGGCAAACCGTCT 50177
50176 GGTCCTCATCGAGCAGCAATGTCGGAAAGCGCTTCGGGCGATGAAAATCTAATTCGATCCCCCCGCTGTG 50107
50106 CCAGGTGTCGCAACCATGGTATAGTTTCGTATCTCAAGGGCCACAAAAGATACTGTCAATGGAAAGATTG 50037
50036 TACGTGCACCAAATGCAGTCTTATTATTGAGAGGCAGAGGCTAATGGCGGCACAAGTTGCTCTCAAACGA 49967
49966 GACGAAGAAGATGCAGTTGGGTACAGAGAGATGCCCATTGAGCCAAGTATGATGTACCGTGCAAATACTG 49897
49896 AAGAACTTCCACCACAACCGGACAGAAATAATAACATGAGAGTTCGTAGCTTTGGCAGTTCATCTAGCGA 49827
49826 TCCTGCATCAGAACCAAAAGATGTGAAACCATATTCAGAAGGTAAACTATTTCTATACATATTTTTATTA 49757
49756 CTTAATTTGGAAATGTGTGATTTTTGACTTTCTGAGAAATGGTCCACTTATTAAATTTCCATACCCAAGT 49687
49686 TTACGAGAATGTCATAGCTCATATCAATCAGTCTGGATTTTCACCTGGATCGCAATGTAACTTGATGAGT 49617
49616 TGTTTCACTCGCTAAGAGTTTCCCTTCTAAAATCACAGGTGTGATGCAGCCTTGCGAAACCCGCCATTTG 49547
49546 CACCTTAAAGATTTGGCGACATGCTTAACAAATTACGAGACCCATTGAGACTCGAGGGCAAATTGATAAA 49477
49476 TGGTCTAAAACTTGACATGTTCTAAGTAGATCCGTGACTCAAGTGTCACAAAAAGGGGTTGCGAAAAATT 49407
49406 TCAATATACGGAAAAGCGCACTTGAAAATTGACAAAGAACCAGACGGGAGTAACAATGTAGTGTTTTGTT 49337
49336 ACCTTCGCATTCTTACATAGGGTTTTGGATACAACCCCTACAGGTGTATTCAAAGTAAAATCTTCAACGC 49267
49266 GCATTCAAAGGCGCTTGTTGAAACAGAAAAGTAGTTATACCGATATAAGATTCAAACGTTTGTCATTTTG 49197
49196 ACGATCAAAACGCGCACAATGTAAACACGGTTTACTCTTGGTTAATTGGAAACGTTTTTACCAATCAAGC 49127
49126 TAGCGCGCAGCCCGGAAACGAGCTGCCAAAAGCAGAAAAGAAAGTGTCTTTTCAGAGCCCTAGTATTAAT 49057
49056 TGGCCTGTCGGGGTTTATCCCTAGTAGATAATGACAGGGTGTATTGTGCTCGAAATATCCAAGCACTGGC 48987
48986 AAACAACGCGGGACAAGTTCGGATGTAGGCATAAGCTCTTAAAAATTTCCAGTTTGTATGTCAAGAACTT 48917
48916 TATTATGTTTAGGCAATATTATTCCCATGCTCTTTAGCCTAAATTGCGAATAAGGAAAAGGTAAATTTGT 48847
48846 GCATTAAGAAAGATCGAGGTTTAAATTGAGGAGATATCAGGGGGGAAATCACAGATGCAGGTACTGAAAT 48777
48776 TGGCTTTGGTTCGGCCTCGAGGACAATTATTTATTTTTGTGAAAAGTAATATTTTTCATTACTTAATACA 48707
48706 GGTTCGCCGGCAAAGCGGCCTAAACTTCCTGACATGGAGGCACAGTTTCCCTCACCTCCCGAGGCGGGCG 48637
48636 ACGACAATGTCTTCTACGATTCCGACGTCCCCAGATCCGAACACGGGCGAACAAACCAAGGCGTTCCCGA 48567
48566 GCGCTGCACGAACGAAAACGGTGGCTCTCCGACGACAAGATACGACGTCTCGCCCAGCAATGGATTTCAT 48497
48496 CAAACCGCCGAACAGTTAGTACGTGGGCGAATTCCTCCAATAGAGCTCCTTACCAGGCTCTTTCCGACGC 48427
48426 AAAAGCGTAGTGTCCTGGAACTGATTTTCAAAGGTTGCCATGGCAACACACTTCAAGCGATCGAGTGTAT 48357
48356 CTTACCGAGCCACGAAAAAGCGATGTCGATACTAAAGCAGCCCGAACCCGCGTATGTACCGTACAACCCG 48287
48286 AACATGCGCCCTAAGTATCACGCGACCAGGCCCCCATATAGCCCGCGATCACCTGGGTACGCGGTTTATG 48217
48216 GTCAGCAGCAAGGTTACACGTATCCAATTCTGGAGTATGTCTCTCACCCAAGATGCTCTGCGGGGCGTGG 48147
48146 CGGATCCACTGGTGGCAGCGAGCCACCCGAGTCCCCGATATATCACGTACGACCAGCGGCACCACCCACG 48077
48076 GAGAACGTGCAAGTCGGGAAAATAGTGGGCCGCGTATGCTCCAAGTGCTCTGTCAAGTGCTCAACTAACA 48007
48006 GTAACTTTTGTTATGCCTGCGGGACGAGCTTCAAAGATATGTAACGGAAGCTGTGACATTAATGTTAATT 47937
47936 GGAAAATGGATGAAAGAGAACGGCAACCGCATCTTTTTTTATAACAGCTCTCGTAAGAATTGTCTCTAAA 47867
47866 ATTATTTTAAGGCGAATGTCGTTGATACAGCGCTTTTAAAAAATACGTAGATCCTTCATATATGGTAGTA 47797
47796 ATGGTTAGGGACGCTATAGTGTACATATATGCATGACCTACCAGAAAGGTTGCATTTGAGAATCCATGTA 47727
47726 GCGTTACCCGCAGTTGTCCATGGGTAACGTATATATGGCTGAACCCTGGTCTGTGAAAGCCCTTTATAGG 47657
47656 CACATAAGAATGCTATTGCTTTCTAAACCTTGGATTGATTTTATTATTACTAATTTGTTACCCAGGAAGC 47587
47586 AATGCGGGTTACGACCCATGTATTCTCCGAGTGCAACGTTATCTTGGTTAAACCAAGAACTCTCCAAAAA 47517
47516 TATGGAAAAACCTTTTCCAAAACCGTTATAAAAGATAATCACCTAACGTTTGTTTTAAGCTTTTTTGTCT 47447
47446 AACATCAGCACTGCATACATTTGTTTTATTGAGATGTGACTATAAATCTATGTTGCAATAAACAACCAAA 47377
47376 ACTCTTGCAAAAAGTTTCATGGAACTAGAGCCTTTTTAGCACTAGTACAATGATTTTGATACCTTTTTTC 47307
47306 TAGCCGCATTGTCACCAGTTTACTTCCGGAGGGCCGACGGAAACCTCAACCGACAAAAAACAATGGAATC 47237
47236 TATTCCAATCTGCGCTATGACAGGCTATCACCATTATCAGTTACATTCTCAAAGAAAATTCAAATAGACA 47167

47166 AACTAATTTTGACATATTTTTCGTGTTTTCAATTGTTACTTAATCGACCGGGATATAAACTGGTGACATT 47097
47096 TAGACCACTTTTTTAGACTGGCTTTAGACCACTTTTTAGTACTGAATGCTCTTCTTTCTGTTTGAATGAA 47027
47026 AGATTTTAAAATCAAGATAATACAAATGCATAACTTCGTAAAAAAAATCATTTATTACTCAAATAACTAT 46957
46956 TTTACATCTCTGTTCAAATTCATATATTGGAAGTGTTGTGCGAGCTCATACTATATATTAGGTAACTTCT 46887
46886 CCACTCAGCTCTATTTTGTAAACACTTCTAATCACAGTGACAGGCATGTACCCAGGATTTTTCTTGGGGA 46817
46816 GGGAGAGATTCTTCCAGGGGAGGGAGTA 46789

DMRT H

77856 AAGGCAAGATAAAATAAAATTCCGATTTTTTTTAAGTAAAAACACCGTGAATCTAATAAATGCCACAGGT 77787
77786 AAACTATTTCCCCTTTAATTATTCAGCGGCCCAATTATAATCAGGTAAATAGCAAGGTACCTGGAAAACC 77717
77716 TGTGATTACTTTCTCGATAGTAATAAACGTGCCTTCAGTCAAGAACCACATCATAGCACGATGGACGACG 77647
77646 ACCACACAAGCAGTCAGAGGACGCCCAAATGCGCGCGGTGTAGAAACCACGGGGTGTGTTCCTCGCTCAA 77577
77576 AGGCCACAAGTACTACTGTAGATGGAGAGACTGCTTGTGCACCAAGTGTCTCCTCATCGCAGAGAGACAG 77507
77506 AAGATTACGGCGGCCAGGGTAGCGCTACTACGGTACCAGAATACTTCAAGCTGGCAGCATATGCCCGGCG 77437
77436 AGAGGGCTGGGGAGCTTAGTGACGAGTTTATGCCGCCCCTGCTGCTTCGCGGGCCCGAGAGAGCAGCGAG 77367
77366 CGCCTTCCGACAACCTGACATACCAATGACTCAAACGATAACTACAGGCAAAACTCACCAGGGTACGTCT 77297
77296 ATCAGTATTCTCTAAAGATAAACATTTCATTTATGGAAACTTTGAATAACACCGCTATGTATCTAGGGGT 77227
77226 GGGAGCAAGGGAATGTGTGTAATCAAAACAACCATCATATGACCTCTAAAGGCTTGTAATACTTAATATC 77157
77156 ATAACTAGAGTAACACAAAAGAGTACATGTCACCCGCCATGTAGAACAACATGAAAACGTGTTTTGTTTA 77087
77086 TTCATGGTGAATGTTATTTGACTTTTTTTAAAACGGGTACAAACTTTTTTTAAACTATATATAGTGAATG 77017
77016 GATAATCAGAGAAATTAATCCATTAATCAAGGTATTTTAAGTAAAATGTCGACGAATGAATGCAAAGATC 76947
76946 AATAGAATTGTTTAATGGAAATTTCGGAAAATAAAAGTCAAAGGCTATACGTGGAATCGGTGACTTGATT 76877
76876 GACGTCGGTCAAGGACTTGTTCAAATTATTTTAAAAATTCTTAAAAAGAAATGACACAAATACTTCCTTG 76807
76806 AAAAATAATGTTTGGATTGAACAAGATTGAATCTTTTGGCAAATTGATAAAAAAAAAAAACTGCATACGG 76737
76736 CATCTAACCATTTTTCCCTTAAAGGAAATAAAAGCTTTAAGGATATTGAAACTTTATAAACGTGAAAGAT 76667
76666 ATATTTTATTTTAATTCATTCATAAATCATGATTATTAAATCAAGTAATAACATATTCTATTGTGTAATT 76597
76596 TTTGTTTAAATGTGGTATAAAAAACAGGTATAATTATCTTACTGTGTCCATCAGGCACATCGTCAAAACA 76527
76526 AAAGAACGACAGAAAATCTATTTTTTGTTACCTTCAAAGTTATCCCAATTAATCCATTTTACAAGAACGG 76457
76456 GTGGTGAAAGGGTGGTGAAAGAAACCAGAAACGTGTTTTTATCACTTTGTCACTTTTATCTAAAGGGATA 76387
76386 CCCAAACGACGTGCATTCGTAGAGGGCAGGAAATGAAAATGAAAACAAACATTATATAACCTCTAAAGGC 76317
76316 TTGTAATACCTTTTGGGGTTGATATCTCATGATTAGAGCAGCACAAAAGAGTACAATGGGGGTGACATGA 76247
76246 AAACATGACATGTTCATTCGAACGTGACTCGAAAAACTTTTCCACTCAATGTGAATTGACAGAAGTAACA 76177
76176 GGTGCAAGCTAAATAGAAAATAAATGGATAATCAGAAAAATAAATCCATTAACAAAAGGTCAAATTTAAA 76107
76106 TGTTTTTTATGTAATAGATGTTTTTGTTATTCAAAGACTTTATTGTAGTTTCTTGGTGTTACGGAGCACC 76037
76036 AGCACCAAAAACACAATGAAACAAAAGCCAAAAACAAGAACCTTAGAACGAGACAATAGAATAGAGAACA 75967
75966 AAGGCCTTGAGCACCAAAAACAAAGCTCTCCGTAACACCAAGGAATACCCGACTTTATTCATGTTAATAC 75897
75896 CCGTGACAAAATAAAAGCCGGGGTGGGGGGAGGGAAAATGCGAGGTTATTAATATAATTTTAAATGCAAA 75827
75826 AATAAATATGAAATTGTTGGAATTTCCATTTTGAAGTACGGTGGATGGATGAGATTGAATCGCACTCGAT 75757
75756 CGACGTAATTTAGGTAAAGAAGTGTACACATTCTGACTAAAATATACTTTCTTAGCTGATAGCTACTGTT 75687
75686 GAGTTTTGCGTTGAACACTTTCAGAAAATATAAAAATTAAAAAAAAGACTTAATGAACACGAAAGATGAC 75617
75616 TTGACATCATTTATTGCTATCATTTCAACTTGTTCTTGAAACAGAATAATTAATTTAAATAATATTTTTAT 75547
75546 TGTAGGAAAAATTTAAAAGGCAAAGGTATCTGATGATTAAACAGATTAAACATCAGATTAAACAGGCAAA 75477
75476 TCGTCAAAACAAATGCACGACAAGAAAATCTGTTGCGGGTGATTTAATCGACCAAAACCGTGAACAGTAA 75407
75406 TGCTCATAAGGAAATGACTAGAAGTGGCGTGGCTGTACCCCCCAGTTTTCTAAATGTATCTGTATTTCTC 75337
75336 CCCCTCCAAGATTTCGAGGTCTGTTAAGGCCCTGATCCCCCTGTTACTGTTTATCTAAGAGGTACTTAAA 75267
75266 CAAACATCATTTTCCAGACGATATCATTGTGCCTCCGATGAAAAGACCGAAACTTGAGAAACCTTCGACT 75197
75196 CTAGATGAAATAGTCACAGAGCCCTCCAACGAATTTAAGGGTTTCCAGGTGATCCAAAGTCCTACAAACC 75127
75126 CACCCTTCCCTACAGAGAAAGACAAGAGCGCAACACCAAGTATCCATCAAGATCAGGGGGGGTCAAACCA 75057
75056 TGCAAGGCTTGACGAAAATACGCTACAGATTCTACACCAGCAGCTCATGTCTGTAATGCACTATCAACGC 74987
74986 CGCACCCCTCCCCTGCCAGTTGACCTTCTAGCCAAGATATTTCCCTCAATCGACCGTCGTGTGCTTGGGT 74917
74916 TGTTGCTCCAAGGATGTGATAACAATGTCGTCCTGGCGATAGAATGCATCGTCAATAATAATACCACCTC 74847
74846 ATCAGGCCGCCTCTTCGAGTTCGAAAGACATGCCGCAGCCTCTAGTGATAGCCCCGCTGTCAAGTTACCG 74777
74776 GAGCAACGTCCCATAAGAAAACGTGTTACGCCACCTAGGTTAATCCCTGACGCGAGGGGCGTGTCCCACG 74707
74706 AGGAACTGTTCCCAAGGTTTCGCCGACCGGGCAGTAGGGACTGATTCTGGCACTGCTGAACAATTTGTTC 74637
74636 GATCTTTTTTTATTATTAATTAACTGTTTTAAAAGAATGTAGAAGCCGGAAATTTCGAAGAACGATGTAT 74567
74566 AAATAATGGATTTCTGAATATATGACTTTATGTGTGACTTTGAGGGTGTTACCACCCTCAAAGTCACACA 74497
74496 GGGTGTTACGATTTTTTTAGCTTCGATTTGAGGG 74463

DMRT I

1064019 AAATACTTTAGCGTCGTTTTCGAAAGTGCTAGATTTTTCTCTGTGTTCTCGTAAAATCTCGCATTGACAT 1063950
1063949 TCGCCACCCCTTTTATCCTATGCCCAGACACGCCAGCCATAAGCGCTACGCACTGACTAGACCACACACA 1063880
1063879 GACTTAAACAGTTTAGTCAGACCTTTGCCTTCCCATTTGTTGCGTCATTTTGGAGGAAAGATGAGCAAAG 1063810
1063809 AATGGTCCGTCAAAGAAGATAACAACCTGCTGCGCACTTTTAAAAGCAAGACCTGCAGAAAATGCCGCTC 1063740
1063739 TCACGGTAAAATAACCCCACTAGAAGGTCACAAGAGAAACTGTCCTTACATAAAGTGCGACTGTCTCTCT 1063670
1063669 TGCACTCGACTCGACCGACAAAACCTTGCATCGAAAAAAAGACAACTAGACAAGGCACACGAAAAAAGGG 1063600
1063599 AGCCAAAAAAGAAACAAAAGATTGAATGCAGAAAGAATGGTAACGTGCGCGCTGTCAGCATGTAGAACAA 1063530
1063529 ATTGCAGCCTTTGGCAATAGTTTTAATATTTGGTACTTCTAAAAATTCTTGTTTTCAAATTTTTTTGTTG 1063460
1063459 CCACGTCGTTACTTAAATTGTCTAGTGAGTGTAAAACCGTTTTATATAATTAAGCCATAACTAAAGCAAA 1063390
1063389 TAATTCATTCTGAAATCAAATTAAAATATGATGAAGCCTTCAAATAAAAAGACCAAAGAAATTTTCTAAA 1063320
1063319 AAAACGATCAAGCAATTCTTGAATTCTGGCCAGTAGGTCTTTCTGGTCTGTTTCTCTCGCATGCGTATCT 1063250

1063249 GAAAGTACAGCGTTTAAAGCTCATAATGTGTGTAGTTCCAGAAAATATCCATAAGCCCCAATGTGGGGGC 1063180
1063179 TAGAGGTGTGACCCCCCACCAACATCCTGGAAAAAATAAATACAGTAACTGTTAAGGAAAACGGAATTTT 1063110
1063109 TTAATTCAGTGAAATGTTTTCCCGAGCACATCTTTCGATGTATGGTATGATTAGATGTCTTTTTTTGTTA 1063040
1063039 TTTCTTGTTTTCAAAGAGCGCAAAACTAAGAACTCGCTTAATTAAGAGGTTTTACAGAGAAAGAATCAAA 1062970
1062969 ATCAAATCCTGCATGTGGCACAAATTCTGGATGCGGGTAGCGACCAGAAACGACAATATTTTTTGTGGCC 1062900
1062899 TTATTTTTAGTTTATATTCAAAAGACTTTATTTCCATGTTTTCTTCTCAGGTCTGGACAACACGATCCAA 1062830
1062829 GACATGATCCTCCCTAACGATTCTATGCAGCCCTTCTTCAATACTGATTTGTCCCCAAGAAAAAGGGAAA 1062760
1062759 TACAGGAGGCTAAATCCCTGGTGGCTAGTGTTCAGGGCAAGCTTGAGGTAGCTCCTCAGAATCACTTTCA 1062690
1062689 AAGCATGAACAACAAAGATATAGGGTCGCCCCTGCCCTCTTCTCATATTACAAATGATGCAATTGGGCCT 1062620
1062619 GTACTGGCACAACCCACTGCAAGAGGGTTTGGACAACCAACAGCTGTGGACATAAATTCCCTACCAATAC 1062550
1062549 CCCCCGGGGATATGCCATCAGTGACAAATCACCTTCAACCAGTTCAGGACTCACACTTTACAAAGTCCAT 1062480
1062479 GGAAGAAAATTACTTATTCCCTCCTGTCCATGGAAGCAGTCAAGCATTGAACTATGTTAATGACACTATT 1062410
1062409 GTTTTACTACCCTATATACCACCTGCATTTATTGCCCAGTATAATGCAATGGCTTTGAAAACTCGCCTTT 1062340
1062339 TGCAGTCCGGCAACAGAGATGAAAAACAAAATGCAAGTGTGGTTTACAAAGGCAGTTATGATGACTAAAT 1062270
1062269 GCAAAAGTTGAAGCATTGTTGAAATCTTTGTTGGTTAGTAACACAGAAATCAGAACGTTTAGAAAGGCTG 1062200
1062199 TTTCATACCTACTGTCAACTCTCCCGTGTTAGGTGGGAGTCTCAAGATTGTTTACCTCTTCTGCTGCTCT 1062130
1062129 CCCGCGTGTAACACCAAATCTGCTTTTTAGCGATTTTTATCGCTTTAAAAAATCCTTC 1062072

DMRT J

103379 TTACTGGCTACTAGCCATGTGCTCGTTAAAAATCGCCCCCTCTATACCTTGCAATCCTTGTAGACCCTGT 103310
103309 AGGTAAGAATCTAATCCTTTATCTCTGCCCTTCTTTTCTTCTTAAATTGTAAACGTTTCTTTACAAAGTC 103240
103239 AAAGAGGAAAACTGTCTTGTCAATGTTGTCAATCTGCAGACGAGAAATCTACATTGTGAAATGGACAAAC 103170
103169 TTGCAACAAAGTCAGATGCCCCCAAACCACCAACTTGCACGCGGTGCAGAAACCATTTCGTTTGGTTTGT 103100
103099 CCCTCTCAAGGGGCACAGCAAGGTTTGTCCTTGGAAAACTTGCGAGTGTGATAAATGTATTTTGATTAAG 103030
103029 CAACGAGGAGAAACGAACAGAAAGAGATCGGGAATGACTACAAGCAAAGTAATAGCCGAAGAACATACTA 102960
102959 AAGCACAAACAGGTAAGAAAATGCGGGTATGTGTGGAAAAGCGGCACAGATACACACTTAAGGTAAAACA 102890
102889 CACATTATTATGAGCTAGAGACTCTTCTCATCTGTGCGTCTGGTTTTTTCCATTTGCAATCAAAGATACG 102820
102819 TAAACTTTTCTTCCATCCAGTGAATGGGTTTATTTAGAAACGACAAAACATCGCCTATCTTGTGAGCATC 102750
102749 ATTTTCGTCGTGTAGAACACTTCCTTTAGGGGAGAAATAGGTATTGGTTAAAAGGTGAAATAGGACATTC 102680
102679 CATTATGTATCCGTAGTCCTCCAAAATTGGACATGTCAAAATCGGGGTGTTGGAAAAGGAATTTGAGAGG 102610
102609 CTAAAAATACAAATTAAAAACCTCGACAGGTGTGATTAAAAAGTAACGTTTCACAAACCTAGATTGAGGG 102540
102539 ATTTGGACGGGAAAAATGAAATTCCTCAAATCAAGACAGGTCTTAGTCTAAGTCTAGGGGAGGGGGGGGG 102470
102469 AGCAGACAAATTGAATTTTTGAAATTTAATTCCCATCTCGTTCCTTCCACAGAGACGTGGAAAGGTTGCG 102400
102399 GATAAAATAATGTACCATACCAGATGGATATACAGTGCTAACATATAATTTTTGTGCAGGTTTCAAAAAG 102330
102329 AGGAAGACCAAACCAAAAAAAGAAAAGCTATCAACTACATCTACTCAAGACTCTCCTATGCGAGTTAGAC 102260
102259 AGTGCCAGATGCCTTCAATGGCACATAGTGCGACAGAATCACACTGCCCCTGCCAGGGCTCGCCCTATCC 102190
102189 AAACAACATGCAAACCACCTGCTCACCTATCCACGGGCAAAGGTATCCCTATCCTTTGTCGATGCATCTT 102120
102119 TCCTACCCATACATGCCAGCGGCAGCCAGTATCTGCGCTAGTTCAGTACCAGAAATATACTACTGCGGTG 102050
102049 AGAGGTGTCCGGTGGCAAACCTTTCATATCCTCCCCCATACAATACAAACATGCTATGCTCTGGTCCCTA 101980
101979 CAATGGAAGTGCCTCACAAATGAAGCGTGTTTGCAGTCCTGAGATTGAAATGCAGATGTCCACTCAGATG 101910
101909 GAGGTATGTTTTTATACCATATGTATCTTCATATATTTGGAACTAGCCTTACATAAGCCAGCTCTTCACC 101840
101839 CTTTCCTTAGAGGTTATAAAATGTTATACCTTTCCTTTTTGTTTTTTTTTACAGAGTAACAAAGCGCTTA 101770
101769 AAAGCTTGGATCATGGGGACTTCTGGTACCCAAATCCCCCCAATGCTTCAGTGCGTCCTTTGGCACCAAA 101700
101699 AACACACACACAAGGGCCTGCAATATTTGGTCAGGAGAAAATTGAAATGAAGTGTGGAAGATATGCTTTC 101630
101629 ATGAGTAAAAAGAATTAAATTTTAATTATACTTAACAAATGTATAAATAGTCTTCAGTGACAAGGTCTCA 101560
101559 CAGACTTTTTCTGAGACACAAAGTAATTTAAAAGCTGTGTTTTGCTTTTGCAGATTCATATTGTATTCCT 101490
101489 AACATGAAATGTGACAAAGTTTTTTAAAATAAATTTGGAGCCTTTTGCTCTCTTCTAACAATTATGAACT 101420
101419 CAAAGTTA 101412
