# Supplementary material for: Sex-specific and developmental expression of Dmrt genes in the starlet sea anemone, Nematostella vectensis
Source: EvoDevo. 2015 Apr 25;6:13. doi: 10.1186/s13227-015-0013-7 (PMC4433094; doi:10.1186/s13227-015-0013-7)
Supplement: Additional file 2: Table S2. — Primer sequences and the N. vectensis sequence ID used for cloning and quantitative PCR. [file 13227_2015_13_MOESM2_ESM.pdf]

Supplemental Table 2. Primer sequences for amplifying pieces of NvDmrts for cloning and for qPCR.

Gene Name	ID	Cloned Fragment Primers (5' – 3')	qPCR Primers (5' – 3')
NvDMRT A	NCBI: JX559763	GGCGTCATCTCCATCCTAAA<br>ATAGGCGAGAAGGCTGACAA	ATTCTCAAAGGGTGCAGTGG<br>CTTGCTAAGGCTTCCTCGTG
NvDMRT B	NCBI: JX559764	TGCATGTTGATAGCGGAGAG<br>CATCTCCCCCGTAAGACTGA	CATTCAAAGGAATCCCATCC<br>GCGACAGCTTCCTTTTCATC
NvDMRT C	NCBI: JX559765	TTATTGAGAGGCAGAGGCTAATGG<br>TTCCAGGACACTACGCTTTTGC	CTAGCGATCCTGCATCAGAAC<br>GGAAACTGTGCCTCCATGTC
NvDMRT D	NCBI: JX559766	GAGCGAATCTGCGTACATGA<br>GGGAAGATTCTCTGCAGCAC	CAAGCCGGTCAAATACCTTC<br>TTTCTTGTCGGAATTCTGTCTC
NvDMRT E	NCBI: JX559767	GGGCTGTAACGGTAACCTCA<br>TGGCAGGCCCTATCACTATC	ATCACACAGTGCGAGACCAC<br>ATGACCGGAGGCTGTAGATG
NvDMRT F	NCBI: JX559768	GGCAACTCAGCACACAAGAA<br>GTACAAGGCAGATCCGGGTA	GGGCTTATTGATTTCCCATTTG<br>TCGTCAGGGTATCGCTCATC
NvDMRT G	NCBI: JX559769	CGTGAGAAGTATCAACGAGAGATGG<br>AAGTGGCGATGGTGTGTAGATGCG	CGCACTACGACGAGAGGATAC<br>AGTAGGAGCTACAACGCTTGC
NvDMRT H	JGI: Fgenesh1_pg.scaffold.26000011	ACCAAGTGTCTCCTCATCGCAG<br>CAAGTTTCGGTCTTTTCATCGG	TTCCGACAACCTGACATACC<br>CAAGTTTCGGTCTTTTCATCG
NvDMRT I	JGI: 198893	ACTCGACCGACAAAACCTTG<br>AGTACAGGCCCAATTGCATC	ATCATGTCTTGGATCGTGTTG<br>GACCGACAAAACCTTGCATC
NvDMRT J	JGI: 215274	TTGGTTTGTCCCTCTCAAGG<br>ATGGGGGAGGATATGAAAGG	CCGAAGAACATACTAAAGCACAAAC<br>AACTCGCATAGGAGAGTCTTGAG
NvDMRT K	JGI: 207818	CAGATCGGAGCTAAAAGGACA<br>AGGCCACGGAGAGACAGATA	TCGATTCAGGAAAACTTACAGG<br>ACCTATGGGTGGAATTACAGG
